# Etiological involvement of *KCND1* variants in an X-linked neurodevelopmental disorder with variable expressivity

**DOI:** 10.1016/j.ajhg.2024.04.019

**Published:** 2024-05-20

**Authors:** Tassja Kalm, Claudia Schob, Hanna Völler, Thatjana Gardeitchik, Christian Gilissen, Rolph Pfundt, Chiara Klöckner, Konrad Platzer, Annick Klabunde-Cherwon, Markus Ries, Steffen Syrbe, Francesca Beccaria, Francesca Madia, Marcello Scala, Federico Zara, Floris Hofstede, Marleen E.H. Simon, Richard H. van Jaarsveld, Renske Oegema, Koen L.I. van Gassen, Sjoerd J.B. Holwerda, Tahsin Stefan Barakat, Arjan Bouman, Marjon van Slegtenhorst, Sara Álvarez, Alberto Fernández-Jaén, Javier Porta, Andrea Accogli, Margherita Maria Mancardi, Pasquale Striano, Michele Iacomino, Jong-Hee Chae, SeSong Jang, Soo Y. Kim, David Chitayat, Saadet Mercimek-Andrews, Christel Depienne, Antje Kampmeier, Alma Kuechler, Harald Surowy, Enrico Silvio Bertini, Francesca Clementina Radio, Cecilia Mancini, Simone Pizzi, Marco Tartaglia, Lucas Gauthier, David Genevieve, Mylène Tharreau, Noy Azoulay, Gal Zaks-Hoffer, Nesia K. Gilad, Naama Orenstein, Geneviève Bernard, Isabelle Thiffault, Jonas Denecke, Theresia Herget, Fanny Kortüm, Christian Kubisch, Robert Bähring, Stefan Kindler

**Affiliations:** 1 Institute for Cellular and Integrative Physiology, Center for Experimental Medicine, University Medical Center Hamburg-Eppendorf, 20246 Hamburg, Germany; 2 Institute of Human Genetics, University Medical Center Hamburg-Eppendorf, 20246 Hamburg, Germany; 3 Department of Human Genetics, Radboud University Medical Center, Nijmegen 6525 GA, the Netherlands; 4 Institute of Human Genetics, University of Leipzig Medical Center, 04103 Leipzig, Germany; 5 Division of Pediatric Epileptology, Centre for Paediatric and Adolescent Medicine, University Hospital Heidelberg, 69120 Heidelberg, Germany; 6 Epilepsy Center, Department of Child Neuropsychiatry, Territorial Social-Health Agency, 46100 Mantova, Italy; 7 Medical Genetics Unit, IRCCS Istituto Giannina Gaslini, 16147 Genoa, Italy; 8 Department of Neurosciences, Rehabilitation, Ophthalmology, Genetics, Maternal and Child Health, University of Genoa, 16145 Genoa, Italy; 9 Department of General Pediatrics, Wilhelmina Children’s Hospital, University Medical Centre Utrecht, Utrecht, the Netherlands; 10 Department of Clinical Genetics, University Medical Center Utrecht, Utrecht 3584 EA, the Netherlands; 11 Department of Clinical Genetics, Erasmus MC University Medical Center, Rotterdam 3000 CA, the Netherlands; 12 ENCORE Expertise Center for Neurodevelopmental Disorders, Erasmus MC University Medical Center, Rotterdam 3000 CA, the Netherlands; 13 Discovery Unit, Department of Clinical Genetics, Erasmus MC University Medical Center, Rotterdam 3000 CA, the Netherlands; 14 Genomics and Medicine, NIMGenetics, 28108 Madrid, Spain; 15 Pediatric Neurology Department, Quironsalud University Hospital Madrid, School of Medicine, European University of Madrid, 28224 Madrid, Spain; 16 Genomics, Genologica Medica, 29016 Málaga, Spain; 17 Division of Medical Genetics, Department of Specialized Medicine, Montreal Children’s Hospital, McGill University Health Centre, QC H4A 3J1 Montreal, Canada; 18 Department of Human Genetics, McGill University, QC H4A 3J1 Montreal, Canada; 19 Child Neuropsychiatry Unit, IRCCS Istituto Giannina Gaslini, 16147 Genoa, Italy; 20 Pediatric Neurology and Neuromuscular Diseases Unit, IRCCS Istituto Giannina Gaslini, 16147 Genoa, Italy; 21 Department of Pediatrics, Seoul National University College of Medicine, Seoul 110-744, Republic of Korea; 22 Department of Genomic Medicine, Rare Disease Center, Seoul National University Hospital, Seoul 03080, Republic of Korea; 23 The Prenatal Diagnosis and Medical Genetics Program, Department of Obstetrics and Gynecology, Mount Sinai Hospital, University of Toronto ON M5G 1E2 Toronto, Canada; 24 Division of Clinical and Metabolic Genetics, Department of Pediatrics, The Hospital for SickKids, University of Toronto, M5G 1X8 Toronto, Canada; 25 Department of Medical Genetics, Faculty of Medicine and Dentistry, University of Alberta, AB T6G 2H7 Edmonton, Canada; 26 Institute of Human Genetics, University Hospital Essen, University Duisburg-Essen, 45122 Essen, Germany; 27 Neuromuscular Disorders, Bambino Gesù Children’s Hospital, IRCCS, 00146 Rome, Italy; 28 Molecular Genetics and Functional Genomics, Bambino Gesù Children’s Hospital, IRCCS, 00146 Rome, Italy; 29 Department of Molecular Genetics and Cytogenomics, Rare and Autoinflammatory Diseases Unit, University Hospital of Montpellier, 34295 Montpellier, France; 30 Montpellier University, Inserm U1183, Montpellier, France; 31 Department of Clinical Genetics, University Hospital of Montpellier, 34295 Montpellier, France; 32 The Genetic Institute of Maccabi Health Services, Rehovot 7610000, Israel; 33 Raphael Recanati Genetics Institute, Beilinson Hospital, Rabin Medical Center, Petach Tikva 49100, Israel; 34 Sackler Faculty of Medicine, Tel Aviv University, Tel Aviv 69978, Israel; 35 Pediatric Genetics Unit, Schneider Children’s Medical Center of Israel, Petah Tikvah 4920235, Israel; 36 Departments of Neurology and Neurosurgery, Pediatrics and Human Genetics, McGill University, Montreal, Canada; 37 Child Health and Human Development Program, Research Institute of the McGill University Health Centre, Montreal, Canada; 38 Genomic Medicine Center, Department of Pediatrics, Children’s Mercy Kansas City, Kansas City, MO, USA; 39 UKMC School of Medicine, University of Missouri Kansas City, Kansas City, MO, USA; 40 Department of Pathology and Laboratory Medicine, Children’s Mercy Kansas City, Kansas City, MO, USA; 41 Department of Pediatrics, University Medical Center Hamburg-Eppendorf, 20246 Hamburg, Germany

## Abstract

Utilizing trio whole-exome sequencing and a gene matching approach, we identified a cohort of 18 male individuals from 17 families with hemizygous variants in *KCND1*, including two *de novo* missense variants, three maternally inherited protein-truncating variants, and 12 maternally inherited missense variants. Affected subjects present with a neurodevelopmental disorder characterized by diverse neurological abnormalities, mostly delays in different developmental domains, but also distinct neuropsychiatric signs and epilepsy. Heterozygous carrier mothers are clinically unaffected. *KCND1* encodes the α-subunit of Kv4.1 voltage-gated potassium channels. All variant-associated amino acid substitutions affect either the cytoplasmic N- or C-terminus of the channel protein except for two occurring in transmembrane segments 1 and 4. Kv4.1 channels were functionally characterized in the absence and presence of auxiliary β subunits. Variant-specific alterations of biophysical channel properties were diverse and varied in magnitude. Genetic data analysis in combination with our functional assessment shows that Kv4.1 channel dysfunction is involved in the pathogenesis of an X-linked neurodevelopmental disorder frequently associated with a variable neuropsychiatric clinical phenotype.

## Teaser

Hemizygous variants of *KCND1* lead to alterations in the biophysical properties of the encoded ion channel, implicating dysfunction of Kv4.1 voltage-gated potassium channels in the etiology of an X-linked neurodevelopmental disorder with variable expressivity.

## Introduction

Voltage-dependent potassium (Kv) channels represent a diverse group of ion channels encoded by a gene superfamily that includes twelve members (Kv1–12) and about 40 different genes.^1^ The particular physiological role of each Kv channel subtype is determined not only by its cellular abundance and subcellular distribution but also by its specific voltage dependence and gating kinetics.^1^ The three members of the Kv4 subfamily, encoded by the genes *KCND1–3*, mediate a subthreshold-activating, somatodendritic, rapidly activating and inactivating (A-type) potassium current (*I*_*SA*_) in neurons.^2^ *I*_*SA*_ plays a critical role in distinct aspects of neurophysiology, including the control of low frequency repetitive discharge, regulation of dendritic excitation, and action potential backpropagation, as well as synaptic plasticity.^2^^,^^3^^,^^4^

**Figure 1:**
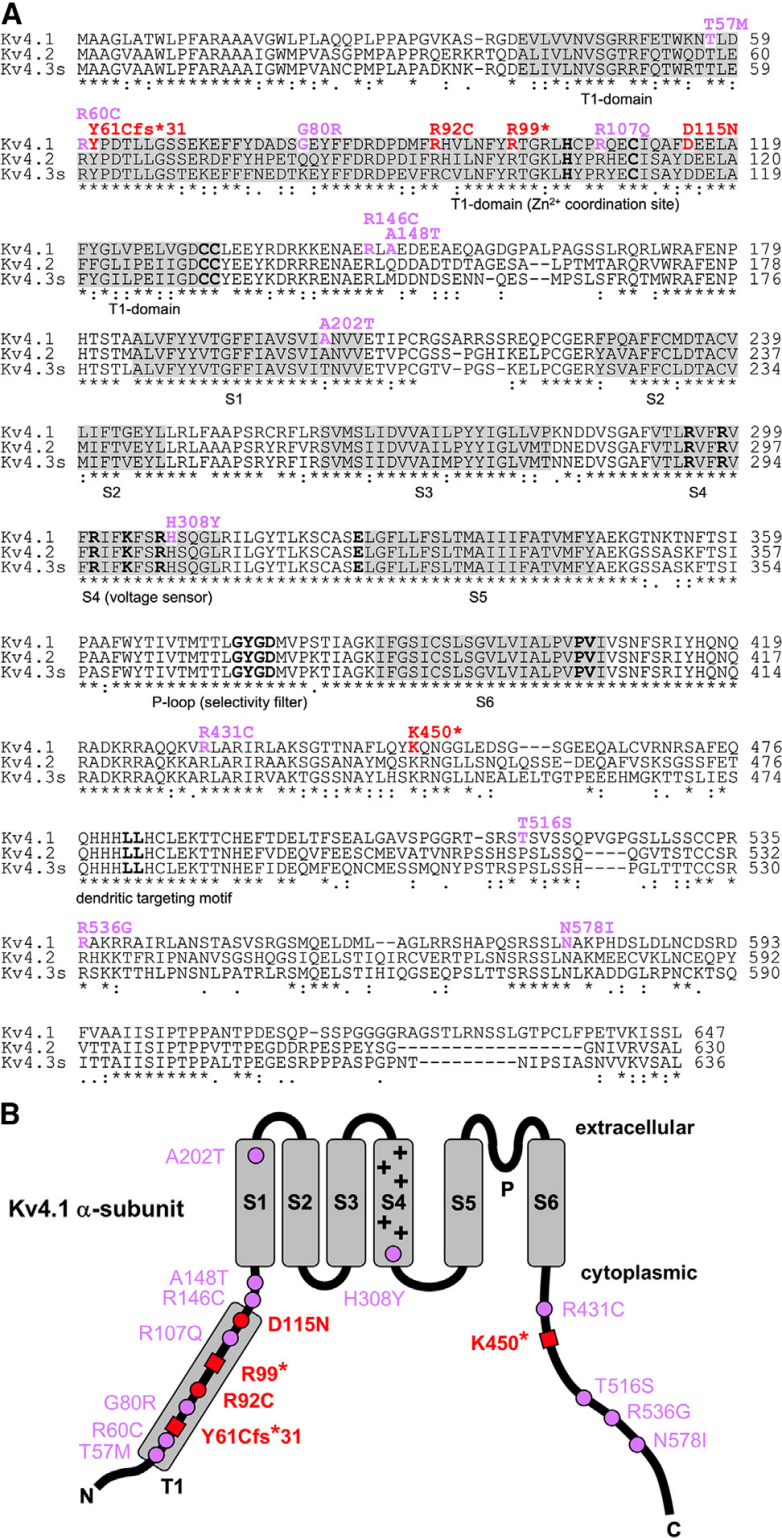
*KCND1* variant-associated amino acid substitutions (A) Alignment of human Kv4 subfamily members Kv4.1, Kv4.2, and Kv4.3s (short isoform). Kv4.1 amino acid residues affected by the reported *KCND1* variants are shown in color, and respective changes are indicated above using single-letter code (red: initially studied *KCND1* group 1 variants; purple: additional maternally inherited missense variants, group 2). Regions highlighted in gray indicate the tetramerization (T1) domain and transmembrane segments S1–S6, as indicated below. Sequence motifs critical for channel assembly trafficking and function are indicated by bold letters (HX_5_CX_20_CC motif in the T1-domain: Zn^2+^ coordination site;^6^ positively charged residues in S4: voltage sensor; ^7^ potassium channel signature sequence GYGD in the pore loop [P]: selectivity filter;^5^ critical dynamic coupling residues, glutamate [E] in the S4-S5 linker, and proline-valine [PV] in the distal S6 segment: operation of the cytoplasmic gate;^8^^,^^9^^,^^10^^,^^11^ C-terminal di-leucine motif: dendritic targeting^12^). Numbers on the right specify amino acid residue positions. Sequences were aligned using Clustal Omega software (http://www.clustal.org/omega/). Residues that are perfectly conserved (^∗^) or exhibit either strong or weak similarity (: or . for a scoring of >0.5 or ≤0.5, respectively, in the Gonnet PAM 250 matrix) are indicated. Note that Kv4.1 amino acid residues in positions 57, 60, 61, 92, 99, 107, 115, 146, 308, 431, 450, 536, and 578 are fully conserved in all three Kv4 subfamily members. (B) Topology scheme of the Kv4.1 α-subunit (extracellular and cytoplasmic side indicated) with six transmembrane segments (S1–S6, gray boxes) and cytoplasmic N- and C-termini. Positively charged amino acid residues in S4 (+) mediate voltage sensing,^7^ and a re-entrant P loop between S5 and S6 harbors the selectivity filter sequence.^5^ The T1 domain (gray box) is located in the cytoplasmic N-terminus. Changes in amino acid sequence are indicated in single-letter code. The two amino acid substitutions and three truncation sites, respectively, associated with five *KCND1* group 1 variants are depicted as red dots (DNVs R92C and D115N) and red squares (PTVs Y61Cfs^∗^31, R99^∗^, and K450^∗^). All other amino acid substitutions (12 maternally inherited missense variants) are depicted as purple dots. Note that all variant-associated amino acid substitutions, except for p.Ala202Thr (A202T in S1) and p.His308Tyr (H308Y in S4), reside within either the cytoplasmic N- or the cytoplasmic C-terminal domain of the protein and do not affect one of the indicated critical sequence motifs.

Like all members of the Kv channel superfamily, Kv4 α-subunits consist of six transmembrane segments (S1–S6), flanked by cytoplasmic termini. A re-entrant pore loop between S5 and S6 contains the highly conserved potassium channel “signature sequence,” which mediates ion selectivity (Figure 1).^5^ The α-subunits tetramerize to constitute functional channels. Characteristic for Kv1–4 subfamily members, the cytoplasmic N-terminus contains a tetramerization (T1) domain, which mediates subfamily-specific assembly, with a critical involvement of specific Zn^2+^ coordination sites in subfamilies Kv2–4.^6^ Within a tetramer, S5 and S6 surround the central ion conduction pathway with the intertwined and flexible distal S6 segments acting as the cytoplasmic gate, while the peripheral S1–S4 segments (especially the positively charged amino acid residues in S4) act as voltage sensors. Kv channel opening and closing is mediated by direct mechanical coupling of the motile voltage sensors to the cytoplasmic S6 gate via the S4-S5 linkers.^7^

Kv4 channels form complexes with auxiliary β subunits, namely cytosolic Kv channel interacting proteins (KChIPs)^13^ and/or transmembrane dipeptidyl aminopeptidase-like proteins (DPPs).^14^ In heterologous cell systems, both KChIPs and DPPs increase Kv4 channel surface levels and modulate channel gating in a β-subunit-specific manner (see Figures S3–S5).^13^^,^^14^^,^^15^^,^^16^^,^^17^^,^^18^^,^^19^ Structural and functional analyses revealed that both the cytoplasmic N-terminus, including the T1-domain, and the cytoplasmic C-terminus of Kv4 α-subunits interact with KChIPs, while S1 and S2 are involved in DPP binding.^15^^,^^19^^,^^20^^,^^21^^,^^22^^,^^23^ It is assumed that native Kv4 channels assemble in a ternary fashion, i.e., with both types of auxiliary β subunits (KChIPs and DPPs, see Figure S1 and Video S1).^21^^,^^23^^,^^24^ Since KChIP and DPP interaction sites on Kv4 α-subunits do not overlap, effects of both β subunits on Kv4 channels are expected to be more or less additive.^25^ These β-subunit-mediated adaptations of channel physiology need to be considered, and the ability to interact with the β subunits needs to be tested when Kv4 channel function is to be evaluated, and a rigorous testing of putative variant pathogenicity is contemplated.

Video S1. Structure of the ternary Kv4/KChIP/DPP channel complex The three-dimensional structure of the Kv4.2/KChIP1/DPP6 complex reported by Kise and co-workers (PDB ID 7E8H)^21^ is used to display the ternary channel configuration (α-subunits: gray; KChIPs: orange; DPPs: blue) and the location of *KCND1* variant-associated amino acid substitutions. Substituted amino acid side chains are depicted as red (group 1 variants) and purple (group 2 variants) CPK models (for more information, see Figure S1)

Variants in several Kv channel genes are associated with monogenic types of epilepsy or developmental and epileptic encephalopathy.^26^ In particular, a number of heterozygous missense variants in *KCND2* (MIM: 605410) that alter Kv4.2 channel gating have been linked to early-onset global developmental delay of distinct severity, often in combination with seizures, muscular hypotonia, and/or visual impairment.^8^^,^^9^^,^^10^^,^^27^^,^^28^ Also, a hemizygous nonsense variant of X-chromosomal *KCND1* (MIM: 300281) has been suggested as a candidate pathogenic variant in a single individual with focal epilepsy, however, with no reference to Kv4.1 channel function.^29^

Here, we report on a cohort of 18 males with 17 distinct hemizygous *KCND1* variants, including two *de novo* missense variants (DNVs), three maternally inherited protein-truncating variants (PTVs), and 12 maternally inherited missense variants. Our genetic and functional analyses support the etiological involvement of Kv4.1 channel dysfunction in an X-linked neurodevelopmental disorder frequently associated with a variable neuropsychiatric clinical phenotype.

## Material and methods

### Recruitment of probands and genetic analysis

Utilizing GeneMatcher,^30^ 18 individuals from 17 families were gathered as part of an international collaborative project. In most cases, affected probands were initially examined by their referring physicians, genetic analyses were performed in a diagnostic setting, and genetic findings were subsequently used for secondary research. In all probands, *KCND1* variants were independently identified at the referring medical centers as the most-likely disease-causing variant. Biological samples were obtained after written informed consent was given by the affected individuals or their legal guardians. Informed consent for the publication of clinical and genetic data was obtained from all participants. The study was performed in accordance with the Declaration of Helsinki protocols and approved by the ethics committees of the respective institutions. Trio whole-exome sequencing (WES) was performed in all subjects, and identified *KCND1* variants were confirmed by Sanger sequencing. The different medical centers involved in this study utilized comparable protocols for DNA isolation, WES, bioinformatic processing, and variant interpretation. Functional impact of identified putative pathogenic variants was predicted via the MetaDome webserver,^31^ the AlphaMissense tool (https://alphamissense.hegelab.org/), the Rare Exome Variant Ensemble Learner (REVEL) algorithm (https://genome.ucsc.edu/cgi-bin/hgTrackUi?db=hg19&g=revel), the Combined Annotation Dependent Depletion (CADD) tool,^32^ and the Polymorphism Phenotyping v2 (PolyPhen-2) tool.^33^

### Plasmids and synthesis of Kv4.1 channels in *Xenopus* oocytes

The human *KCND1* cDNA (GenBank: NM_004979.6) encoding full-length Kv4.1 (GenBank: NP_004970.3, UniProtKB Q9NSA2)^34^ was inserted into pGEM-HE to generate pGEM-OK-hKCND1, which carries an optimized Kozak sequence. Variant-specific mutations were introduced utilizing the Quick-Change II site-directed mutagenesis kit (Agilent), and resulting constructs were verified by Sanger sequencing. Similar to a previous study, pGEM vectors encoding human KChIP2b^15^ and DPP6s (a generous gift from Nicole Schmitt, University of Copenhagen), two well-characterized isoforms present in many brain regions, and hereafter referred to as KChIP and DPP, respectively, were used for auxiliary β-subunit synthesis.^10^^,^^35^ Plasmid linearization, *in vitro* transcription, and cRNA injection in *Xenopus laevis* oocytes were done as previously described.^10^ All experimental procedures were in accordance with the national guidelines for the care and use of research animals and were approved by the local authorities. To generate Kv4.1 homotetramers, 5 ng total cRNA were injected per oocyte. Kv4.1 wild type (WT) and variant cRNAs were also co-injected KChIP or DPP cRNAs (5 + 5 ng per oocyte) for binary complex formation (only one type of auxiliary β-subunit) or with both KChIP and DPP cRNAs (2 + 5 + 5 ng per oocyte) for ternary complex formation.

### Electrophysiology

Oocytes were used for electrophysiological recordings 1–3 days after cRNA injection. Currents were recorded at room temperature (20°C–22°C) under two-electrode voltage clamp as described^10^ in a low chloride (15 mM) bath solution containing (in mM) 7.4 NaCl, 88.6 Na-aspartate, 2 KCl, 1.8 CaCl_2_, 1 MgCl_2_, and 5 HEPES, pH 7.4 with NaOH. Pulse protocols are explained in the figure legends. Data were analyzed using FitMaster (HEKA) and Kaleidagraph (Synergy Software), as described previously.^10^^,^^11^^,^^36^ Current decay kinetics were approximated with a double-exponential function and the kinetics of recovery from inactivation with a single-exponential function. The voltage dependences of activation and steady-state inactivation were analyzed with appropriate Boltzmann functions.^10^^,^^11^^,^^36^ Pooled data are presented as mean ± SD, unless stated otherwise. Statistical analyses for multiple groups are based on ANOVA with Dunnett’s post hoc testing and for two groups on Student’s t tests.

## Results

### Identification of hemizygous *KCND1* variants associated with variable neurological phenotypes

Utilizing trio WES and GeneMatcher, we identified distinct hemizygous coding variants of *KCND1* in a cohort of 18 male subjects from 17 families. The variant group includes one frameshift, two nonsense, and 12 missense mutations, all of which are maternally inherited, as well as two DNVs (Table 1). Affected individuals display diverse neurological abnormalities, primarily including delays in various developmental domains, distinct neuropsychiatric signs, and seizures (Tables 2 and S1). In each case, trio WES data analysis revealed the respective hemizygous *KCND1* variant as the most-likely genetic cause of the observed clinical phenotype, as no other (likely) pathogenic variants that could explain the observed clinical features were identified. *KCND1* is located on the X chromosome and encodes the Kv4.1 Kv channel α subunit.^34^ Although Kv4 channels play a crucial role in brain function,^2^ variants in *KCND1* have not been associated with a human disease phenotype in OMIM, though a single individual with focal epilepsy has been reported as hemizygous for a truncating variant in this gene.^29^ Thus, the two central aims of the present work were to (1) functionally characterize the identified *KCND1* variants, thereby allowing their classification beyond uncertain clinical significance and (2) provide an overview of the phenotypic spectrum associated with hemizygous *KCND1* variants.

**Table 1:** Description and *in silico* analysis of *KCND1* variants

**Genetic analysis**											**Bioinformatic analysis**				
**Individual**	**Transcript GenBank: NM_004979.6**	**Protein GenBank:****NP_004970.3**	**gnomAD v2.1.1**				**gnomAD v4.0.0**				**MetaDome**	**REVEL**	**CADD**	**Alpha-Missense**	**Poly-Phen-2**
			**Allele** **count**	**# of hemi-zygotes**	**Allele** **frequency**	**Allele** **frequency in hemizygotes**	**Allele** **count**	**# of hemi-zygotes**	**Allele** **frequency**	**Allele** **frequency in hemizygotes**					
***De novo* missense variants**															

1	c.274C>T	p.Arg92Cys^a^	0	0	0	0	11	3	9.09e-6	7.54e-6	1.31	0.404	24.7	0	1.0
2	c.343G>A	p.Asp115Asn^a^	1	0	5.58e-6	0	18	5	1.49e-5	1.26e-5	0.53	0.165	23.6	0.037	0.388

**Maternally inherited truncating variants**															

3	c.182_194del	p.Tyr61Cysfs^∗^31^a^	0	0	0	0	0	0	0	0	N/A	N/A	N/A	N/A	N/A
4	c.295C>T	p.Arg99^∗^^a^	2	0	1.1e-5	0	6	2	4.96e-6	5.03e-6	N/A	N/A	35	N/A	N/A
5	c.1348A>T	p.Lys450^∗^^a^	1	0	5.51e-6	0	5	1	8.87e-6	4.99e-6	N/A	N/A	37	N/A	N/A

**Maternally inherited missense variants**															

**Variants affecting the N-terminal cytoplasmic domain**															

6	c.170C>T	p.Thr57Met^a^	3	0	1.67e-5	0	12	0	9.92e-6	0	0.14	0.858	25.3	0.909	0.986
7	c.178C>T	p.Arg60Cys^a^	0	0	0	0	2	0	1.82e-6	0	0.16	0.469	29.0	0.534	1.0
8	c.238G>C	p.Gly80Arg	0	0	0	0	0	0	0	0	0.36	0.101	18.14	0.145	0.001
9	c.320G>A	p.Arg107Gln^a^	0	0	0	0	6	2	4.96e-6	5.03e-6	0.55	0.395	23.1	0.225	0.956
10	c.436C>T	p.Arg146Cys^a^	1	0	5.71e-6	0	3	2	2.74e-6	8.78e-6	0.18	0.807	26.5	0.952	1.0
11	c.442G>A	p.Ala148Thr	0	0	0	0	1	0	2.2e-6	0	0.23	0.438	20.7	0.112	0.471

**Variants affecting transmembrane domains**															

12	c.604G>A	p.Ala202Thr	5	2	2.53e-5	2.83e-5	130	35	1.08e-4	8.82e-5	0.74	0.613	22.7	0.207	0.236
13	c.922C>T	p.His308Tyr^a^	0	0	0	0	0	0	0	0	0.69	0.774	25.3	0.970	0.786

**Variants affecting the C-terminal cytoplasmic domain**															

14	c.1291C>T	p.Arg431Cys^a^	2	1	1.11e-5	1.56e-5	5	3	4.58e-6	8.38e-6	1.79	0.694	33	0.936	1.0
15/16	c.1546A>T	p.Thr516Ser	11	3	5.81e-5	4.63e-5	277	79	2.3e-4	2.01e-4	0.9	0.157	15.87	0.102	0.044
17	c.1606A>G	p.Arg536Gly^a^	3	2	1.68e-5	3.11e-5	32	14	2.65e-5	3.52e-5	0.46	0.822	25.1	0.194	0.949
18	c.1733A>T	p.Asn578Ile^a^	0	0	0	0	0	0	0	0	0.55	0.814	26.9	0.756	1.0

Individuals 15 and 16 are brothers of different age. Allele count, number of identified hemizygotes, and allele frequency of individual variants are derived from the gnomAD database (https://gnomad.broadinstitute.org/, gnomAD v2.1.1, GRCh37/hg19 and gnomAD v4.0.0, GRCh38/hg38; as of January 22, 2024). MetaDome tolerance scores are calculated as described (https://stuart.radboudumc.nl/metadome/). The phred-like scaled C score predicted by CADD (https://wintervar.wglab.org/) ranges from 1 to 99 with higher values indicating more deleterious variants. The AlphaMissense score (https://alphamissense.hegelab.org/) ranges from 0.0 to 1.0 with scores below 0.34 considered likely benign and scores higher than 0.564 being considered likely pathogenic. The REVEL and the PolyPhen-2 scores range from 0.0 (tolerated) to 1.0 (deleterious). CADD, Combined Annotation Dependent Depletion; HGVS, Human Genome Variation Society; N/A, not applicable.

a Respective native amino acids are conserved throughout the Kv4 family.

**Table 2:** Clinical features of subjects with hemizygous *de novo* missense or maternally inherited truncating *KCND1* group 1 variants

**Individual #**	**1**	**2**	**3**	**4**	**5**
cDNA variant GenBank: NM_004979.6	c.274C>T	c.343G>A	c.182_194del	c.295C>T	c.1348A>T
Protein variant GenBank: NP_004970.3	p.Arg92Cys	p.Asp115Asn	p.Tyr61Cysfs^∗^31	p.Arg99^∗^	p.Lys450^∗^
Variant origin	*de novo*	*de novo*	maternally inherited	maternally inherited	maternally inherited
Age at last examination	15 years	9 years 11 months	16 years	16 years	3 years 2 months
Age at first signs	10 years	6 years	2 years 3 months	abt 2 years	neonatal period
First signs	anxiety, tics	speech delay, seizures	seizures	speech delay, motor delay, anxiety	speech delay, motor delay
Motor development	normal	normal	normal	delayed	delayed
Intellectual development	average	low average, TIQ 85	average	borderline mental functioning – low average, TIQ 74-86	extremely low/impaired, TIQ 68
Speech development	normal	delayed	normal	delayed	delayed
Receptive language	normal	below average	normal	below average	below average
Verbal ability	normal	pronunciation problems, grammar problems	normal	pronunciation problems at younger age	pronunciation problems, uses only very short sentences
Comprehension	normal	below average	normal	below average	below average
Neuropsychiatric signs	ASD, attention deficit, anxiety, tics	attention deficit	none	ASD, anxiety, emotional problems	attention deficit
Seizures/epilepsy	no	epilepsy, absences, myoclonus, eyelid myoclonia	epilepsy, generalized tonic-clonic seizures during sleep until 11 years, then seizures remitted, Valproate responsive	no	no
Electro-encephalography	normal	irregular polyspikes and spike-waves, photoparoxysmal reaction	generalized abnormalities at 3 years 8 months, normal at 12 years 6 months	ND	ND

abt, about; ASD, autism spectrum disorder; ND, no data; TIQ, total intelligence quotient.

Initially, we focused on a set of five variants (group 1) for which the genetic data provide the strongest evidence for pathogenicity, i.e., DNVs and PTVs. The allele frequency in hemizygotes in the gnomAD database (v4.0.0, GRCh38/hg38)^37^ is very low for the DNV c.274C>T (p.Arg92Cys) (7.54e-6, individual 1) and low for the DNV c.343G>A (p.Asp115Asn) (1.26e-5, individual 2) (Table 1). Both affected amino acid residues in the T1-domain are completely conserved throughout the Kv4 subfamily (Figure 1A). While well-established prediction tools support the pathogenicity of p.Arg92Cys, they provide ambiguous results for p.Asp115Asn (Table 1). We also identified three PTVs, namely c.182_194del (p.Tyr61Cysfs^∗^31) (individual 3), c.295C>T (p.Arg99^∗^) (individual 4), and c.1348A>T (p.Lys450^∗^) (individual 5). While the former is not listed in gnomAD, p.Arg99^∗^ and p.Lys450^∗^ are both found at very low allele frequencies in hemizygotes (around 5.0e-6). High-scaled C-scores predicted by CADD suggest that the latter two are pathogenic variants. All PTVs are predicted to lead to nonsense-mediated decay (NMD) of the respective transcripts, thus resulting in a complete loss-of-function (LOF) in hemizygous males. However, as NMD is known to exhibit a large variability in its efficiency across mRNAs, cell types, tissues, and individuals, we also considered the possibility of NMD escape.^38^ In case of NMD escape and synthesis of p.Tyr61Cysfs^∗^31 and p.Arg99^∗^, both truncated proteins are expected to remain cytosolic and be unable to form functional channels due to the lack of any transmembrane domains (Figure 1), thus still leading to complete LOF. In contrast, if synthesized, p.Lys450^∗^ contains transmembrane regions S1–S6 (Figure 1) and is hence theoretically able to form functional channels. Thus, from the five group 1 variants, p.Arg92Cys, p.Asp115Asn, and p.Lys450^∗^ were subsequently chosen for functional characterization, while cytosolic p.Tyr61Cysfs^∗^31 and p.Arg99^∗^, although patho-physiologically relevant, were not included in these experiments.

### *KCND1* group 1 variants: Functional characterization and associated clinical features

**Figure 2:**
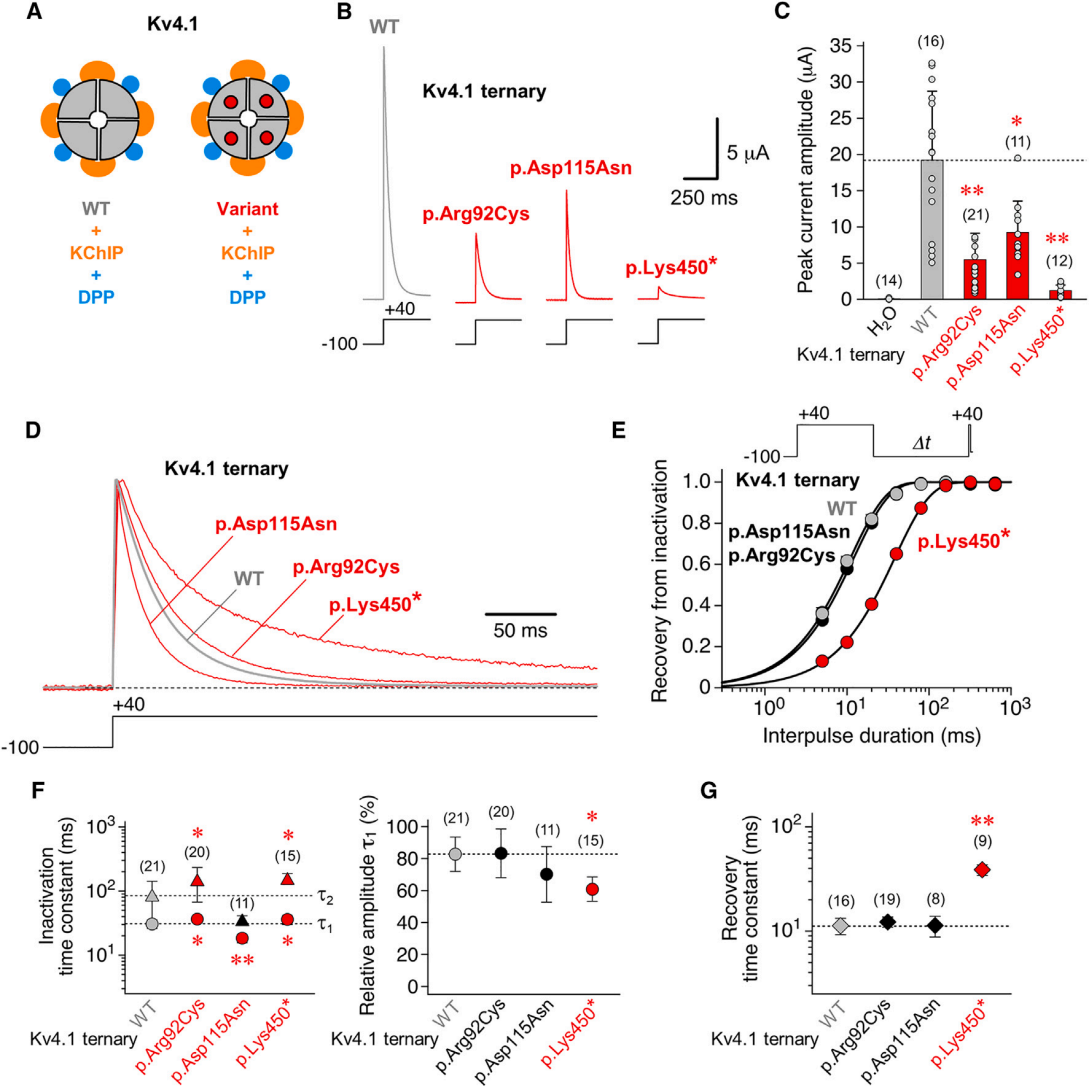
Functional expression and kinetic analysis of inactivation for *KCND1* WT and group 1 variants in a ternary configuration (A) Kv4.1 wild-type channels (WT, gray quadrants) and variant channels with amino acid substitutions (quadrants with red dots) were functionally analyzed in a ternary configuration, i.e., with both KChIP (orange) and DPP (blue, see also Figure S1 and Video S1) in *Xenopus laevis* oocytes (see material and methods). (B) Macroscopic currents mediated by Kv4.1 WT, p.Arg92Cys, p.Asp115Asn, and p.Lys450^∗^ ternary channels. Currents were recorded under two-electrode voltage clamp using depolarizing voltage steps from −100 to +40 mV, as indicated (gray trace: WT; red traces: p.Arg92Cys, p.Asp115Asn, and p.Lys450^∗^). (C) Bar graph shows mean peak current amplitudes, including individual data points (number of observations indicated). Note that for all functionally tested group 1 variants (red bars) mean peak current amplitudes were smaller than for Kv4.1 WT (gray bar and horizontal dotted line). Water-injected oocytes served as control. (D) Normalized currents mediated by Kv4.1 WT (gray trace), p.Arg92Cys, p.Asp115Asn, and p.Lys450^∗^ (red traces) in a ternary configuration. Currents were elicited by extended voltage pulses, as indicated (2.5 s, +40 mV), to study the kinetics of macroscopic inactivation (i.e., current decay). (E) Recovery of ternary channels from inactivation was measured using a double-pulse protocol with long control and brief test pulses to +40 mV and variable interpulse durations at −100 mV (*Δt*, see inset). The normalized data (I_test_/I_control_) were plotted against the interpulse duration and described by a single-exponential function (gray symbols: Kv4.1 WT; black symbols: p.Arg92Cys and p.Asp115Asn; red symbols: p.Lys450^∗^; error bars are SEM and smaller than symbols). (F) Inactivation time constants obtained by fitting the current decay kinetics with a double-exponential function (τ_1_: circles, τ_2_: triangles) and the relative amplitude of the total decay accounted for by τ_1_. (G) Recovery time constants obtained by fitting the kinetics of recovery from inactivation with a single-exponential function. (F and G) Gray symbols and horizontal dotted lines: WT; black symbols: values obtained for variant ternary channels with no difference compared to Kv4.1 WT; red symbols: values obtained for variant ternary channels that significantly differ from Kv4.1 WT; number of observations indicated; all statistics based on one way ANOVA with Dunnett’s post-hoc testing; significant differences compared to Kv4.1 WT are indicated with ^∗^*p <* 0.05 or ^∗∗^*p <* 0.0001.

First of all, Kv4.1 WT and variant channels were functionally characterized in a ternary configuration, i.e., in the presence of both types of auxiliary β subunits (KChIP and DPP, Figures 2A and S1 and Video S1), a likely channel configuration in native neuronal tissues^24^ (see material and methods). For the ternary Kv4.1 channel complexes, the following biophysical parameters were examined under two-electrode voltage clamp in *Xenopus* oocytes (see material and methods): peak current amplitude, macroscopic inactivation kinetics (i.e., kinetics of current decay), kinetics of recovery from inactivation, and voltage dependences of activation and steady-state inactivation.

**Figure 3:**
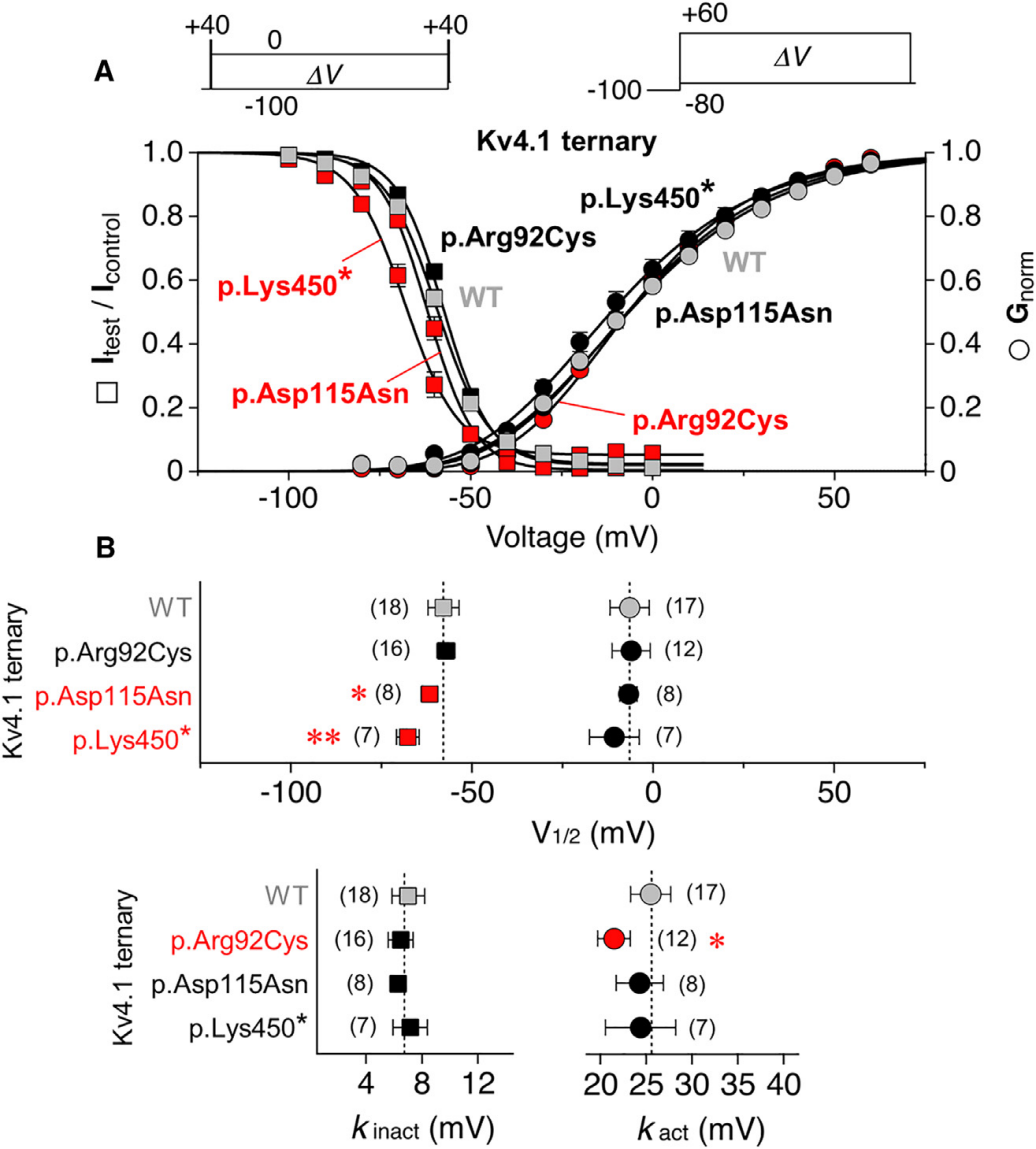
Voltage dependence of activation and steady-state inactivation (A) Voltage dependences of peak conductance activation (circles) and steady-state inactivation (squares) are shown for Kv4.1 wild-type (WT), p.Arg92Cys, p.Asp115Asn, and p.Lys450^∗^ ternary channels. For the study of steady-state inactivation, brief control and test pulses to +40 mV were separated by a 10 s conditioning pulse (*ΔV* between −100 and 0 mV in 10 mV increments, see inset). Normalized data (I_test_/I_control_) were plotted against the conditioning pulse voltage. For the study of peak conductance activation, test pulses to voltages between −80 and +60 mV (*ΔV* in 10 mV increments) were applied from −100 mV (see inset). Normalized conductance values were plotted against the test pulse voltage. The data were fitted with appropriate Boltzmann functions.^11^^,^^36^ Gray symbols: Kv4.1 WT; black symbols: data which do not differ from Kv4.1 WT; red symbols: data that differ from Kv4.1 WT (error bars are SEM). (B) Voltages of half-maximal inactivation (squares) and half-maximal activation (circles) and corresponding slope factors (*k*_inact_ and *k*_act_, respectively); gray symbols and vertical dotted lines: Kv4.1 WT; black symbols: V_1/2_ and *k* values of variant ternary channels with no difference compared to Kv4.1 WT; red symbols: V_1/2_ and *k* values of variant ternary channels that significantly differ from Kv4.1 WT; number of observations indicated; all statistics based on one way ANOVA with Dunnett’s post-hoc testing; significant differences compared to Kv4.1 WT are indicated with ^∗^*p <* 0.05 or ^∗∗^*p <* 0.0001.

Both Kv4.1 WT and variant ternary channels, even the truncated protein p.Lys450^∗^, mediated rapidly, activating and inactivating currents with variable amplitudes and decay kinetics in response to depolarizing voltage steps (Figure 2B). Mean peak current amplitudes at a test voltage of +40 mV (19.2 ± 9.52 μA for Kv4.1 WT, *n* = 16) were found to be reduced for all three variants (Figure 2C). Macroscopic inactivation kinetics (τ_1_ = 30.7 ± 4.72 ms, relative amplitude = 83 ± 11% and τ_2_ = 85.8 ± 56.8 ms for Kv4.1 WT, *n* = 21) were accelerated for p.Asp115Asn, slowed for p.Arg92Cys and p.Lys450^∗^, and the relative amplitude accounted for by τ_1_ was significantly decreased for p.Lys450^∗^ (Figures 2D and 2F). The kinetics of recovery from inactivation (τ_rec_ = 11.2 ± 2.02 ms for Kv4.1 WT, *n* = 16), were unaffected for p.Arg92Cys and p.Asp115Asn but slowed for p.Lys450^∗^ (Figures 2E and 2G). Finally, we examined the voltage dependences of activation and steady-state inactivation (Figure 3A). For Kv4.1 WT, Boltzmann analysis yielded voltages of −6.45 ± 5.42 mV (*n =* 17) and −57.9 ± 4.30 mV (*n =* 18) for half-maximal activation and inactivation, respectively. Except for a decreased slope factor (steepening) of the p.Arg92Cys activation curve, the voltage dependence of activation remained unaltered for all variants. The voltage dependence of steady-state inactivation was similar to Kv4.1 WT for p.Arg92Cys but negatively shifted for p.Asp115Asn and p.Lys450^∗^ with no significant effects on the steepness of inactivation curves (Figure 3B, results summarized in Table S3). To summarize, functional analyses of group 1 variants p.Arg92Cys, p.Asp115Asn, and p.Lys450^∗^ in a ternary channel configuration revealed that (1) all three variants significantly differ from WT, and (2) each of them displays distinct variant-specific alterations of biophysical channel properties, thus providing evidence for their pathogenicity. Since the two remaining group 1 variants, p.Tyr61Cysfs^∗^31 and p.Arg99^∗^, which were not functionally studied, are predicted to result in complete LOF, either due to NMD of the respective mRNA or the inability of the encoded protein to form functional channels, the above data collectively suggest that all five identified group 1 *KCND1* variants are pathogenic, impairing Kv4.1 channel function and resulting in an X-linked neurodevelopmental disorder with variable expressivity.

From a clinical perspective, group 1 *KCND1* variants are associated with a neurodevelopmental disorder variable in severity, with or without seizures (Table 2). First clinical signs were noted between the neonatal period and 10 years of age. Four individuals exhibit neuropsychiatric signs, including autism spectrum disorder (ASD), attention deficit disorder, anxiety, tics, and emotional problems. In addition, three of these subjects present with speech delay, receptive language and comprehension problems, and impaired intellectual development. Two individuals have different types of epilepsy, while two others present with delayed motor development. There is no apparent genotype-phenotype correlation.

### Maternally inherited *KCND1* missense variants (group 2)

In addition to the five group 1 variants described above, we identified 13 male individuals carrying 12 distinct maternally inherited *KCND1* missense variants (group 2; Table 1), which were initially considered variants of unknown significance (VUSs). Eleven group 2 variants are private in unrelated males, while p.Thr516Ser was identified in two brothers of different age (individuals 15 and 16; Table 1). The phenotypic spectrum associated with group 2 variants overlaps with the range of phenotypes exhibited by the individuals bearing group 1 variants (Table S1). All 18 individuals present with neurological, albeit rather variable, abnormalities. Frequent clinical signs include impaired intellectual development (14 individuals), delayed speech development (14 individuals), impaired comprehension (13 individuals), neuropsychiatric signs (11 individuals), impaired receptive language (nine individuals), delayed motor development (eight individuals), muscular hypotonia (seven individuals), and different forms of epilepsy (six individuals). Neuropsychiatric features include ASD, attention deficit, hyperactivity, anxiety, tics, emotional problems, and poor social skills. Of note, the two differently aged brothers carrying the c.1546A>T (p.Thr516Ser) variant (individuals 15 and 16) share a common phenotype with only marginal differences. Both boys present with speech delay, reduced receptive language and comprehension, delayed intellectual development, and abnormalities in cerebral magnetic resonance imaging, while until now only the younger brother has been diagnosed with ASD. Thus, despite a rather large clinical variability within the entire cohort, a particular variant may be associated with a more defined phenotypic pattern. It is also worth noting that the variant p.His308Tyr is the one that deviates the most from Kv4.1 WT in terms of its biophysical parameters (see below). However, since the affected child was only two years old at the time of the most recent medical examination, it is not yet possible to definitively state whether these drastic functional changes correlate with a particularly severe phenotype (individual 13). Subject 14 deceased at the age of eight months due to respiratory insufficiency, probably linked to progressive hypomyelinating encephalopathy. Reportedly, in all cases, the heterozygous carrier mothers are clinically unaffected.

Within group 2, the pathogenicity of six variants is strongly supported by the fact that they are not listed as hemizygous variants in gnomAD (p.Thr57Met, p.Arg60Cys, p.Gly80Arg, p.Ala148Thr, p.His308Tyr, and p.Asn578Ile). The combined data from four distinct pathogenicity prediction algorithms indicate that six variants (p.Thr57Met, p.Arg146Cys, p.His308Tyr, p.Arg431Cys, p.Arg536Gly, and p.Asn578Ile) are likely pathogenic, while the prediction for the other six variants is rather ambiguous.

On the protein level, nine of the 12 substituted amino acid residues are conserved throughout the Kv4 subfamily (Figure 1A), thus suggesting potential biological relevance. Variant-specific amino acid exchanges primarily reside in the cytoplasmic N- or C-termini of Kv4.1, with only two exceptions affecting S1 or S4 (Figures 1 and S1 and Video S1). While these findings appear to suggest that both intracellular termini of Kv4.1 represent hotspots for disease-associated amino acid substitutions, the exact pathogenic potential of the respective variants in a hemizygous state remains unclear. Thus, after having ensured based on immunocytochemical experiments with epitope-tagged constructs that all variants are synthesized and integrated in the plasma membrane of transfected human cells (data not shown), we aimed at their functional characterization to aid clinical variant classification.

**Figure 4:**
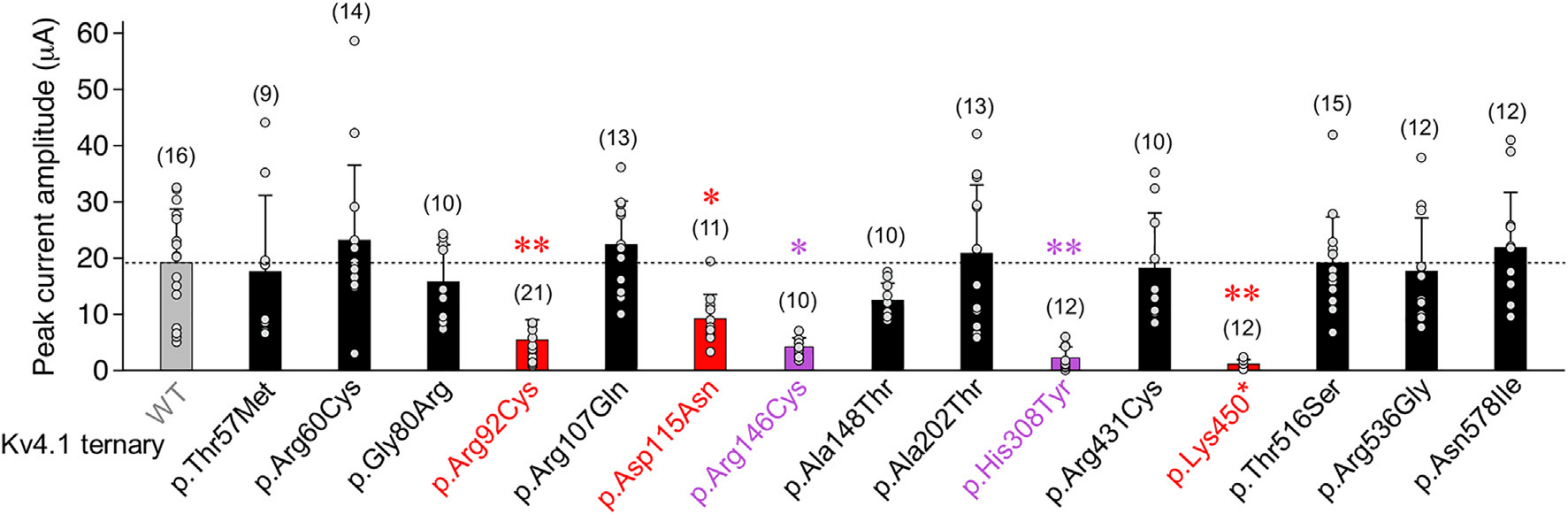
Functional expression of group 2 variants in a ternary configuration Mean peak current amplitudes at +40 mV including individual data points (number of observations indicated; gray bar and horizontal dotted line: Kv4.1 WT data from Figure 2C; red bars: variant ternary channel data from Figure 2C; black bars: Peak amplitude values for variant ternary channels with no difference compared to Kv4.1 WT; purple bars: peak amplitude values of variant ternary channels that significantly differ from Kv4.1 WT; all statistics based on one way ANOVA with Dunnett’s post-hoc testing; significant differences compared to Kv4.1 WT are indicated with ^∗^*p <* 0.05 or ^∗∗^*p <* 0.0001.

**Figure 5:**
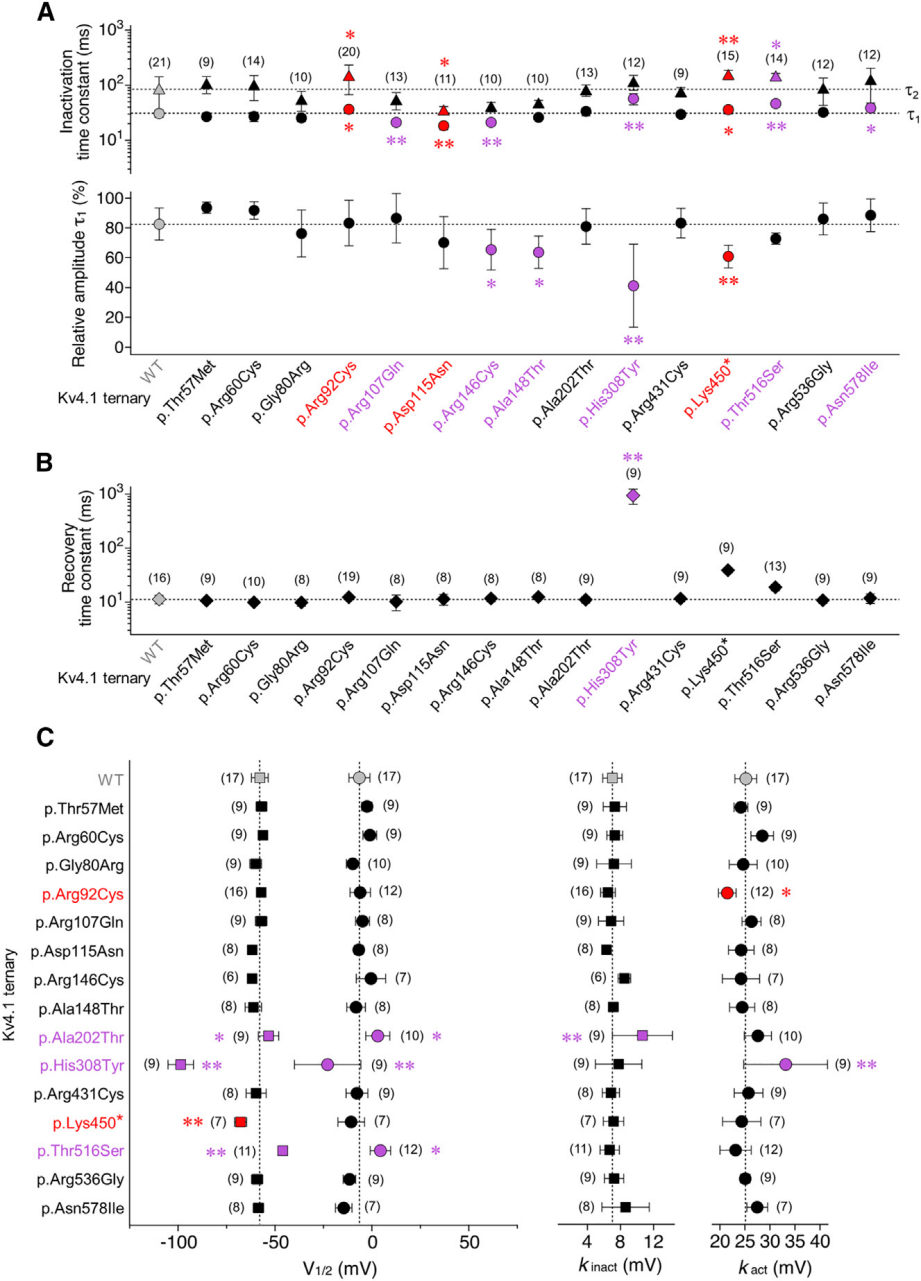
Biophysical characterization of group 2 variants in a ternary configuration (A) Inactivation time constants obtained by fitting the Kv4.1-mediated current decay kinetics with a double-exponential function (τ_1_: circles, τ_2_: triangles) and the relative amplitude of the total decay accounted for by τ_1_ (number of observations indicated). (B) Recovery time constants obtained by fitting the kinetics of recovery from inactivation with a single-exponential function (number of observations indicated). (C) Voltages of half-maximal inactivation (squares) and half-maximal activation (circles) and corresponding slope factors (*k*_inact_ and *k*_act_, respectively; number of observations indicated). (A–C) Gray symbols and dotted lines: Kv4.1 WT data from Figures 2F, 2G, and 3B; red symbols: variant ternary channel data from Figures 2F, 2G, and 3B that significantly differ from Kv4.1 WT when testing 15 variants, including p.His308Tyr; black symbols: values obtained for variant ternary channels with no difference compared to Kv4.1 WT (including part of the p.Asp115Asn and p.Lys450^∗^ data); purple symbols: values obtained for variant ternary channels (maternally inherited missense variants) that significantly differ from Kv4.1 WT; number of observations indicated; all statistics based on one way ANOVA with Dunnett’s post-hoc testing; significant differences compared to Kv4.1 WT are indicated with ^∗^*p <* 0.05 or ^∗∗^*p <* 0.0001.

When tested in a ternary configuration (i.e., in the presence of both KChIP and DPP), all 12 maternally inherited missense variants mediated A-type currents and thus allowed further functional characterization. Of note, all group 2 variants as well as the three previously studied group 1 variants were included in the statistical analysis of the functional data. Reduced peak current amplitudes compared to Kv4.1 WT were only seen for p.Arg146Cys and p.His308Tyr (Figure 4). However, macroscopic inactivation kinetics were differentially altered for six out of the 12 variants, with an overall acceleration for p.Arg107Gln and p.Arg146Cys and an overall slowing for p.His308Tyr, p.Thr516Ser, and p.Asn578Ile, while in p.Ala148Thr only the relative amplitude of τ_1_ was reduced in comparison to Kv4.1 WT (Figure 5A). Notably, in none of the 12 variants, the recovery from inactivation was affected, except for p.His308Tyr, which showed a remarkable about 100-fold slowing (Figure 5B). The voltage dependences of activation and/or steady-state inactivation were modified for three of the 12 variants. Thus, for p.His308Tyr, both voltage dependences were negatively shifted, whereas for p.Ala202Thr and p.Thr516Ser, both voltage dependences were positively shifted. In addition, increased slope factors (flattening) were obtained for the p.Ala202Thr inactivation curve and the p.His308Tyr activation curve (Figure 5C, results summarized in Table S3). Taken together, when tested in a ternary configuration, significant variant effects on one or more electrophysiological parameters were observed for seven out of 12 group 2 variants. It should be noted that, due to the inclusion of a large number of variants in addition to the initially studied group 1 variants, and particularly due to the drastic effects caused by p.His308Tyr, ANOVA might underestimate the significance of effects associated with some of the variants.

### Variant-associated effects in Kv4.1 homotetramers and binary channel complexes

In the absence of a precise knowledge of the availability of KChIP and DPP subunits at early developmental stages, in specific brain regions, cell types, or subcellular compartments, further electrophysiological experiments were performed with Kv4.1 homotetramers and binary channel complexes (containing only one of the two auxiliary β subunits). First, Kv4.1 WT and variant channels were examined in the absence of auxiliary β subunits to find out whether a variant exerts an intrinsic effect on biophysical properties. Second, channels were synthesized together with either KChIP or DPP to assess possible dysfunctions of binary channel complexes (results summarized in Tables S4–S6). Our experiments revealed for instance that, although inconspicuous in the ternary configuration, p.Gly80Arg, p.Ala202Thr, p.Thr516Ser, and p.Asn578Ile show an increase, whereas p.Arg431Cys shows a decrease in peak current amplitude in the absence of auxiliary β subunits. Similarly, macroscopic inactivation kinetics were accelerated for p.Thr57Met and slowed for p.Gly80Arg and p.Arg536Gly, and concerning the voltage dependences, positive shifts were seen for p.Thr57Met and p.Arg92Cys, and negative shifts were seen for p.Asp115Asn, p.Ala148Thr, p.Arg431Cys, and p.Asn578Ile in the absence of auxiliary β subunits but not in the ternary configuration. For other variants, intrinsic functional alterations correspond to those observed in the ternary configuration. Thus, peak current amplitudes were intrinsically lowered for p.Arg92Cys, p.His308Tyr, and p.Lys450^∗^; macroscopic inactivation kinetics were intrinsically slowed for p.Lys450^∗^ and p.Asn578Ile; and recovery kinetics were intrinsically slowed for p.His308Tyr. Finally, the voltage dependence of steady-state inactivation was intrinsically shifted positive for p.Ala202Thr and p.Thr516Ser, and both voltage dependences were intrinsically shifted negative for p.His308Tyr. Taken together, our findings show that variant-specific alterations of individual biophysical parameters are often apparent in all four tested channel configurations. On the other hand, the only functional alteration found for p.Arg60Cys was a modest acceleration of macroscopic inactivation kinetics, reflected by an increase in the relative amplitude of τ_1_ in the DPP binary complex.

Of note, the presence of only one β subunit (binary channels) also yielded valuable information on whether a variant channel was still able to functionally interact with KChIP or DPP. As seen for Kv4.1 WT and the majority of variants, the concurrent presence of KChIP slowed the initial phase and accelerated the late phase of macroscopic inactivation, accelerated the recovery from inactivation, and positively shifted the voltage dependence of steady-state inactivation, whereas the presence of DPP accelerated both the initial phase of macroscopic inactivation and recovery from inactivation and negatively shifted the voltage dependences of both activation and steady-state inactivation. These typical β-subunit effects on Kv4.1 channel function were lost for certain variants (see Figures S3–S5) thus representing a further putative pathophysiological mechanism.

### Classification of *KCND1* group 2 variants

Finally, an assessment of the pathogenicity probability of group 2 variants should be made, taking into account the above findings. Thus, we relied on the well-established American College of Medical Genetics and Genomics (ACMG) guidelines for clinical variant interpretation.^39^^,^^40^ This classification system utilizes the evidence code PS3 based on the results of “well-established” functional assays, which are suitable to discriminate between abnormal and normal gene/protein function. In the past, the highly reproducible and robust two-electrode voltage-clamp technique in *Xenopus* oocytes has been used successfully to assess the biophysical features of ion channels, including variants of *KCND2*/Kv4.2^8^^,^^9^^,^^10^^,^^41^^,^^42^ and *KCND3*/Kv4.3,^43^^,^^44^^,^^45^ as well as other voltage-gated potassium channels.^46^ Therefore, we performed PS3 scoring of *KCND1* variants on the basis of the results from this functional assay system. In particular, to rate functional impairment of Kv4.1 variants, we included the five biophysical parameters examined (peak current amplitude, macroscopic inactivation, recovery from inactivation, and the voltage dependences of activation and steady-state inactivation) from all analyzed channel configurations (ternary complexes, Kv4.1 alone, and both binary channel compositions). In addition, we incorporated the functional interaction of Kv4.1 variants with KChIP2 and/or DPP6. Thus, to assess physiological impairment of a given functionally analyzed variant, 29 items were taken into account (Table S7). If four or more of these functional parameters are significantly altered compared to Kv4.1 WT, this is valued as supportive evidence for a pathogenic effect on channel function (ACMG criterion PS3, see discussion). This definition is based on the idea that variants showing normal interaction with auxiliary β subunits meet the requirements to pass the threshold if they differ from Kv4.1 WT in only one of the five biophysical parameters examined but do so consistently in all four channel configurations tested. All but two group 2 variants (p.Arg60Cys and p.Arg536Gly) exceed this threshold value (Table S7), with p.His308Tyr reaching the maximum number of 22 altered parameters, some of which are drastically affected. For comparison, 12 (p.Asp115Asn) and 15 (p.Arg92Cys and p.Lys450^∗^) items are significantly altered in the functionally analyzed pathogenic group 1 variants. According to ACMG classification, three group 2 variants are considered to be likely pathogenic, whereas the nine remaining variants are categorized as VUSs (Table 3).

**Table 3:** Classification of *KCND1* group 2 variants

**Individual**	**Transcript GenBank: NM_004979.6**	**protein GenBank:****NP_004970.3**	**Used ACMG criteria**	**ACMG classification**^a^
**Maternally inherited missense variants**				

**Variants affecting the N-terminal cytoplasmic domain**				

6	c.170C>T	p.Thr57Met	PS3, PM2_sup, PP3	likely pathogenic
7	c.178C>T	p.Arg60Cys	PM2_sup, PP3	VUS
8	c.238G>C	p.Gly80Arg	PS3, PM2_sup, BP4	VUS
9	c.320G>A	p.Arg107Gln	PS3	VUS
10	c.436C>T	p.Arg146Cys	PS3, PP3	VUS
11	c.442G>A	p.Ala148Thr	PS3, PM2_sup	VUS

**Variants affecting transmembrane domains**				

12	c.604G>A	p.Ala202Thr	PS3, BS2	VUS
13	c.922C>T	p.His308Tyr	PS3, PM2_supp, PP3	likely pathogenic

**Variants affecting the C-terminal cytoplasmic domain**				

14	c.1291C>T	p.Arg431Cys	PS3, PP3	VUS
15 and 16	c.1546A>T	p.Thr516Ser	PS3, BP4, BS2	VUS
17	c.1606A>G	p.Arg536Gly	PP3	VUS
18	c.1733A>T	p.Asn578Ile	PS3, PM2_sup, PP3	likely pathogenic

American College of Medical Genetics and Genomics (ACMG) criteria: PS3, well-established *in vitro* or *in vivo* functional studies supportive of a damaging effect on the gene or gene product; PM2, absent from controls (or at extremely low frequency if recessive) in Exome Sequencing Project, 1000 Genomes Project, or Exome Aggregation Consortium; PP3, multiple lines of computational evidence support a deleterious effect on the gene or gene product (conservation, evolutionary, splicing impact, etc.); BP4, multiple lines of computational evidence suggest no impact on gene or gene product (conservation, evolutionary, splicing impact, etc.); BS2, variant was observed in a hemizygous state in population databases more than expected for disease.

a To assign variant class, a points-based system to simplify scoring was used.^40^ See Table S7 for more details on PS3 scoring.

## Discussion

Here, we describe 17 distinct hemizygous coding variants of *KCND1*, which were independently identified as the most-likely disease-causing variant in 18 male subjects presenting with a variable neurodevelopmental phenotype. To determine variant pathogenicity, we employed genomic data analyses and comprehensive functional characterization of biophysical channel features. Our results provide strong supporting evidence that hemizygous *KCND1* variants that differentially alter Kv4.1 channel properties are involved in the pathogenesis of an X-linked neurodevelopmental condition, which is often associated with a variable neuropsychiatric phenotype.

### Clinical phenotypes

The *KCND1* variants described herein are associated with a neurodevelopmental disorder characterized by a remarkable variability in the phenotypic expression and severity of the disease. Although the clinically documented onset of the disease varies from neonatal to ten years of age, all but two individuals were younger than five years of age when the first clinical signs were diagnosed. In the two exceptions, it is not known whether an earlier clinical diagnosis would have been possible. The disease phenotype of the entire cohort ranges from rather complex and severe neurological phenotypes to more or less isolated clinical signs. Individual clinical features noticed in isolation were also observed as syndromic attributes. Prevalent clinical signs are intellectual disability, delayed speech development, hindered comprehension, neuropsychiatric signs, impaired receptive language, motor delay, muscular hypotonia, and different types of epilepsy. Neuropsychiatric abnormalities observed in about two-thirds of the subjects include ASD, attention deficit, hyperactivity, anxiety, tics, emotional problems, and poor social skills. There is no obvious genotype-phenotype correlation, e.g., similar genetic alterations or amino acid exchanges affecting identical parts of the channel protein do not appear to result in a common phenotype. Several *KCND1* variants, including two predicted LOF variants, are listed as rare hemizygous variants in gnomAD v4.0.0 (Table 1). This finding is consistent with a highly variable disease phenotype that includes adult males with a mild (or late-onset) or even no apparent clinical phenotype, thus potentially broadening the clinical variability described herein. Unfortunately, to our knowledge, there are no suitable published or otherwise available data on a *KCND1* LOF animal model to verify this hypothesis. However, in a single individual with childhood-onset focal epilepsy, a hemizygous *KCND1* nonsense variant was identified as the prime candidate for a pathogenic variant.^29^ Moreover, LOF hemizygotes are listed in gnomAD for several well-known X-linked neurodevelopmental genes, including genes that also encode transmembrane proteins with ion channel/transporter function, such as *GRIA3*^47^ (MIM: 305915) and *SLC9A6*^48^ (MIM: 300231) for both of which pathogenic LOF variants have been described. It should also be noted that the *KCND1* cohort presented in our study is likely to be biased to the more severe end of the phenotypic spectrum, as all subjects were selected based on distinctive clinical abnormalities exhibited during childhood. Taken together, it is tempting to speculate that hemizygous *KCND1* variants lead to a wide-ranging continuum of neurodevelopmental abnormalities reaching from clinically largely unremarkable phenotypes to early-onset, rather severe syndromic conditions. While the reasons for this large degree of phenotypic variability are currently unknown, it is important to note that distinct variants of a given gene are unlikely to act in isolation. Instead, other genomic variants may act as genetic modifiers to either alleviate or exacerbate disease severity, thus leading to a variable phenotypic outcome. In the future, it will be interesting to identify genetic modifiers of pathogenic *KCND1* variants and determine their mutual interaction. Attractive candidate modifiers are, e.g., genes encoding proteins that interact with Kv4.1, such as members of the KChIP and DPP families.

### ACMG classification

Our genetic and functional data provide strong evidence that all five *KCND1* group 1 variants are pathogenic. Based on these findings, the evidence of pathogenicity for all group 2 variants was formally evaluated in accordance with ACMG standards and guidelines (Table 3).^39^^,^^40^ For functionally tested variants the criterion PS3 (well-established *in vitro* or *in vivo* functional studies supportive of a damaging effect on the gene or gene product) was rated utilizing a functional assessment table based on significance levels with no reference to the direction and amount of change (i.e., gain or LOF; Table S7). In order to meet the PS3 criterion, at least four electrophysiological items need to be significantly altered, accounting for the possibility that a variant may differ from Kv4.1 WT in only one electrophysiological parameter, but does so in all four tested channel configurations. To keep the scoring system simple, quantitative differences in significant variant effects are not further differentiated. Likewise, all analyzed parameters are assumed to be of equivalent pathogenic significance and are therefore given equal weight. Overall, ACMG classification categorizes three group 2 variants (p.Thr57Met, p.His308Tyr, and p.Asn578Ile) as likely pathogenic, thus providing supporting evidence that these variants are involved in the pathogenesis of a neurodevelopmental disorder. The remaining group 2 missense variants are classified as VUS. Further studies are needed to determine their involvement in pathogenicity.

### Molecular pathophysiology of *KCND1* variants

A closer inspection of the structure-function relationships specifically affected by individual variants may help to understand the molecular pathophysiology of the disorder described herein. Strikingly, the vast majority of *KCND1* missense variants described herein leads to widely scattered amino acid exchanges within the cytoplasmic N- and C-termini at sites that are presumably not quite essential for Kv4.1 channel function (Figures 1 and S1). Although most amino acid exchanges lead to alterations in the number (mostly a reduction) and nature of polar side chain interactions (Figure S2; Table S2), the resultant biophysical alterations are mostly moderate. In contrast, published heterozygous missense variants in *KCND2* associated with a neurodevelopmental disease phenotype alter sites within the S4-S5 linker and the distal S6 segment, core regions of the protein known to play a pivotal role in the operation of the cytoplasmic gate, with a prominent impact on Kv4.2 channel gating.^8^^,^^9^^,^^10^^,^^11^ This obvious difference may reflect natural selection due to the gonosomal and autosomal localization of *KCND1* and *KCND2*, respectively.^34^ Putative *KCND1* missense variants affecting the S4-S5 linker or the distal S6 segment may be selected against because homomeric variant channels may lead to embryonic lethality of males harboring these variants. Of note, all *KCND1* missense-variant-conducting mothers are reportedly clinically asymptomatic. It will be interesting to see if *KCND1* missense variants affecting the cytoplasmic gate are identified in the future and shown to be associated with a female-specific genetic disease.

In order to define loss- or gain-of-function features for a given variant, the direction of changes in biophysical parameters may be considered. Thus, besides an increase in current amplitude, a slowing of macroscopic inactivation, accelerated recovery kinetics, a positively shifted voltage dependence of steady-state inactivation and/or negatively shifted voltage dependence of activation represent gain-of-function features, and vice versa for LOF. Except for the recovery kinetics, which, if modified, were always slowed; biophysical Kv4.1 alterations are observed in either direction. Thus, observed alterations of biophysical parameters are likely to eventually reflect both LOF and gain-of-function. Overall, variant-specific alterations in electrophysiological parameters or a lack of their modulation by auxiliary β subunits do not appear to correlate with the associated clinical phenotype, neither with individual signs, the sum of phenotypic features, nor the disease severity. Notably, this finding holds true regardless of whether the quantitative and qualitative nature of biophysical changes was taken into account or not (data not shown). Therefore, and for the sake of simplicity, we based the PS3 scoring for ACMG classification of *KCND1* variants exclusively on the sum of significantly altered electrophysiological parameters (Tables S3–S7), thus not accounting for the qualitative and quantitative nature of variant effects.

### Cellular mechanism of this neurodevelopmental disorder

In order to determine cellular mechanisms that may be critically influenced by Kv4.1 channel dysfunction or complete LOF, it is important to consider the brain regions in which *KCND1* is expressed and the physiological role of Kv4.1 channels in specific neurons. While *KCND1* expression seems to be relatively moderate compared to other ion channel genes,^34^^,^^49^ it is widely expressed throughout the human brain and has been shown to be involved in the control of slow repetitive action potential firing in many different neurons.^50^^,^^51^^,^^52^^,^^53^ This is reminiscent of the A-type current function originally identified in primitive animals,^54^ where these channels appear to already activate at very negative membrane potentials well below threshold. Thereby, they cause a delay in firing until A-type channel inactivation allows for the next spike. It is obvious that the amount and distribution of A-type channels on the cell surface as well as their biophysical properties, especially their macroscopic inactivation kinetics, are critical for this function. However, none of the reported Kv4.1-associated functional roles, including hippocampal pattern separation,^52^ clock gene expression,^51^ control of striatal circuitry,^53^ or nociception,^50^ appear to directly relate to the neurodevelopmental disease phenotype described herein. Intriguingly, Kv4.1 has also been implicated in the control of cell proliferation, not only in tumor cells,^55^^,^^56^ but also in neural stem and progenitor cells.^57^ Thus, at certain developmental stages, even minor disturbances in Kv4.1 channel levels and/or biophysical properties, representing either gain of function or LOF, may significantly affect neuronal cell proliferation and migration, thus impairing neuronal development. In addition, clock gene expression reportedly under the control of Kv4.1 channel activity^51^ is known to regulate cortical development,^58^ thus representing a further possible explanation for the high phenotypic variability of the described disorder to be explored in the future.

## Data and code availability

Additional methodological, genetic, and clinical data supporting the findings of this study are available from the corresponding authors upon reasonable request. In some cases, specific privacy regulations and the consent under which specific data were obtained may not permit the sharing of additional findings, e.g., raw next-generation sequencing files.

## Web resources

CADD, https://wintervar.wglab.org/
GeneMatcher, https://genematcher.org/
gnomAD database, https://gnomad.broadinstitute.org/
MetaDome, https://stuart.radboudumc.nl/metadome/
PolyPhen-2, http://genetics.bwh.harvard.edu/pph2/
REVEL, https://sites.google.com/site/revelgenomics/
SIFT, https://sift.bii.a-star.edu.sg/

## Supplemental information

Document S1. Figures S1–S5 and Tables S1–S7

Document S2. Article plus supplemental information
