# Supplementary material for: Etiological involvement of KCND1 variants in an X-linked neurodevelopmental disorder with variable expressivity
Source: Am J Hum Genet. 2024 May 20;111(6):1206–21. doi: 10.1016/j.ajhg.2024.04.019 (PMC11179411; doi:10.1016/j.ajhg.2024.04.019)
Supplement: Document S1. Figures S1–S5 and Tables S1–S7 [file mmc1.pdf]

The American Journal of Human Genetics, Volume *111*

Supplemental information

Etiological involvement of *KCND1* variants in an X-linked neurodevelopmental disorder with variable expressivity

Tassja Kalm, Claudia Schob, Hanna Völler, Thatjana Gardeitchik, Christian Gilissen, Rolph Pfundt, Chiara Klöckner, Konrad Platzer, Annick Klabunde-Cherwon, Markus Ries, Steffen Syrbe, Francesca Beccaria, Francesca Madia, Marcello Scala, Federico Zara, Floris Hofstede, Marleen E.H. Simon, Richard H. van Jaarsveld, Renske Oegema, Koen L.I. van Gassen, Sjoerd J.B. Holwerda, Tahsin Stefan Barakat, Arjan Bouman, Marjon van Slegtenhorst, Sara Álvarez, Alberto Fernández-Jaén, Javier Porta, Andrea Accogli, Margherita Maria Mancardi, Pasquale Striano, Michele Iacomino, Jong-Hee Chae, SeSong Jang, Soo Y. Kim, David Chitayat, Saadet Mercimek-Andrews, Christel Depienne, Antje Kampmeier, Alma Kuechler, Harald Surowy, Enrico Silvio Bertini, Francesca Clementina Radio, Cecilia Mancini, Simone Pizzi, Marco Tartaglia, Lucas Gauthier, David Genevieve, Mylène Tharreau, Noy Azoulay, Gal Zaks-Hoffer, Nesia K. Gilad, Naama Orenstein, Geneviève Bernard, Isabelle Thiffault, Jonas Denecke, Theresia Herget, Fanny Kortüm, Christian Kubisch, Robert Bähring, and Stefan Kindler

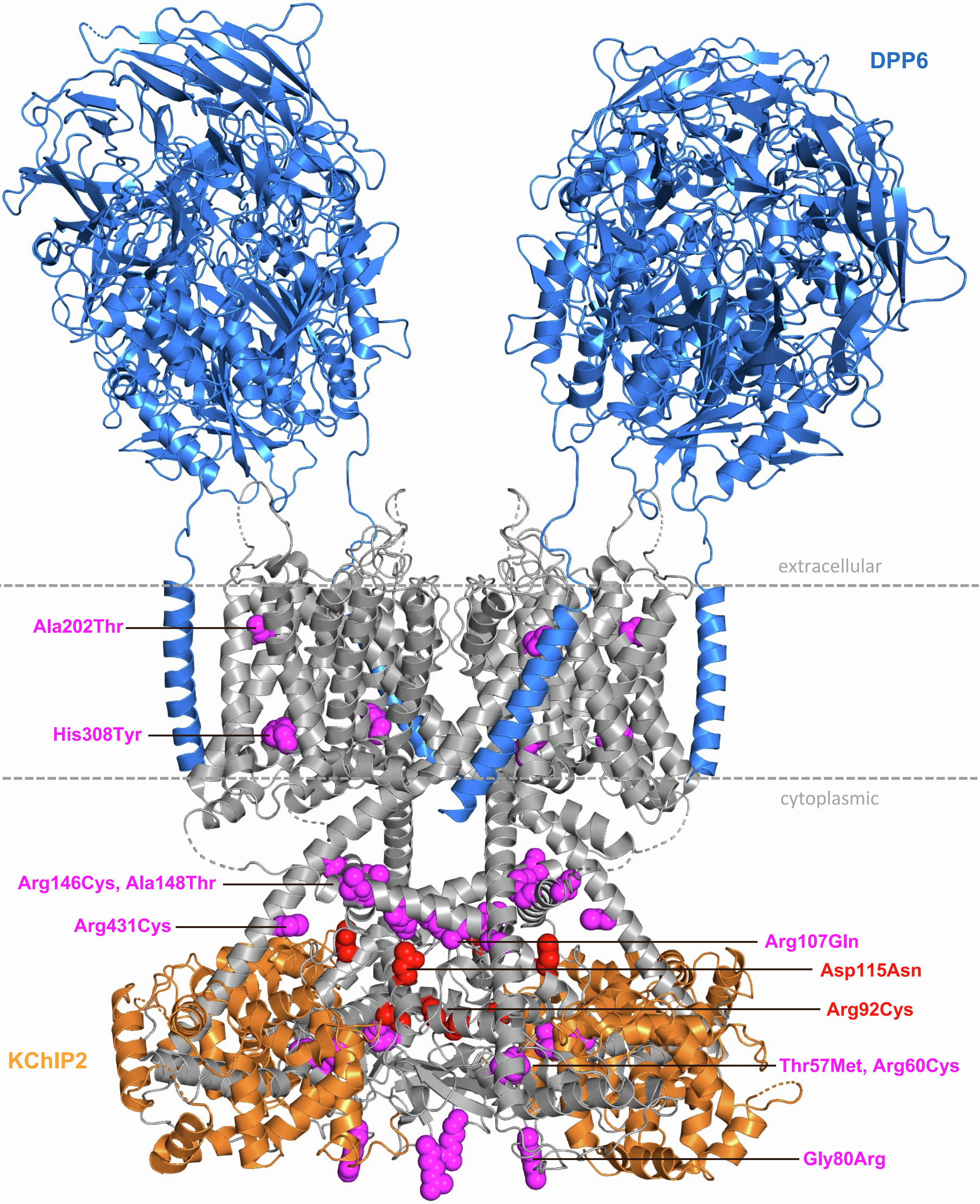

Figure S1. Structure of the ternary Kv4/KChIP/DPP channel complex

The structure of the Kv4.2/KChIP1/DPP6 complex reported by Kise and co-workers (PDB ID 7E8H)[1] was used to give an overview of the ternary channel configuration (α-subunits: grey, KChIPs: orange, DPPs: blue) and the location of *KCND1* variant-associated amino acid substitutions. Amino acid exchanges at sites homologous to Kv4.1 were simulated with the most likely backbone-dependent rotamer orientation using PyMOL (Schrödinger). Protein backbones depicted as ribbon diagrams. Substituted amino acid side chains depicted as red (group 1 variants) or purple (group 2 variants) CPK models. In each case one of the four corresponding substitutions is indicated and labeled (unlabeled structure also available as Movie S1). Note that p.Thr516Ser, p.Arg536Gly and p.Asn578Ile are absent because the structure lacks the C-terminal ends of the α-subunits. Horizontal dotted lines indicate the location of the plasma membrane separating extracellular from cytoplasmic space.

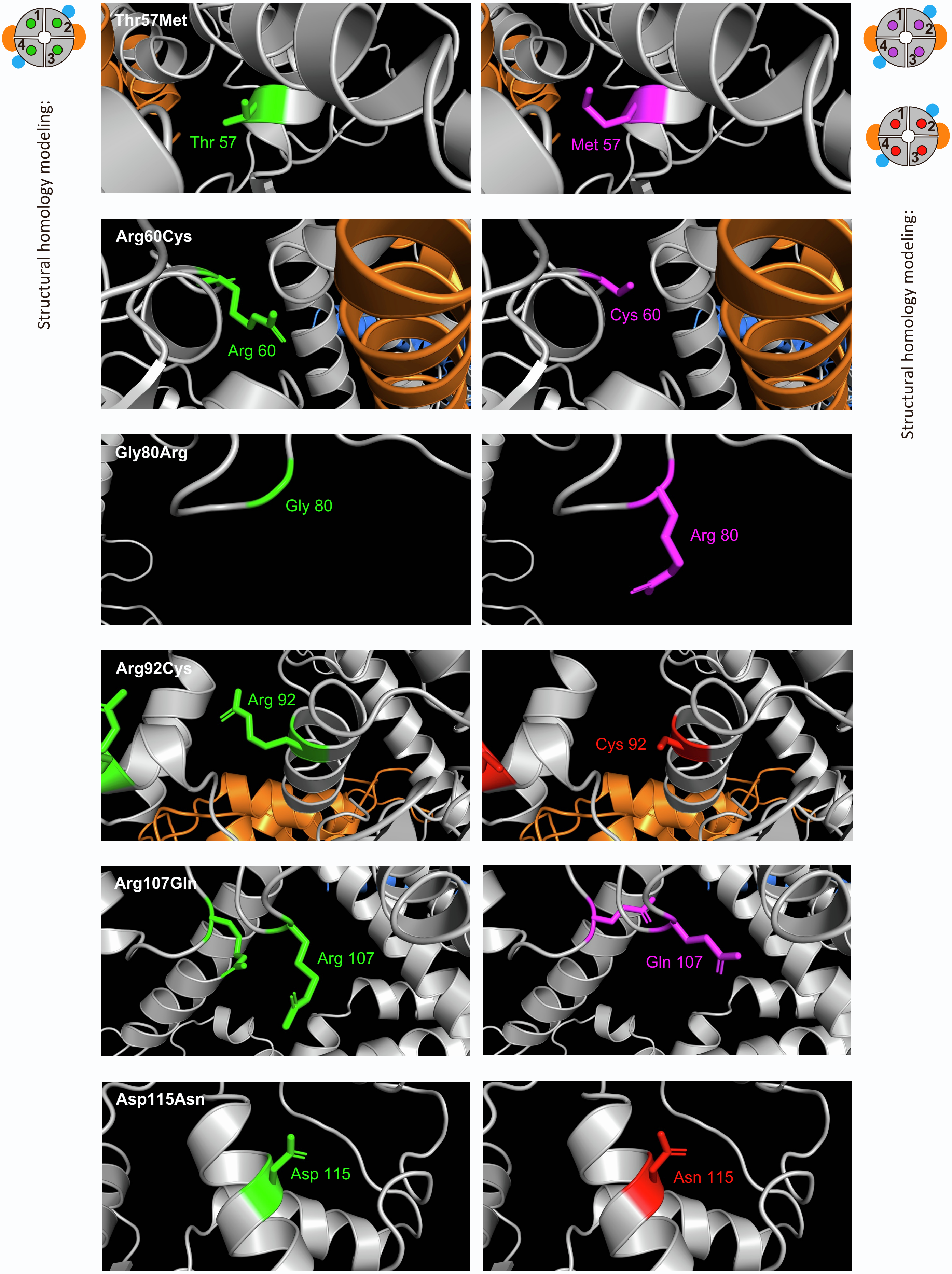
Structural homology modeling:
Thr57Met
Thr 57
Met 57
Arg60Cys
Arg 60
Cys 60
Gly80Arg
Gly 80
Arg 80
Arg92Cys
Arg 92
Cys 92
Arg107Gln
Arg 107
Gln 107
Asp115Asn
Asp 115
Asn 115
Structural homology modeling:

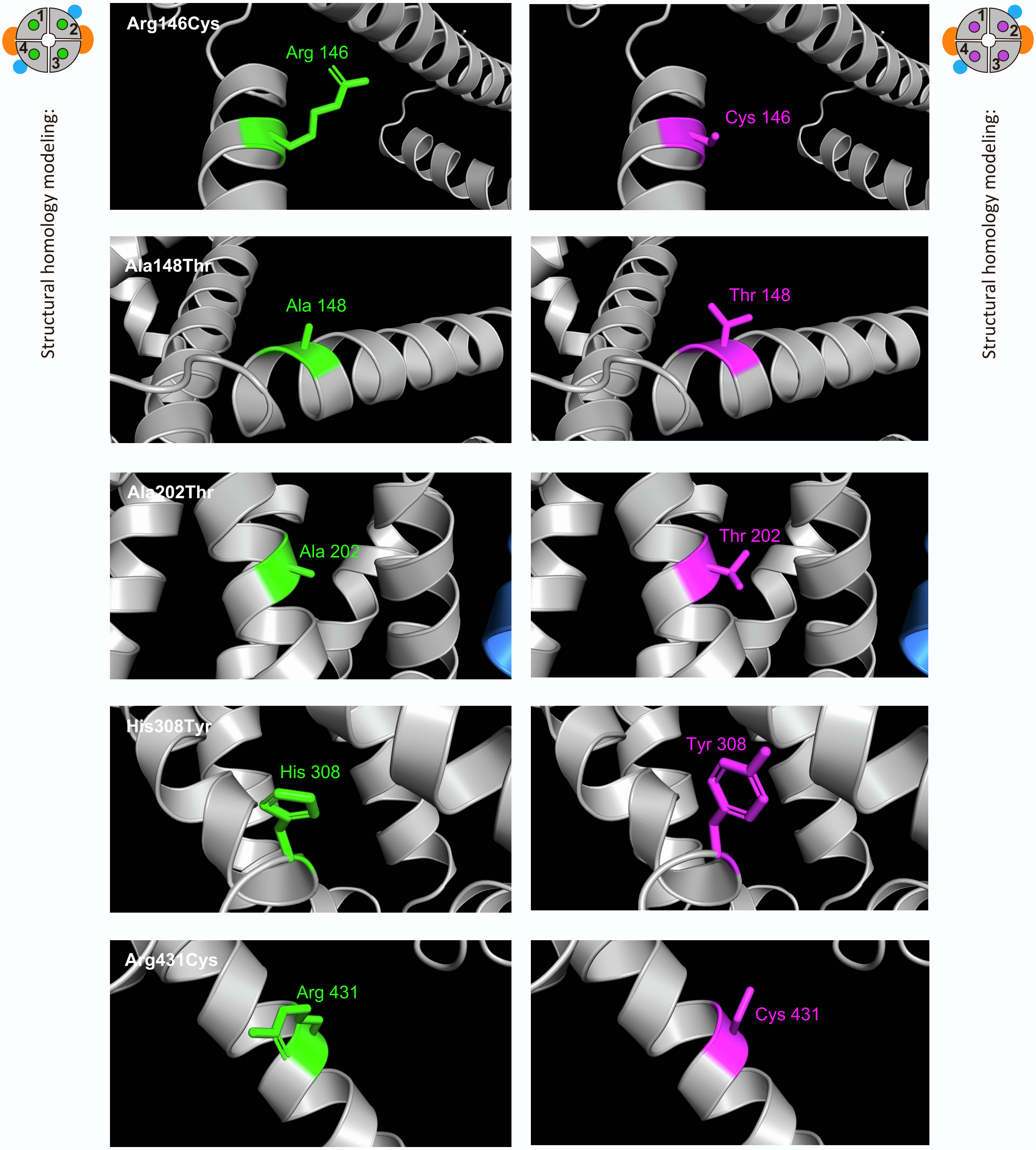

Figure S2. Structural side chain modification caused by *KCND1* variant-associated amino acid substitutions

Structural homology modeling using UniProt (https://www.uniprot.org/), SWISS MODEL (Center for Molecular Life Sciences, University of Basel, Switzerland; https://swissmodel.expasy.org/) and PyMOL (Schrödinger), was performed based on the amino acid sequences of human Kv4.1 (UniProt identifier: Q9NSA2), KChIP2 (UniProt identifier: Q9NS61) and DPP6 (UniProt identifer: P42658), and the ternary Kv4.2/KChIP1/DPP6 structure reported by Kise and co-workers (PDB ID 7E8H).[1] The structure provided by homology modeling was incomplete showing a 4:2:2 stoichiometry due to the lack of two opposite KChIP and DPP molecules (see cartoons). Computer-simulated amino acid substitutions using PyMOL (Schrödinger) were done with consideration of the most likely backbone-dependent rotamer orientation, and the putative steric consequences of the amino acid substitution within a distance of 8 Å were taken into account. Protein backbones depicted as ribbon diagrams. Native (wild-type) and novel variant-associated side chains depicted in stick mode, native residues in green, mutated residues in red (group 1 variants) or purple (group 2 variants).

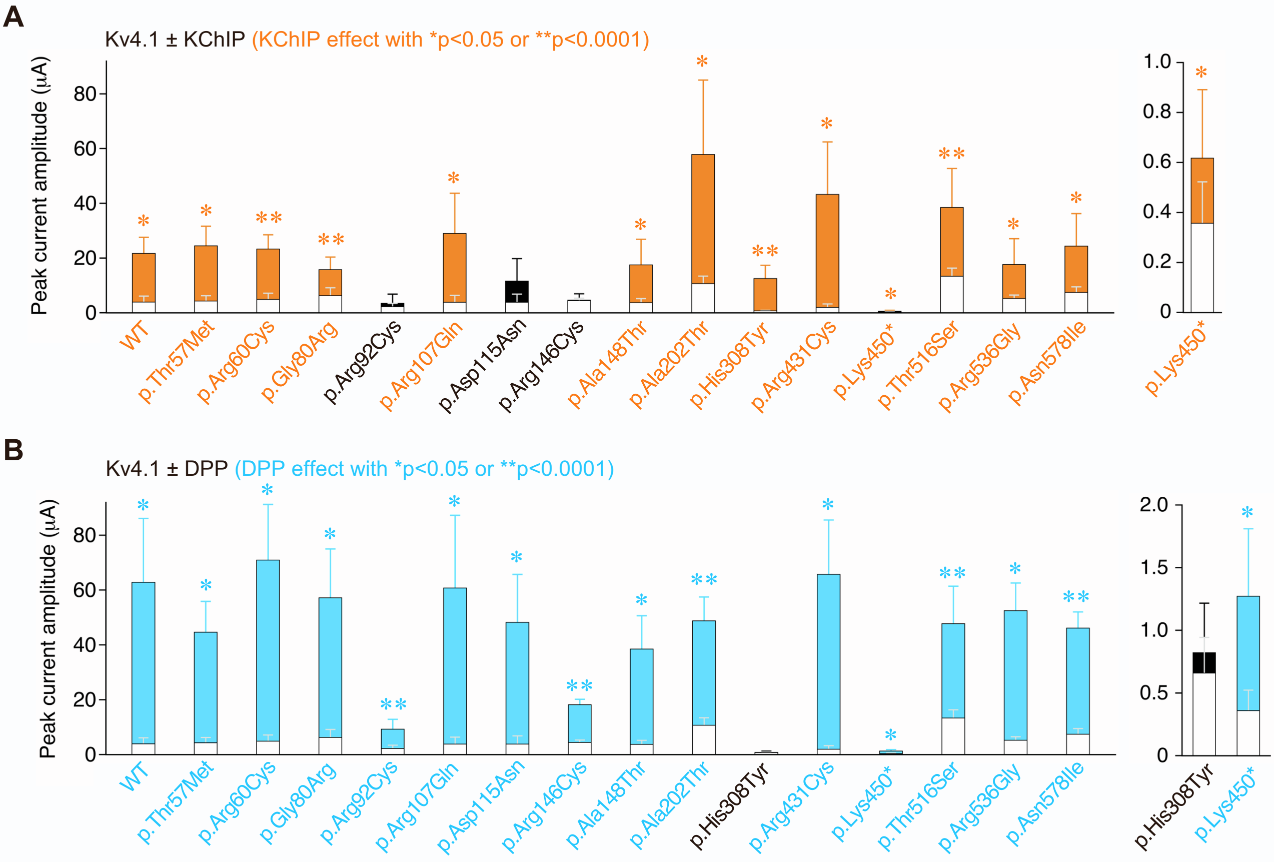

Figure S3. Effects of auxiliary β-subunit co-expression on Kv4.1 channel-mediated peak current amplitudes

Mean peak current amplitudes (data from Tables S4 – S6, error bars are SD) obtained in the absence (empty bars) and presence of either KChIP or DPP, as indicated by the filled bar extensions.

(A) Effects of KChIP co-expression; p.Lys450* data plotted on two different y-scales;

(B) Effects of DPP co-expression; p.His308Tyr and p.Lys450* data plotted on two different y-scales;

Statistics are based on unpaired Student's *t*-tests, applied to the data from Tables S4 – S6; significant effects of auxiliary β-subunit co-expression (compared to Kv4.1 alone) are indicated with * ($p<0.05$) or ** ($p<0.0001$) and by color (significant KChIP effect: orange; significant DPP effect: blue; absence of or non-significant effects: black). Note that the typical incease in peak current amplitude caused by both KChIP and DPP co-expression,[2,3] as seen for Kv4.1 WT and the majority of variants, is absent in the case of p.Arg92Cys, p.Asp115Asn and p.Arg146Cys for KChIP co-expression, and in the case of p.His308Tyr for DPP co-expression.

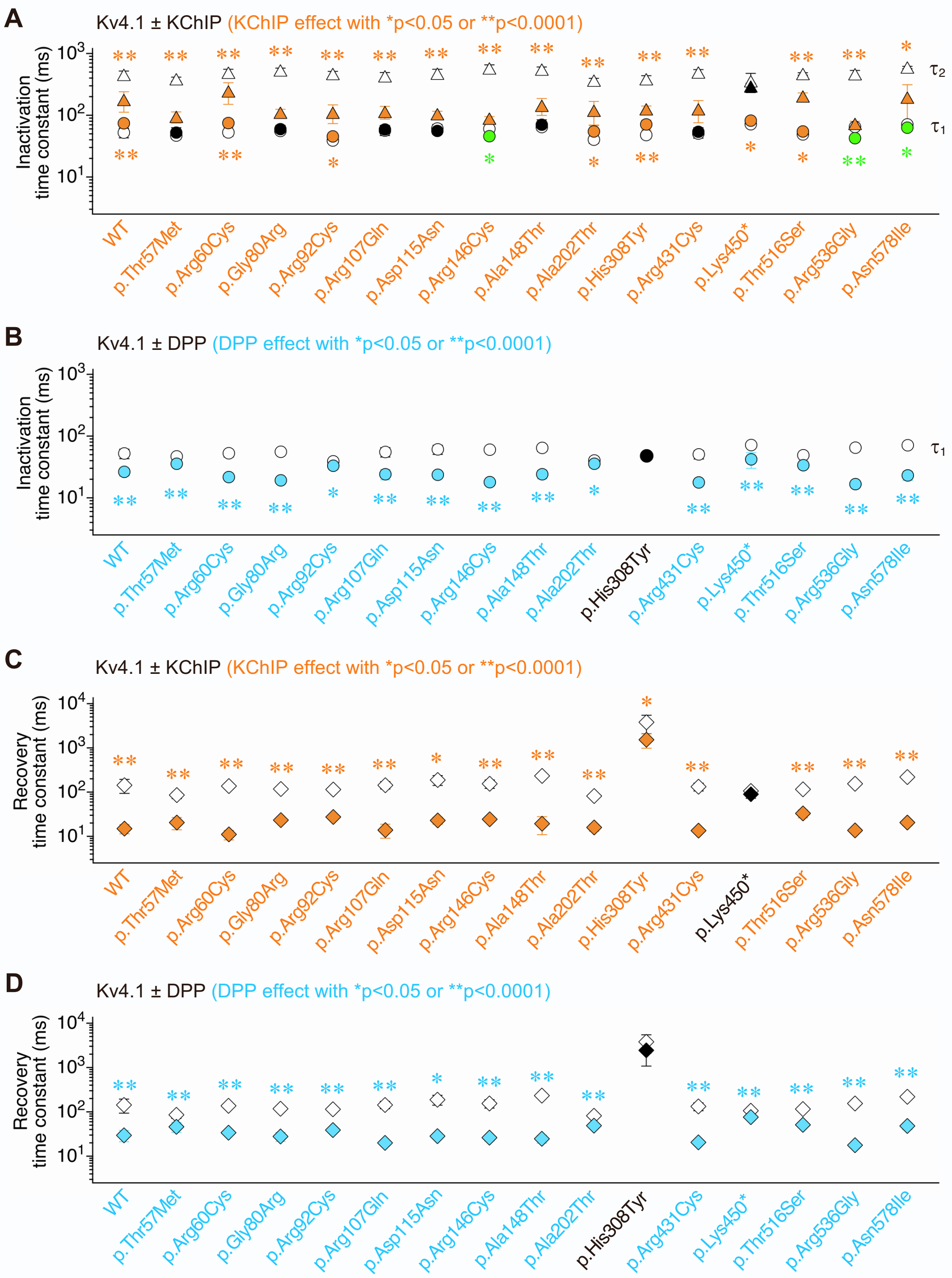

Figure S4. Effects of auxiliary β-subunit co-expression on Kv4.1 channel macroscopic inactivation and recovery kinetics

Mean values of inactivation and recovery time constants (data from Tables S4 – S6, error bars are SD and in many cases smaller than symbols) obtained in the absence (empty symbols) and presence of either KChIP or DPP (filled symbols).

(A and C) Effects of KChIP co-expression;

(B and D) Effects of DPP co-expression;

Statistics are based on unpaired Student's *t*-tests, applied to the data from Tables S4 – S6; significant effects of auxiliary β-subunit co-expression (compared to Kv4.1 alone) are indicated with * ($p<0.05$) or ** ($p<0.0001$) and by color (significant KChIP effect: orange; significant DPP effect: blue; absence of or non-significant effects: black). Typically, KChIP causes a slowing of the initial (fast) and an acceleration of the second (slower) current decay component (τ_1 and τ_2 depicted as circles and triangles, respectively), whereas DPP causes an acceleration of the initial (fast) current decay component (τ_1), while both KChIP and DPP speed up recovery from inactivation.[2,3] These typical modifications were seen for Kv4.1 WT and, at least partially, for the majority of variants. Still, for p.Lys450* KChIP failed to accelerate the recovery kinetics, and for p.His308Tyr none of the typical DPP effects was seen. Notably, in the case of p.Arg146Cys, p.Arg536Gly and p.Asn578Ile KChIP caused an accceleration of the initial current decay component (filled green symbols).

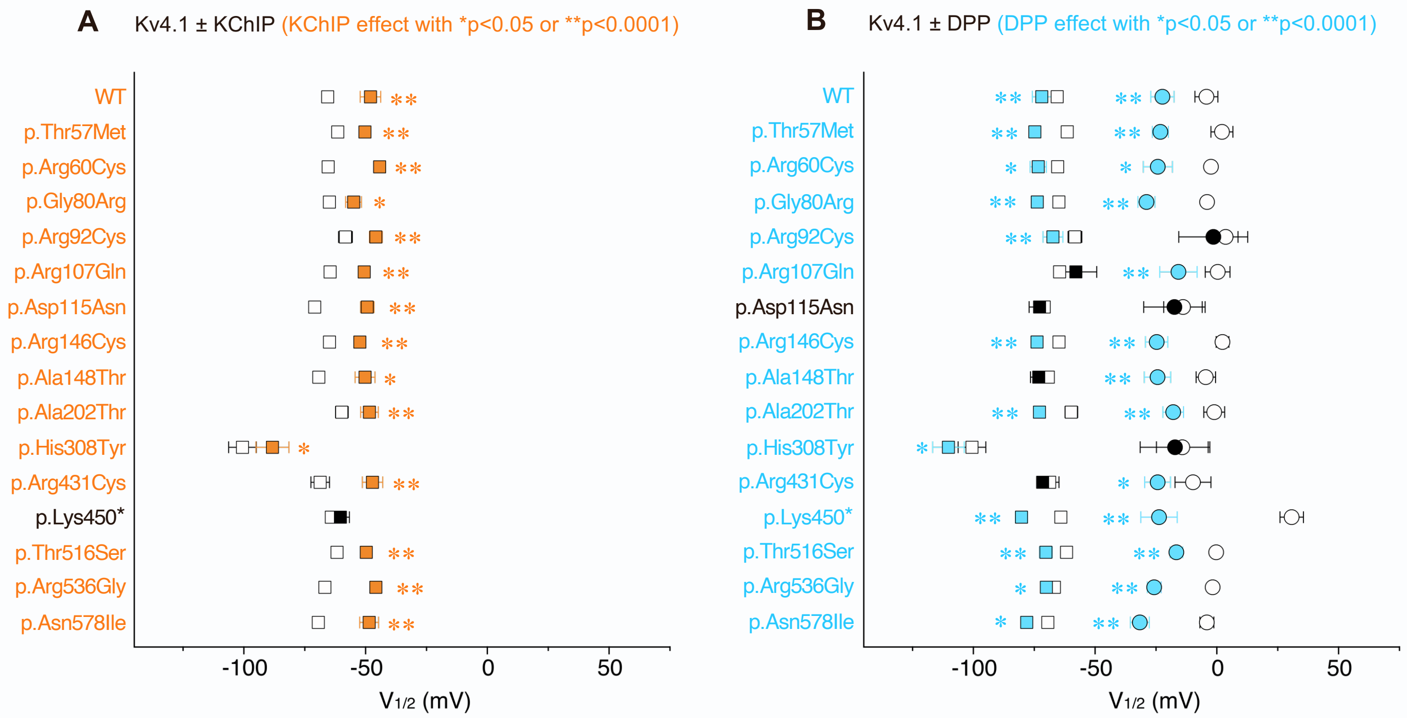

Figure S5. Effects of auxiliary β-subunit co-expression on the voltage dependences of Kv4.1 channel activation and steady-state inactivation

Mean values obtained for the voltages of halfmaximal activation and inactivation (data from Tables S4 – S6, error bars are SD and in many cases smaller than symbols) obtained in the absence (empty symbols) and in the presence of either KChIP or DPP (filled symbols).

(A) Effects of KChIP co-expression;

(B) Effects of DPP co-expression;

Statistics are based on unpaired Student's *t*-tests, applied to the data from Tables S4 – S6; significant effects of auxiliary β-subunit co-expression (compared to Kv4.1 alone) are indicated with * (p<0.05) or ** (p<0.0001) and by color (significant KChIP effect: orange; significant DPP effect: blue; absence of or non-significant effects: black). Typically KChIP causes a positive shift of the inactivation curve, and DPP a negative shift of both activation and inactivation curve,[2,3] as seen for Kv4.1 WT and, at least partially, for the majority of variants. However, in the case of p.Lys450* KChIP failed to significantly shift the inactivation curve in the positive direction, and in the case of p.Asp115Asn DPP failed to significantly shift both curves in the negative direction.

Individual #	1	2	3	4	5	6	7	8	9
Mutation (NM_004979.6; NP_004970.3)	c.274C>T (p.Arg92Cys)	c.343G>A (p.Asp115Asn)	c.182_194del (p.Tyr61Cysfs*31)	c.295C>T (p.Arg99*)	c.1348A>T (p.Lys450*)	c.170C>T (p.Thr57Met)	c.178C>T (p.Arg60Cys)	c.238G>C (p.Gly80Arg)	c.320G>A (p.Arg107Gln)
Origin	*de novo*	*de novo*	MI	MI	MI	MI	MI	MI	MI
Additional candidate variants	none	none	none	none	none	*TCEAL2*: c.595C>T (p.Gln199*), NM_080390.4, homozygous	none	*AREG*: c.440delA (p.Asn147Metfs*16), NM_001657.4, heterozygous	none
Birth weight (z, date of birth)	RN (40.5 wks)	ND (39.7 wks)	RN (at term)	2680 g (-0.79, 36.7 wks)	3262 g (-0.99, 40.7 wks)	4210 g (1.33, 40 wks)	3000 g (-1.25, 39.5 wks)	2100 g (-0.57, 34 wks)	2350 g (-2.2, 38 wks)
Birth length (z)	ND	ND	RN	ND	52 cm (-0.4)	ND	ND	ND	ND
OFC birth (z)	ND	ND	RN	ND	ND	ND	ND	ND	ND
Age last exam	15 y	9 y 11 m	16 y	16 y	3 y 2m	5 y 6 m	3 y 6 m	16 y	7 y
Weight last exam (z)	74 kg (1.1)	28.6 kg (-0.34)	ND	45.6 kg (-2.34)	15.5 kg (0.25)	23.5 kg (0.95)	15.9 kg (0.14)	46 kg (-2.28)	ND
Height last exam (z)	185.5 cm (1.54)	132.5 cm (-0.56)	ND	169 cm (-1.02)	96.4 cm (-0.5)	118 cm (0.58)	99.2 cm (-0.29)	166 cm (-1.42)	ND
OFC at last exam (z)	ND	55.5 cm (1.71)	ND	RN	50.7 cm (-0.09)	52 cm (0.07)	50.5 cm (-0.36)	54 cm (-1.57)	ND
Age at first signs	10 y	6 y	2 y 3 m	abt 2 y	neonatal period	abt 3 y	abt 2 y	3 y	4 y 10 m
First signs	anxiety, tics	speech delay, seizures	seizures	speech delay, motor delay, anxiety	speech delay, motor delay	speech delay, mild motor delay	speech delay mild motor delay	speech delay, autistic features	seizures (motor, with impaired awareness)
Motor development	normal	normal	normal	delayed	delayed	mildly delayed	mildly delayed	normal	normal
Intellectual development	average	low average, TIQ 85	average	borderline mental functioning – low average, TIQ 74-86	extremely low/impaired, TIQ 68	WNV of Bayley-III-NL could not be completed, developmental age estimate at 53 m of age: 20-42 m	extremely low/impaired, TIQ 55	extremely low/impaired, TIQ <50, WISC-IV 43 (7 y), WISC-V 41 (13 y)	average
Speech development	normal	delayed	normal	delayed	delayed	delayed	delayed	delayed, pragmatic language disorder	mildly delayed
Receptive language	normal	below average	normal	below average	below average	poor	ND	ND	ND
Comprehension	normal	below average	normal	below average	below average	below average	below average	below average	below average
Neuropsychiatric signs	ASD, attention deficit, anxiety, tics	attention deficit	none	ASD, anxiety, emotional problems	attention deficit	poor social skills, little contact, attention deficit	past temper tantrums	ASD, hyperactivity, attention deficit	hyperactivity, attention deficit
Ambulation/ gait	normal	normal	normal	normal	normal	clumsy gait[a]	normal	normal	normal
Neurological and muscular features	none	mild hypotonia	none	ND	none	none	probably mild hypotonia in infancy	hypotonia	none
Seizures/epilepsy	no	epilepsy, absences, myoclonus, eyelid myoclonia	epilepsy, generalized tonic-clonic seizures during sleep until 11 y, then seizures remitted, Valproate responsive	no	no	no	no	no	epilepsy, multiple daily seizures, psychomotor arrest, eye deviation
EEG	normal	irregular polyspikes and spike-waves, photoparoxysmal reaction	generalized abnormalities at 3 y 8 m, normal at 12 y 6 m	ND	ND	ND	ND	normal	irregular polyspikes and spike-waves
Cerebral MRI	normal	normal	normal	ND	ND	ND	ND	normal	ND

**Individual #**	10	11	12	13	14[b]	15[c]	16[c]	17	18
Mutation (NM_004979.6; NP_004970.3)	c.436C>T (p.Arg146Cys)	c.442G>A (p.Ala148Thr)	c.604G>A (p.Ala202Thr)	c.922C>T (p.His308Tyr)	c.1291C>T (p.Arg431Cys)	c.1546A>T (p.Thr516Ser)	c.1546A>T (p.Thr516Ser)	c.1606A>G (p.Arg536Gly)	c.1733A>T (p.Asn578Ile)
Origin	MI	MI	MI	MI	MI	MI	MI	MI	MI
Additional candidate variants	none	none	*EIF2B3*: c.172delG (p.Asp58Metfs*12), NM_020365.5, heterozygous; *SYNE1*: c.18891+2T>C, NM_182961.4, heterozygous	*HAGHL*: c.55G>A (p.Glu19Lys), NM_207112.2, heterozygous	none	none	none	none	*AMER1*: c.3112C>T (p.Pro1038Ser), NM_152424.4, hemizygous
Birth weight (z, date of birth)	ND (at term)	2300 g (-1.26, 37 wks)	3543 g (0.13, 39 wks)	2650 g (-1.81, 38.7 wks)	3300 g (-0.14, at term)	3470 g (-0.38, 40 wks)	2680 g (-1.86, 39 wks)	3180 g (-0.16, 37.7 wks)	2050 g (-2.37, 37 wks)
Birth length (z)	ND	ND	52 cm (0.04)	48 cm (-1.6)	51 cm (-0.14)	50 cm (-1.12)	46 cm (-2.57)	48 cm (-1.12)	46 cm (-1.75)
OFC birth (z)	ND	ND	35 cm (-0.23)	34 cm (-0.9)	35 cm (-0.01)	34.5 cm (-0.88)	34 cm (-1)	ND	31 cm (-2.27)
Age last exam	21 y	5 y	6 y 11 m	1 y 9 m	8 m	11 y 8 m	6 y 4 m	10 y 1 m	3 y
Weight last exam (z)	88 kg	18 kg (-0.49)	23.8 kg (-0.03)	13.7 kg (1.03)	5.6 kg (-2.98)	47.3 kg (0.78)	24.3 kg (0.51)	38.5 kg (0.63)	ND
Height last exam (z)	ND	110 cm (-0.36)	ND	90 cm (1.26)	65 cm (-2.16)	158 cm (1.05)	125 cm (0.82)	138.5 cm (-0.52)	ND
OFC at last exam (z)	ND	50 cm (-1.3)	45.5 cm (-5.38)	48.5 cm (-0.47)	40 cm (-4.37)	54.3 cm (-0.02)	52.5 cm (0.16)	54.2 cm (0.4)	ND
Age at first signs	abt 3 y	1 y 6 m	3 m	2 m	at birth	1 y 8 m	5-6 m	neonatal period	at birth (prenatal)
First signs	speech delay, ID	speech delay, poor eye contact	seizures	seizures	hypotonia	speech delay	feeding issues	feeding issues	IUGR, PAPP-A, postnatal SGA, pneumothorax
Motor development	normal	normal	delayed	normal	delayed	normal	normal	delayed	delayed
Intellectual development	extremely low/impaired, FSIQ 40-50	average, WPPSI-IV 90	impaired, not formally examined due to severe developmental delay	average	impaired, not formally examined, severe developmental delay and early death	borderline mental functioning, FSIQ 77	borderline mental functioning, FSIQ 77	low average, TIQ 81[d]	impaired, not formally examined
Speech development	delayed	delayed	delayed, never acquired ability to speak	normal	ND	delayed	delayed, never acquired ability to speak	delayed	delayed
Receptive language	ND	below average	poor	normal	ND	below average	poor	normal	poor
Comprehension	below average	below average	below average	normal	ND	below average	below average	normal	below average
Neuropsychiatric signs	none	none	ND	none	ND	none	ASD	ASD, tics	ASD
Ambulation/ gait	normal	normal	never acquired ambulation	normal	ND	normal	normal	initially normal[e]	unsteady gait
Neurological and muscular features	none	none	generalized hypotonia	none	hypotonia	none	none	slight hypotonia	hypotonia (limbs)
Seizures/epilepsy	epilepsy, generalized tonic-clonic seizures, Oxcarbazepine & Levetiracetam responsive	no	epilepsy, frequent recurrent seizures, nonresponsive to multiple anti-epileptic drugs	epilepsy, generalized tonic-clonic seizures, frequent recurrent febrile and non-febrile seizures, no medication given	no	no	no	no	no
EEG	few low amplitude spike discharges on left centro-temporal area	normal	abnormal background with intermittent epileptiform discharges mainly in bifrontal regions	normal	normal	ND	ND	normal	normal
Cerebral MRI	normal	normal	corpus callosum absent, progressive cerebral atrophy with relative sparing of basal ganglia and cerebellum, myelination arrest at 5 m	unremarkable at 2 m, enlarged external ventricles supratentorial	hypomyelination, abnormal symmetrical central tegmental tract hyper-intensities, increased DWI signal of bilateral central tegmental tract	hypoplasia of corpus callosum	small corpus callosum (3rd centile)	ND	bilateral small heterotopia in centrum semiovale

Table S1. Key clinical features of subjects with hemizygous *KCND1* variants (group 1 and group 2)

Z-scores for birth and growth parameters were calculated using the Ped(z) Pediatric Calculator (https://www.pedz.de/en/welcome.html). Abbreviations: abt, about; ASD, autism spectrum disorder; DD, developmental delay; exam, examination; DWI, diffusion-weighted magnetic resonance imaging; FSIQ, full scale intelligence quotient; ID, intellectual disability; IUGR, intrauterine growth retardation; m, months; MI, maternally inherited; ND, no data; OFC, occipitofrontal head circumference; PAPP-A, pregnancy-associated plasma protein A; RN, reported normal; SD, standard deviation; SGA, small for gestational age; TIQ, total intelligence quotient; WISC, Wechsler intelligence scale for children; WNV, Wechsler nonverbal scale of ability; wks, weeks; WPPSI, Wechsler preschool and primary scale of intelligence; y, years; z, z-score. [a]No clear evidence of ataxia, no broad-based gait, no coordination problems, no muscle weakness or hypertonia, but rather a somewhat unstable gait pattern possibly reflecting hypermobility or a general gross motor delay, [b]Died at eight months due to respiratory insufficiency, probably linked to progressive hypo-myelinating encephalopathy, [c]Brothers of different age, [d]At ten years of age, decline probably due to suspected childhood disintegrative disorder, [e]Increasingly frequent use of a wheel chair since the onset of suspected childhood disintegrative disorder

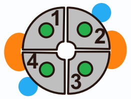

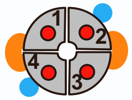

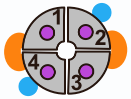

Structural homology modeling:

KCND1	side chain → side chain	side chain → backbone	backbone → side chain
Thr57<br>Met57	Thr53 (4-4-3.1Å)<br>—	Trp54 (4-4-2.9Å)<br>—	—<br>—
Arg60<br>Cys60	Asn56 (1-1-2.8Å)<br>—	Gly4 (1-1-3.1Å); Asn56 (4-4-2.8Å)<br>—	—<br>—
Gly80<br>Arg80	—<br>—	—<br>—	—<br>—
Arg92<br>Cys92	Asp87 (1-4-2.6Å, 4-3-2.8Å); Asp89 (1-1-2.7Å, 4-4-2.6Å)<br>—	—<br>—	Asn96 (1-1-3.0Å, 4-4-3.3Å)<br>Asn96 (1-1-2.9Å, 4-4-3.3Å)
Arg107<br>Gln107	Gln108 (1-3-2.9Å)<br>Arg139 (1-4-2.9Å,3.1Å)	Arg107 (1-4-3.5Å, 4-3-2.7Å); Glu109 (4-3-2.9Å)<br>—	Arg107 (1-2-2.7Å)<br>Arg139 (4-4-3.3Å)
Asp115<br>Asn115	Tyr136 (4-4-2.6Å)<br>Tyr136 (4-4-2.8Å); Lys140 (1-1-2.6Å; 4-4-3.0Å)	—<br>Asn115 (1-1-2.8Å)	—<br>—
Arg146<br>Cys146	Glu134 (1-2-2.7Å); Asp130 (4-1-2.7Å); Glu142 (4-4-2.7Å)<br>—	Asp130 (4-1-3.0Å)<br>—	Arg424 (4-4-2.9Å)<br>Arg424 (4-4-2.9Å)
Ala148<br>Thr148	—<br>—	—<br>Ala144 (1-1-3.0Å, 4-4-2.8Å)	—<br>—
Ala202<br>Thr202	—<br>—	—<br>Val198 (1-1-2.7Å, 4-4-2.8Å)	—<br>—
His308<br>Tyr308	—<br>—	Tyr189 (1-1-2.8Å); His308 (1-1-3.0Å)<br>—	—<br>—
Arg431<br>Cys431	Gln428 (4-4-2.7Å)<br>—	Glu126 (1-2-3.3Å)<br>—	—<br>—

Table S2. Polar contacts involving side chains in a modeled Kv4.1/KChIP2/DPP6 structure

A computer search for polar interactions involving side chains was performed on the Kv4.1/KChIP2/DPP6 structure provided by homology modeling (see cartoons: 4 α-subunits, grey; 2 KChIPs, orange; 2 DPPs, blue; see also Figure S2). Amino acid substitutions were modeled and searches for polar side chain interactions including distance measurements were performed for wild-type and variant α-subunits. The results obtained for the residues of interest (wild-type: green; group 1 mutants: red; group 2 mutants: purple) in α-subunits 1 and 4 are illustrated. Numbers separated by dashes represent (in this order): subunit of the residue under study (1 or 4) – subunit of the interaction partner – smallest distance between interacting atoms. All three possible combinations of side chain and backbone involvement are considered. Note that in the majority of cases the amino acid substitution leads to a reduction in the number of contacts.

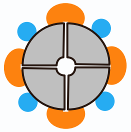

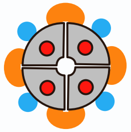

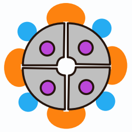

Heterologous expression:

**Kv4.1 + KChIP + DPP**	I_{+40} (μA)	τ_1 (ms)	τ_2 (ms)	% τ_1	τ_{rec} (ms)	$V_{1/2,inact}$ (mV)	k_{inact} (mV)	$V_{1/2,act}$ (mV)	k_{act} (mV)
WT	19.2 ± 9.5 (n = 16)	30.7 ± 4.7	85.8 ± 56.8	82.6 ± 10.8 (n = 21)	11.2 ± 2.0 (n = 16)	-57.9 ± 4.3	7.42 ± 2.00 (n = 18)	-6.5 ± 5.4	25.2 ± 2.2 (n = 17)
p.Thr57Met	17.6 ± 13.6 (n = 9)	26.8 ± 2.5	107 ± 36	93.6 ± 3.8 (n = 9)	10.6 ± 1.0 (n = 9)	-57.0 ± 2.8	7.32 ± 1.40 (n = 9)	-2.5 ± 2.7	24.2 ± 1.4 (n = 9)
p.Arg60Cys	23.2 ± 13.3 (n = 14)	27.1 ± 5.2	102 ± 49	91.7 ± 5.9 (n = 14)	9.80 ± 0.45 (n = 10)	-56.2 ± 2.5	7.31 ± 0.96 (n = 9)	-1.1 ± 3.6	28.5 ± 2.3 (n = 9)
p.Gly80Arg	15.8 ± 6.6 (n = 10)	25.4 ± 3.5	55.4 ± 22.1	76.2 ± 15.7 (n = 10)	9.74 ± 1.43 (n = 8)	-60.0 ± 3.0	7.23 ± 2.10 (n = 9)	-9.9 ± 3.4	24.7 ± 2.8 (n = 10)
pArg92Cys	5.47 ± 3.63 ** (n = 21)	36.3 ± 4.3 *	150 ± 83 *	83.3 ± 15.2 (n = 20)	12.2 ± 1.4 (n = 19)	-57.2 ± 2.5	6.49 ± 0.88 (n = 16)	-6.0 ± 5.3	21.5 ± 1.8 * (n = 12)
p.Arg107Gln	22.4 ± 7.7 (n = 13)	21.2 ± 2.4 **	54.5 ± 18.7	82.8 ± 16.6 (n = 13)	10.1 ± 3.3 (n = 8)	-57.1 ± 3.0	6.87 ± 1.53 (n = 9)	-4.8 ± 3.6	26.3 ± 1.9 (n = 8)
p.Asp115Asn	9.22 ± 4.36 * (n = 11)	18.4 ± 2.8 **	35.8 ± 5.5 *	70.1 ± 17.4 (n = 11)	11.3 ± 2.5 (n = 8)	-61.7 ± 2.3	6.32 ± 0.42 (n = 8)	-6.8 ± 2.4	24.3 ± 2.6 (n = 8)
p.Arg146Cys	4.22 ± 1.62 * (n = 10)	21.0 ± 2.4 **	40.9 ± 8.1	65.4 ± 13.6 * (n = 10)	11.5 ± 1.6 (n = 8)	-61.8 ± 2.3	8.48 ± 0.76 (n = 6)	-0.5 ± 7.6	24.2 ± 3.7 (n = 7)
p.Ala148Thr	12.5 ± 3.1 (n = 10)	26.0 ± 2.3	47.1 ± 6.0	63.7 ± 10.8 * (n = 10)	12.4 ± 1.4 (n = 8)	-61.2 ± 4.2	7.14 ± 0.60 (n = 8)	-8.2 ± 4.8	24.5 ± 2.5 (n = 8)
p.Ala202Thr	20.8 ± 12.2 (n = 13)	33.5 ± 5.8	82.0 ± 19.3	81.0 ± 12.0 (n = 13)	11.0 ± 1.5 (n = 9)	-53.3 ± 5.3 *	10.7 ± 3.6 ** (n = 9)	+3.1 ± 6.2 *	27.6 ± 2.7 (n = 10)
p.His308Tyr	2.30 ± 1.95 ** (n = 12)	57.0 ± 13.1 **	116 ± 35	41.2 ± 27.9 ** (n = 12)	937 ± 293 ** (n = 9)	-98.6 ± 6.5 **	7.79 ± 2.80 (n = 9)	-22.9 ± 17.2 **	33.2 ± 8.4 ** (n = 9)
p.Arg431Cys	18.2 ± 9.8 (n = 10)	29.6 ± 2.4	75.8 ± 14.9	83.2 ± 10.0 (n = 9)	11.5 ± 1.4 (n = 9)	-59.7 ± 5.2	6.85 ± 1.03 (n = 8)	-7.7 ± 5.6	25.7 ± 2.9 (n = 9)
p.Lys450*	1.20 ± 0.75 ** (n = 12)	35.8 ± 5.7 *	156 ± 30 **	60.8 ± 7.6 ** (n = 15)	38.7 ± 4.5 (n = 9)	-67.7 ± 3.1 **	7.17 ± 1.24 (n = 7)	-10.7 ± 6.9	24.4 ± 3.8 (n = 7)
p.Thr516Ser	19.1 ± 8.2 (n = 15)	46.1 ± 4.3 **	146 ± 17 *	72.8 ± 3.7 (n = 14)	18.7 ± 2.5 (n = 13)	-46.0 ± 2.0 **	6.70 ± 1.16 (n = 11)	+4.4 ± 5.3 *	23.1 ± 3.1 (n = 12)
p.Arg536Gly	17.6 ± 9.6 (n = 12)	32.1 ± 4.0	88.4 ± 45.5	86.0 ± 10.6 (n = 12)	10.7 ± 1.3 (n = 9)	-59.2 ± 2.9	7.24 ± 1.18 (n = 9)	-11.6 ± 3.2	25.1 ± 0.9 (n = 9)
p.Asn578Ile	21.9 ± 9.8 (n = 12)	38.5 ± 5.1 *	125 ± 78	88.5 ± 11.0 (n = 12)	11.7 ± 2.4 (n = 9)	-58.6 ± 2.7	8.62 ± 2.87 (n = 8)	-14.6 ± 4.3	27.5 ± 2.1 (n = 7)

Table S3. Biophysical parameters for Kv4.1 wild-type and variant ternary channels

Kv4.1 channels (grey, variant channels with red or purple dots) were studied in a ternary configuration; i.e. in the presence of both KChIP (orange) and DPP (blue). Mean values ± SD and number of observations (n) are given for the peak current amplitude at + 40 mV (I_{+40}), the time constants of macroscopic inactivation (τ_1 and τ_2) including the fractional amplitude of τ_1 (%), the time constant of recovery from inactivation (τ_{rec}) and the voltages of halfmaximal inactivation ($V_{1/2,inact}$) and activation ($V_{1/2,act}$) with corresponding slope factors (k_{inact} and k_{act}, respectively). Statistics are based on one-way analysis of variance (ANOVA) with Dunnett's post hoc testing. For significant variant effects (i.e., deviations from Kv4.1 WT ternary; key variants: red, maternally inherited missense variants: purple) significance levels are indicated with * (p<0.05) or ** (p<0.0001); shaded fields: no significant differences found compared to Kv4.1 WT ternary.

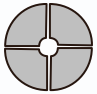

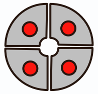

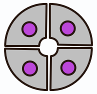

Heterologous expression:

Kv4.1	I_{+40} (μA)	τ_1 (ms)	τ_2 (ms)	% τ_1	τ_{rec} (ms)	$V_{1/2,inact}$ (mV)	k_{inact} (mV)	$V_{1/2,act}$ (mV)	k_{act} (mV)
WT	3.94 ± 2.24 (n = 46)	52.1 ± 9.3	452 ± 67	67.3 ± 5.6 (n = 45)	143 ± 50 (n = 22)	-65.6 ± 2.3	7.10 ± 1.71 (n = 24)	-4.3 ± 4.7	22.3 ± 2.1 (n = 25)
p.Thr57Met	4.25 ± 2.02 (n = 13)	46.9 ± 6.0	382 ± 29 *	72.3 ± 1.7 * (n = 13)	85.3 ± 14.2 (n = 8)	-61.5 ± 2.0 *	7.72 ± 1.53 (n = 8)	+2.1 ± 4.5 *	21.5 ± 2.8 (n = 7)
p.Arg60Cys	4.91 ± 2.26 (n = 17)	52.5 ± 6.5	487 ± 65	67.8 ± 5.3 (n = 16)	137 ± 18 (n = 8)	-65.4 ± 0.9	7.60 ± 2.05 (n = 9)	-2.4 ± 1.9	23.7 ± 0.7 (n = 6)
p.Gly80Arg	6.29 ± 2.90 * (n = 16)	55.4 ± 5.6	528 ± 51 *	64.6 ± 3.6 (n = 20)	117 ± 13 (n = 9)	-64.9 ± 1.2	9.04 ± 2.72 * (n = 10)	-4.0 ± 1.8	24.1 ± 0.7 (n = 4)
p.Arg92Cys	2.20 ± 1.28 * (n = 26)	38.7 ± 1.8 **	467 ± 45	72.9 ± 1.3 * (n = 17)	114 ± 13 (n = 11)	-58.3 ± 2.8 **	6.11 ± 0.35 (n = 10)	+3.6 ± 5.1 *	22.0 ± 2.0 (n = 11)
p.Arg107Gln	3.80 ± 2.56 (n = 66)	56.4 ± 9.7	424 ± 64	67.4 ± 5.1 (n = 39)	144 ± 27 (n = 11)	-64.5 ± 2.3	8.65 ± 1.72 (n = 16)	+0.4 ± 5.1	23.9 ± 2.9 (n = 13)
p.Asp115Asn	3.86 ± 2.95 (n = 33)	60.5 ± 10.1 **	469 ± 85	68.5 ± 5.2 (n = 31)	188 ± 47 (n = 5)	-70.9 ± 1.3 **	9.85 ± 1.52 * (n = 8)	-13.9 ± 7.9 *	20.8 ± 4.2 (n = 4)
p.Arg146Cys	4.46 ± 0.92 (n = 12)	60.0 ± 6.9 *	573 ± 81 **	72.4 ± 3.7 * (n = 20)	155 ± 33 (n = 9)	-64.9 ± 1.3	10.7 ± 3.0 ** (n = 9)	+2.3 ± 2.6	26.5 ± 2.4 * (n = 5)
p.Ala148Thr	3.70 ± 1.53 (n = 19)	64.2 ± 7.7 **	546 ± 57 **	69.0 ± 4.4 (n = 19)	231 ± 19 (n = 8)	-69.2 ± 1.0 *	6.85 ± 0.33 (n = 8)	-4.5 ± 4.0	23.3 ± 1.8 (n = 10)
p.Ala202Thr	10.7 ± 2.72 ** (n = 12)	40.3 ± 5.3 **	361 ± 35 *	73.8 ± 2.7 * (n = 12)	81.9 ± 9.8 (n = 7)	-59.8 ± 2.6 **	6.77 ± 0.83 (n = 9)	-1.1 ± 4.3	21.2 ± 1.1 (n = 9)
p.His308Tyr	0.66 ± 0.28 ** (n = 12)	47.9 ± 5.9	385 ± 51 *	65.6 ± 4.7 (n = 12)	3760 ± 1650 ** (n = 10)	-100.6 ± 5.7 **	7.16 ± 2.32 (n = 8)	-14.2 ± 10.6 *	24.8 ± 5.3 (n = 7)
p.Arg431Cys	1.98 ± 1.23 * (n = 16)	50.3 ± 8.0	492 ± 56	67.7 ± 3.9 (n = 25)	132 ± 27 (n = 9)	-68.6 ± 3.8 *	9.21 ± 1.76 * (n = 8)	-9.8 ± 7.4	22.7 ± 2.3 (n = 4)
p.Lys450*	0.36 ± 0.17 ** (n = 19)	71.9 ± 6.5 **	354 ± 123 **	73.9 ± 6.0 * (n = 12)	106 ± 7 (n = 5)	-64.1 ± 1.8	9.43 ± 0.62 (n = 4)	+30.7 ± 4.8 **	34.1 ± 4.0 ** (n = 5)
p.Thr516Ser	13.3 ± 3.07 ** (n = 20)	48.8 ± 3.9	464 ± 20	68.7 ± 1.8 (n = 20)	115 ± 8 (n = 6)	-61.7 ± 1.9 *	7.18 ± 0.28 (n = 5)	-0.3 ± 1.9	22.4 ± 0.8 (n = 5)
p.Arg536Gly	5.17 ± 1.33 (n = 15)	64.8 ± 7.8 **	460 ± 73	64.5 ± 4.3 (n = 22)	153 ± 16 (n = 10)	-66.7 ± 2.3	8.29 ± 1.12 (n = 12)	-1.7 ± 1.9	23.2 ± 2.2 (n = 8)
p.Asn578Ile	7.39 ± 2.08 * (n = 5)	70.8 ± 3.3 **	591 ± 26 *	60.6 ± 1.3 * (n = 5)	219 ± 25 (n = 5)	-69.4 ± 1.4 *	8.01 ± 0.90 (n = 4)	-4.1 ± 2.9	24.5 ± 0.5 (n = 4)

Table S4. Biophysical parameters for Kv4.1 wild-type and variant homotetrameric channels

Kv4.1 channels (grey, variant channels with red or purple dots) were expressed in the absence of auxiliary β-subunits. Mean values ± SD and number of observations (n) are given for the peak current amplitude at + 40 mV (I_{+40}), the time constants of macroscopic inactivation (τ_1 and τ_2) including the fractional amplitude of τ_1 (%), the time constant of recovery from inactivation (τ_{rec}) and the voltages of halfmaximal inactivation ($V_{1/2,inact}$) and activation ($V_{1/2,act}$) with corresponding slope factors (k_{inact} and k_{act}, respectively); Statistics are based on one-way analysis of variance (ANOVA) with Dunnett's post hoc testing. For significant variant effects (i.e., deviations from Kv4.1 WT; key variants: red, maternally inherited missense variants: purple) significance levels are indicated with * ($p<0.05$) or ** ($p<0.0001$); shaded fields: no significant differences found compared to Kv4.1 WT.

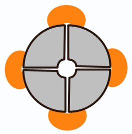

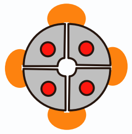

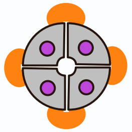

Heterologous expression:

Kv4.1 + KChIP	I_{+40} (μA)	τ_1 (ms)	τ_2 (ms)	% τ_1	τ_{rec} (ms)	$V_{1/2,inact}$ (mV)	k_{inact} (mV)	$V_{1/2,act}$ (mV)	k_{act} (mV)
WT	21.7 ± 6.0 (n = 7)	73.9 ± 8.1	176 ± 64	90.5 ± 6.6 (n = 6)	14.9 ± 3.5 (n = 5)	-48.0 ± 4.2	5.47 ± 1.28 (n = 5)	-7.0 ± 3.2	25.1 ± 2.1 (n = 6)
p.Thr57Met	24.4 ± 7.2 (n = 6)	52.1 ± 10.7 *	93.8 ± 19.4 *	55.8 ± 13.6 ** (n = 13)	20.4 ± 6.3 (n = 7)	-50.3 ± 2.0	5.82 ± 0.61 (n = 7)	-1.9 ± 3.4	20.8 ± 1.8 (n = 10)
p.Arg60Cys	23.3 ± 5.2 (n = 8)	74.7 ± 11.2	243 ± 93	93.2 ± 4.6 (n = 7)	11.0 ± 2.7 (n = 4)	-44.2 ± 1.6	6.76 ± 1.51 (n = 5)	-2.4 ± 2.8	26.9 ± 2.0 (n = 4)
p.Gly80Arg	15.7 ± 4.7 (n = 6)	59.3 ± 8.8	109 ± 15	49.6 ± 8.1 ** (n = 6)	23.1 ± 1.6 (n = 5)	-55.0 ± 3.2 *	4.44 ± 0.28 (n = 5)	-11.3 ± 2.8	20.1 ± 1.0 (n = 4)
p.Arg92Cys	3.50 ± 3.30 * (n = 11)	45.2 ± 4.8 **	110 ± 37 *	66.0 ± 16.1 * (n = 10)	27.3 ± 2.7 (n = 9)	-45.8 ± 2.6	6.69 ± 0.93 (n = 8)	+11.1 ± 10.0 **	24.7 ± 3.5 (n = 7)
p.Arg107Gln	29.0 ± 14.7 (n = 6)	59.1 ± 9.5	113 ± 26	79.4 ± 12.5 (n = 6)	13.9 ± 4.9 (n = 5)	-50.6 ± 1.7	4.94 ± 0.77 (n = 5)	-6.1 ± 1.3	24.5 ± 1.9 (n = 5)
p.Asp115Asn	11.6 ± 8.3 (n = 6)	55.5 ± 6.8 *	104 ± 12 *	69.0 ± 16.8 (n = 6)	23.0 ± 4.2 (n = 5)	-49.3 ± 2.8	8.32 ± 2.70 * (n = 6)	+3.1 ± 9.4 *	24.4 ± 4.1 (n = 6)
p.Arg146Cys	4.63 ± 2.32 * (n = 6)	45.7 ± 6.1 **	87.6 ± 7.3 *	39.2 ± 11.2 ** (n = 6)	24.3 ± 2.9 (n = 5)	-52.4 ± 1.3	6.48 ± 1.07 (n = 4)	-1.3 ± 2.4	21.3 ± 2.2 (n = 4)
p.Ala148Thr	17.5 ± 9.4 (n = 6)	70.3 ± 13.9	143 ± 44	77.1 ± 25.1 (n = 6)	19.4 ± 8.5 (n = 5)	-50.2 ± 4.1	4.59 ± 0.31 (n = 5)	-6.8 ± 6.2	21.0 ± 4.3 (n = 5)
p.Ala202Thr	57.9 ± 27.2 ** (n = 6)	54.7 ± 12.7 *	118 ± 49	66.9 ± 19.2 * (n = 9)	16.0 ± 2.9 (n = 8)	-48.4 ± 3.6	4.43 ± 0.42 (n = 8)	-1.9 ± 2.3	21.5 ± 2.0 (n = 9)
p.His308Tyr	12.5 ± 4.9 (n = 11)	70.8 ± 13.0	124 ± 16	41.4 ± 20.4 ** (n = 25)	1520 ± 540 ** (n = 11)	-88.3 ± 6.8 **	6.28 ± 3.34 (n = 9)	-30.5 ± 8.8 **	30.7 ± 7.7 * (n = 13)
p.Arg431Cys	43.3 ± 19.2 * (n = 8)	54.1 ± 8.6 *	124 ± 49	82.5 ± 18.4 (n = 8)	13.5 ± 1.5 (n = 5)	-47.2 ± 4.2	5.65 ± 0.85 (n = 5)	-1.8 ± 4.3	22.3 ± 0.7 (n = 3)
p.Lys450*	0.62 ± 0.27 * (n = 19)	81.9 ± 11.2	290 ± 37 **	70.1 ± 4.2 * (n = 20)	89.1 ± 17.2 (n = 10)	-60.4 ± 3.7 **	8.64 ± 1.42 * (n = 10)	+20.8 ± 6.9 **	36.6 ± 4.2 ** (n = 11)
p.Thr516Ser	38.5 ± 14.3 * (n = 13)	54.6 ± 5.6 *	202 ± 27	72.0 ± 2.6 (n = 13)	33.0 ± 6.7 (n = 8)	-49.7 ± 2.2	5.28 ± 0.56 (n = 5)	-5.5 ± 2.4	19.4 ± 1.7 (n = 5)
p.Arg536Gly	17.7 ± 9.5 (n = 6)	42.2 ± 5.4 **	72.1 ± 8.1 *	65.0 ± 16.5 * (n = 6)	13.7 ± 1.1 (n = 4)	-45.8 ± 0.6	5.37 ± 0.90 (n = 4)	+2.7 ± 1.7	21.2 ± 1.7 (n = 4)
p.Asn578Ile	24.4 ± 11.9 (n = 6)	62.6 ± 7.3	194 ± 117	87.4 ± 13.7 (n = 6)	20.6 ± 3.3 (n = 4)	-48.6 ± 3.9	6.68 ± 2.48 (n = 4)	-1.4 ± 2.6	24.5 ± 1.2 (n = 5)

Table S5. Biophysical parameters for Kv4.1 wild-type and variant binary channels with KChIP

Kv4.1 channels (grey, variant channels with red or purple dots) were studied in the presence of KChIP (orange). Mean values ± SD and number of observations (n) are given for the peak current amplitude at + 40 mV (I_{+40}), the time constants of macroscopic inactivation (τ_1 and τ_2) including the fractional amplitude of τ_1 (%), the time constant of recovery from inactivation (τ_{rec}) and the voltages of halfmaximal inactivation ($V_{1/2,inact}$) and activation ($V_{1/2,act}$) with corresponding slope factors (k_{inact} and k_{act}, respectively); Statistics are based on one-way analysis of variance (ANOVA) with Dunnett's post hoc testing. For significant variant effects (i.e., deviations from Kv4.1 WT + KChIP; key variants: red, maternally inherited missense variants: purple) significance levels are indicated with * (p<0.05) or ** (p<0.0001); shaded fields: no significant differences found compared to Kv4.1 WT + KChIP.

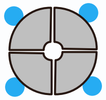

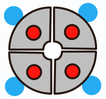

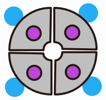

Heterologous expression:

Kv4.1 + DPP	I_{+40} (µA)	τ_1 (ms)	τ_2 (ms)	% τ_1	τ_{rec} (ms)	$V_{1/2,inact}$ (mV)	k_{inact} (mV)	$V_{1/2,act}$ (mV)	k_{act} (mV)
WT	62.8 ± 23.3 (n = 5)	26.3 ± 3.8	289 ± 59	71.7 ± 2.7 (n = 11)	29.7 ± 8.4 (n = 5)	-71.9 ± 3.9	8.46 ± 2.63 (n = 4)	-22.4 ± 4.8	24.7 ± 4.6 (n = 5)
p.Thr57Met	44.6 ± 11.2 (n = 6)	35.8 ± 2.9 **	279 ± 27	67.1 ± 2.5 * (n = 13)	45.8 ± 11.6 (n = 7)	-74.8 ± 1.2	7.03 ± 0.76 (n = 7)	-23.2 ± 3.3	21.9 ± 2.7 (n = 9)
p.Arg60Cys	70.9 ± 20.3 (n = 6)	21.5 ± 1.9	305 ± 15	75.8 ± 1.8 * (n = 11)	34.1 ± 3.3 (n = 4)	-73.4 ± 3.3	6.17 ± 0.77 (n = 5)	-24.2 ± 6.0	19.8 ± 5.9 (n = 6)
p.Gly80Arg	57.1 ± 17.9 (n = 6)	19.2 ± 2.0 *	322 ± 20	74.2 ± 3.0 (n = 11)	27.9 ± 6.4 (n = 5)	-73.8 ± 1.8	5.43 ± 0.66 (n = 5)	-28.9 ± 3.5	19.2 ± 3.6 (n = 5)
p.Arg92Cys	9.23 ± 3.65 ** (n = 13)	33.0 ± 4.2 *	330 ± 43	68.9 ± 3.3 (n = 12)	39.1 ± 4.8 (n = 9)	-67.3 ± 4.1	9.41 ± 1.79 (n = 9)	-1.5 ± 14.2 *	27.7 ± 4.4 (n = 8)
p.Arg107Gln	60.7 ± 26.6 (n = 6)	24.2 ± 4.6	252 ± 33	76.8 ± 3.1 ** (n = 18)	19.8 ± 1.9 (n = 4)	-57.9 ± 8.6 **	12.1 ± 2.9 * (n = 4)	-15.8 ± 7.7	25.3 ± 4.7 (n = 7)
p.Asp115Asn	48.2 ± 17.5 (n = 6)	23.4 ± 2.8	350 ± 12 *	77.2 ± 2.1 ** (n = 10)	28.3 ± 3.8 (n = 4)	-72.8 ± 4.3	10.0 ± 2.0 (n = 4)	-17.5 ± 12.6	27.7 ± 5.1 (n = 4)
p.Arg146Cys	18.2 ± 2.0 ** (n = 6)	17.9 ± 2.1 *	359 ± 41 **	85.9 ± 1.0 ** (n = 10)	26.1 ± 4.2 (n = 4)	-73.8 ± 1.3	7.45 ± 0.43 (n = 4)	-24.7 ± 4.6	18.1 ± 3.7 (n = 6)
p.Ala148Thr	38.5 ± 12.2 * (n = 6)	24.1 ± 3.9	372 ± 41 **	80.6 ± 3.0 ** (n = 10)	24.6 ± 4.9 (n = 4)	-73.2 ± 3.4	7.68 ± 0.14 (n = 4)	-24.4 ± 5.4	20.3 ± 5.2 (n = 6)
p.Ala202Thr	48.8 ± 8.6 (n = 6)	35.7 ± 5.3 **	313 ± 28	69.3 ± 2.2 (n = 16)	48.6 ± 6.6 (n = 8)	-72.9 ± 1.3	8.03 ± 0.50 (n = 8)	-17.9 ± 4.2	20.8 ± 3.2 (n = 10)
p.His308Tyr	0.82 ± 0.40 ** (n = 14)	47.6 ± 3.0 **	317 ± 50	64.5 ± 3.5 ** (n = 14)	2420 ± 1350 ** (n = 8)	-110.3 ± 6.5 **	6.74 ± 3.56 (n = 8)	-17.2 ± 14.3	30.0 ± 8.0 (n = 10)
p.Arg431Cys	65.7 ± 19.9 (n = 6)	17.8 ± 1.2 *	283 ± 32	81.4 ± 1.8 ** (n = 11)	20.6 ± 1.8 (n = 4)	-71.5 ± 2.0	7.68 ± 1.45 (n = 4)	-24.3 ± 5.3	20.6 ± 5.8 (n = 6)
p.Lys450*	1.27 ± 0.54 ** (n = 10)	41.9 ± 12.1 **	279 ± 36	71.6 ± 3.6 (n = 14)	75.7 ± 5.4 (n = 7)	-80.2 ± 2.5 *	7.97 ± 1.06 (n = 7)	-23.7 ± 7.5	25.6 ± 4.3 (n = 8)
p.Thr516Ser	47.8 ± 13.6 (n = 11)	33.9 ± 3.8 *	320 ± 23	67.4 ± 2.4 * (n = 11)	50.8 ± 6.4 (n = 6)	-70.2 ± 2.6	6.38 ± 0.23 (n = 8)	-16.6 ± 2.6	22.0 ± 2.0 (n = 7)
p.Arg536Gly	52.5 ± 10.1 (n = 4)	16.6 ± 1.8 *	272 ± 23	82.2 ± 1.8 ** (n = 9)	17.7 ± 1.9 (n = 4)	-70.0 ± 1.2	5.58 ± 0.52 (n = 4)	-25.7 ± 2.8	17.8 ± 1.5 (n = 4)
p.Asn578Ile	46.1 ± 6.0 (n = 4)	23.0 ± 3.8	332 ± 21 *	74.7 ± 2.8 (n = 10)	48.0 ± 8.3 (n = 3)	-78.1 ± 1.8	6.36 ± 0.76 (n = 4)	-31.7 ± 3.9	18.6 ± 5.3 (n = 5)

Table S6. Biophysical parameters for Kv4.1 wild-type and variant binary channels with DPP

Kv4.1 channels (grey, variant channels with red or purple dots) were studied in the presence of DPP (blue). Mean values ± SD and number of observations (n) are given for the peak current amplitude at + 40 mV (I_{+40}), the time constants of macroscopic inactivation (τ_1 and τ_2) including the fractional amplitude of τ_1 (%), the time constant of recovery from inactivation (τ_{rec}) and the voltages of halfmaximal inactivation ($V_{1/2,inact}$) and activation ($V_{1/2,act}$) with corresponding slope factors (k_{inact} and k_{act}, respectively); Statistics are based on one-way analysis of variance (ANOVA) with Dunnett's post hoc testing. For significant variant effects (i.e., deviations from Kv4.1 WT + DPP; key variants: red, maternally inherited missense variants: purple) significance levels are indicated with * ($p<0.05$) or ** ($p<0.0001$).

		Kv4.1 functional analysis																													
		peak current amplitude						macroscopic inactivation						recovery from inactivation						voltage dependence of steady-state inactivation						voltage dependence of activation					
Individual #		t	a	K	D	= ± K	= ± D	t	a	K	D	= ± K	= ± D	t	a	K	D	= ± K	= ± D	t	a	K	D	= ± K	= ± D	t	a	K	D	= ± D	PS3 score
	**DNVs**																														
1	c.274C>T (p.Arg92Cys)	Y	Y	Y	Y	Y	N	Y	Y	Y	Y	N	N	N	N	N	N	N	N	N	Y	N	N	N	N	Y	Y	Y	Y	Y	15
2	c.343G>A (p.Asp115Asn)	Y	N	N	N	Y	N	Y	Y	Y	Y	N	N	N	N	N	N	N	N	N	Y	Y	N	N	Y	N	Y	Y	N	Y	12
	**PTVs**																														
3	c.182_194del (p.Tyr61Cysfs*31)	complete LOF																													
4	c.295C>T (p.Arg99*)	complete LOF																													
5	c.1348A>T (p.Lys450*)	Y	Y	Y	Y	N	N	Y	Y	Y	Y	N	N	N	N	N	N	Y	N	Y	N	Y	Y	Y	N	N	Y	Y	N	N	15
	**maternally inherited missense variants**																														
	variants affecting the N-terminal cytoplasmic domain																														
6	c.170C>T (p.Thr57Met)	N	N	N	N	N	N	N	Y	Y	Y	N	N	N	N	N	N	N	N	N	Y	N	N	N	N	N	Y	N	N	N	5
7	c.178C>T (p.Arg60Cys)	N	N	N	N	N	N	N	N	N	Y	N	N	N	N	N	N	N	N	N	N	N	N	N	N	N	N	N	N	N	1
8	c.238G>C (p.Gly80Arg)	N	Y	N	N	N	N	N	Y	Y	Y	N	N	N	N	N	N	N	N	N	Y	Y	N	N	N	N	N	N	N	N	6
9	c.320G>A (p.Arg107Gln)	N	N	N	N	N	N	Y	N	N	Y	N	N	N	N	N	N	N	N	N	N	N	Y	N	Y	N	N	N	N	N	4
10	c.436C>T (p.Arg146Cys)	Y	N	Y	Y	Y	N	Y	Y	Y	Y	N	N	N	N	N	N	N	N	N	Y	N	N	N	N	N	Y	N	N	N	10
11	c.442G>A (p.Ala148Thr)	N	N	N	Y	N	N	Y	Y	N	Y	N	N	N	N	N	N	N	N	N	Y	N	N	N	Y	N	N	N	N	N	6
	variants affecting transmembrane domains																														
12	c.604G>A (p.Ala202Thr)	N	Y	Y	N	N	N	N	Y	Y	Y	N	N	N	N	N	N	N	N	Y	Y	N	N	N	N	Y	N	N	N	N	8
13	c.922C>T (p.His308Tyr)	Y	Y	N	Y	N	Y	Y	Y	Y	Y	N	Y	Y	Y	Y	Y	N	Y	Y	Y	Y	Y	N	N	Y	Y	Y	N	Y	22
	variants affecting the C-terminal cytoplasmic domain																														
14	c.1291C>T (p.Arg431Cys)	N	Y	Y	N	N	N	N	N	Y	Y	N	N	N	N	N	N	N	N	N	Y	N	N	N	Y	N	N	N	N	N	6
15 & 16	c.1546A>T (p.Thr516Ser)	N	Y	Y	N	N	N	Y	N	Y	Y	N	N	N	N	N	N	N	N	Y	Y	N	N	N	N	Y	N	N	N	N	8
17	c.1606A>G (p.Arg536Gly)	N	N	N	N	N	N	N	Y	Y	Y	N	N	N	N	N	N	N	N	N	N	N	N	N	N	N	N	N	N	N	3
18	c.1733A>T (p.Asn578Ile)	N	Y	N	N	N	N	Y	Y	N	Y	N	N	N	N	N	N	N	N	N	Y	N	N	N	N	N	N	N	N	N	5

Table S7. PS3 scoring for functionally expressed *KCND1* variants

Functional *KCND1* variants were assessed based on significant alterations relative to Kv4.1 WT. For each biophysical parameter and in all channel configurations (t: ternary, a: alone, K: only with KChIP, D: only with DPP) the following questions were asked: 1) Is there a significant difference relative to Kv4.1 WT? Yes (Y) or No (N); 2) Is the β-subunit effect on this biophysical parameter absent (i.e.; no KChIP effect, = ± K or no DPP effect, = ± D)? Yes (Y) or No (N). Assessment based on the data from Tables S3 - S6; see also Figures S3 - S5.

Supplemental References

1. Kise, Y., Kasuya, G., Okamoto, H.H., Yamanouchi, D., Kobayashi, K., Kusakizako, T., Nishizawa, T., Nakajo, K., and Nureki, O. (2021). Structural basis of gating modulation of Kv4 channel complexes. Nature *599*, 158-164.

2. An, W.F., Bowlby, M.R., Betty, M., Cao, J., Ling, H.P., Mendoza, G., Hinson, J.W., Mattsson, K.I., Strassle, B.W., Trimmer, J.S., and Rhodes, K.J. (2000). Modulation of A-type potassium channels by a family of calcium sensors. Nature *403*, 553-556.

3. Nadal, M.S., Ozaita, A., Amarillo, Y., de Miera, E.V., Ma, Y., Mo, W., Goldberg, E.M., Misumi, Y., Ikehara, Y., Neubert, T.A., and Rudy, B. (2003). The CD26-related dipeptidyl aminopeptidase-like protein DPPX is a critical component of neuronal A-Type K^+ channels. Neuron *37*, 449-461.
