# Supplementary material for: Etiological involvement of KCND1 variants in an X-linked neurodevelopmental disorder with variable expressivity
Source: Am J Hum Genet. 2024 May 20;111(6):1206–21. doi: 10.1016/j.ajhg.2024.04.019 (PMC11179411; doi:10.1016/j.ajhg.2024.04.019)
Supplement: Document S2. Article plus supplemental information [file mmc3.pdf]

Etiological involvement of *KCND1* variants in an X-linked neurodevelopmental disorder with variable expressivity

Authors

Tassja Kalm, Claudia Schob, Hanna Völler, ...,
Christian Kubisch, Robert Bähring, Stefan Kindler

Correspondence

r.baehring@uke.uni-hamburg.de (R.B.),
kindler@uke.de (S.K.)

Hemizygous variants of *KCND1* lead to alterations in the biophysical properties of the encoded ion channel, implicating dysfunction of Kv4.1 voltage-gated potassium channels in the etiology of an X-linked neurodevelopmental disorder with variable expressivity.

Kalm et al., 2024, The American Journal of Human Genetics *111*, 1206–1221
June 6, 2024 © 2024 The Authors.
https://doi.org/10.1016/j.ajhg.2024.04.019

ARTICLE

Etiological involvement of *KCND1* variants in an X-linked neurodevelopmental disorder with variable expressivity

Tassja Kalm,[1] Claudia Schob,[2] Hanna Völler,[2] Thatjana Gardeitchik,[3] Christian Gilissen,[3] Rolph Pfundt,[3] Chiara Klöckner,[4] Konrad Platzer,[4] Annick Klabunde-Cherwon,[5] Markus Ries,[5] Steffen Syrbe,[5] Francesca Beccaria,[6] Francesca Madia,[7] Marcello Scala,[7,8] Federico Zara,[7,8] Floris Hofstede,[9] Marleen E.H. Simon,[10] Richard H. van Jaarsveld,[10] Renske Oegema,[10] Koen L.I. van Gassen,[10] Sjoerd J.B. Holwerda,[10] Tahsin Stefan Barakat,[11,12,13] Arjan Bouman,[11]

(Author list continued on next page)

Summary

Utilizing trio whole-exome sequencing and a gene matching approach, we identified a cohort of 18 male individuals from 17 families with hemizygous variants in *KCND1*, including two *de novo* missense variants, three maternally inherited protein-truncating variants, and 12 maternally inherited missense variants. Affected subjects present with a neurodevelopmental disorder characterized by diverse neurological abnormalities, mostly delays in different developmental domains, but also distinct neuropsychiatric signs and epilepsy. Heterozygous carrier mothers are clinically unaffected. *KCND1* encodes the α-subunit of Kv4.1 voltage-gated potassium channels. All variant-associated amino acid substitutions affect either the cytoplasmic N- or C-terminus of the channel protein except for two occurring in transmembrane segments 1 and 4. Kv4.1 channels were functionally characterized in the absence and presence of auxiliary β subunits. Variant-specific alterations of biophysical channel properties were diverse and varied in magnitude. Genetic data analysis in combination with our functional assessment shows that Kv4.1 channel dysfunction is involved in the pathogenesis of an X-linked neurodevelopmental disorder frequently associated with a variable neuropsychiatric clinical phenotype.

Introduction

Voltage-dependent potassium (Kv) channels represent a diverse group of ion channels encoded by a gene superfamily that includes twelve members (Kv1–12) and about 40 different genes.[1] The particular physiological role of each Kv channel subtype is determined not only by its cellular abundance and subcellular distribution but also by its specific voltage dependence and gating kinetics.[1] The three members of the Kv4 subfamily, encoded by the genes *KCND1–3*, mediate a subthreshold-activating, somatodendritic, rapidly activating and inactivating (A-type) potassium current (I_{SA}) in neurons.[2] I_{SA} plays a critical role in distinct aspects of neurophysiology, including the control of low frequency repetitive discharge, regulation of dendritic excitation, and action potential backpropagation, as well as synaptic plasticity.[2–4]

Like all members of the Kv channel superfamily, Kv4 α-subunits consist of six transmembrane segments (S1–S6), flanked by cytoplasmic termini. A re-entrant pore loop between S5 and S6 contains the highly conserved potassium channel "signature sequence," which mediates ion selectivity (Figure 1).[5] The α-subunits tetramerize to constitute functional channels. Characteristic for Kv1–4 subfamily members, the cytoplasmic N-terminus contains a tetramerization (T1) domain, which mediates subfamily-specific assembly, with a critical involvement of specific Zn^{2+} coordination sites in subfamilies Kv2–4.[6] Within a

[1]Institute for Cellular and Integrative Physiology, Center for Experimental Medicine, University Medical Center Hamburg-Eppendorf, 20246 Hamburg, Germany; [2]Institute of Human Genetics, University Medical Center Hamburg-Eppendorf, 20246 Hamburg, Germany; [3]Department of Human Genetics, Radboud University Medical Center, Nijmegen 6525 GA, the Netherlands; [4]Institute of Human Genetics, University of Leipzig Medical Center, 04103 Leipzig, Germany; [5]Division of Pediatric Epileptology, Centre for Paediatric and Adolescent Medicine, University Hospital Heidelberg, 69120 Heidelberg, Germany; [6]Epilepsy Center, Department of Child Neuropsychiatry, Territorial Social-Health Agency, 46100 Mantova, Italy; [7]Medical Genetics Unit, IRCCS Istituto Giannina Gaslini, 16147 Genoa, Italy; [8]Department of Neurosciences, Rehabilitation, Ophthalmology, Genetics, Maternal and Child Health, University of Genoa, 16145 Genoa, Italy; [9]Department of General Pediatrics, Wilhelmina Children's Hospital, University Medical Centre Utrecht, Utrecht, the Netherlands; [10]Department of Clinical Genetics, University Medical Center Utrecht, Utrecht 3584 EA, the Netherlands; [11]Department of Clinical Genetics, Erasmus MC University Medical Center, Rotterdam 3000 CA, the Netherlands; [12]ENCORE Expertise Center for Neurodevelopmental Disorders, Erasmus MC University Medical Center, Rotterdam 3000 CA, the Netherlands; [13]Discovery Unit, Department of Clinical Genetics, Erasmus MC University Medical Center, Rotterdam 3000 CA, the Netherlands; [14]Genomics and Medicine, NIMGenetics, 28108 Madrid, Spain; [15]Pediatric Neurology Department, Quironsalud University Hospital Madrid, School of Medicine, European University of Madrid, 28224 Madrid, Spain; [16]Genomics, Genologica Medica, 29016 Málaga, Spain; [17]Division of Medical Genetics, Department of Specialized Medicine, Montreal Children's Hospital, McGill University Health Centre, QC H4A 3J1 Montreal, Canada; [18]Department of Human Genetics, McGill University, QC H4A 3J1 Montreal, Canada; [19]Child Neuropsychiatry Unit, IRCCS Istituto Giannina Gaslini, 16147 Genoa, Italy; [20]Pediatric Neurology and Neuromuscular Diseases Unit, IRCCS Istituto Giannina Gaslini, 16147 Genoa,

(Affiliations continued on next page)

© 2024 The Authors. This is an open access article under the CC BY license (http://creativecommons.org/licenses/by/4.0/).

Marjon van Slegtenhorst,[11] Sara Álvarez,[14] Alberto Fernández-Jaén,[15] Javier Porta,[16] Andrea Accogli,[17,18] Margherita Maria Mancardi,[19] Pasquale Striano,[8,20] Michele Iacomino,[7] Jong-Hee Chae,[21,22] SeSong Jang,[21] Soo Y. Kim,[22] David Chitayat,[23,24] Saadet Mercimek-Andrews,[24,25] Christel Depienne,[26] Antje Kampmeier,[26] Alma Kuechler,[26] Harald Surowy,[26] Enrico Silvio Bertini,[27] Francesca Clementina Radio,[28] Cecilia Mancini,[28] Simone Pizzi,[28] Marco Tartaglia,[28] Lucas Gauthier,[29] David Genevieve,[30,31] Mylène Tharreau,[29] Noy Azoulay,[32,33] Gal Zaks-Hoffer,[33,34] Nesia K. Gilad,[34,35] Naama Orenstein,[34,35] Geneviève Bernard,[17,36,37] Isabelle Thiffault,[38,39,40] Jonas Denecke,[41] Theresia Herget,[2] Fanny Kortüm,[2] Christian Kubisch,[2] Robert Bähring,[1,42,*] and Stefan Kindler[2,42,*]

tetramer, S5 and S6 surround the central ion conduction pathway with the intertwined and flexible distal S6 segments acting as the cytoplasmic gate, while the peripheral S1–S4 segments (especially the positively charged amino acid residues in S4) act as voltage sensors. Kv channel opening and closing is mediated by direct mechanical coupling of the motile voltage sensors to the cytoplasmic S6 gate via the S4-S5 linkers.[7]

Kv4 channels form complexes with auxiliary β subunits, namely cytosolic Kv channel interacting proteins (KChIPs)[13] and/or transmembrane dipeptidyl aminopeptidase-like proteins (DPPs).[14] In heterologous cell systems, both KChIPs and DPPs increase Kv4 channel surface levels and modulate channel gating in a β-subunit-specific manner (see Figures S3–S5).[13–19] Structural and functional analyses revealed that both the cytoplasmic N-terminus, including the T1-domain, and the cytoplasmic C-terminus of Kv4 α-subunits interact with KChIPs, while S1 and S2 are involved in DPP binding.[15,19–23] It is assumed that native Kv4 channels assemble in a ternary fashion, i.e., with both types of auxiliary β subunits (KChIPs and DPPs, see Figure S1 and Video S1).[21,23,24] Since KChIP and DPP interaction sites on Kv4 α-subunits do not overlap, effects of both β subunits on Kv4 channels are expected to be more or less additive.[25] These β-subunit-mediated adaptations of channel physiology need to be considered, and the ability to interact with the β subunits needs to be tested when Kv4 channel function is to be evaluated, and a rigorous testing of putative variant pathogenicity is contemplated.

Variants in several Kv channel genes are associated with monogenic types of epilepsy or developmental and epileptic encephalopathy.[26] In particular, a number of heterozygous missense variants in *KCND2* (MIM: 605410) that alter Kv4.2 channel gating have been linked to early-onset global developmental delay of distinct severity, often in combination with seizures, muscular hypotonia, and/or visual impairment.[8–10,27,28] Also, a hemizygous nonsense variant of X-chromosomal *KCND1* (MIM: 300281) has been suggested as a candidate pathogenic variant in a single individual with focal epilepsy, however, with no reference to Kv4.1 channel function.[29]

Here, we report on a cohort of 18 males with 17 distinct hemizygous *KCND1* variants, including two *de novo* missense variants (DNVs), three maternally inherited protein-truncating variants (PTVs), and 12 maternally inherited missense variants. Our genetic and functional analyses support the etiological involvement of Kv4.1 channel dysfunction in an X-linked neurodevelopmental disorder frequently associated with a variable neuropsychiatric clinical phenotype.

Material and methods

Recruitment of probands and genetic analysis

Utilizing GeneMatcher,[30] 18 individuals from 17 families were gathered as part of an international collaborative project. In most cases, affected probands were initially examined by their referring physicians, genetic analyses were performed in a diagnostic setting, and genetic findings were subsequently used for secondary research. In all probands, *KCND1* variants were independently identified at the referring medical centers as the most-likely disease-causing variant. Biological samples were obtained after written informed consent was given by the affected individuals or their legal guardians. Informed consent for the publication of clinical and genetic data was obtained from all participants. The

Italy; [21]Department of Pediatrics, Seoul National University College of Medicine, Seoul 110-744, Republic of Korea; [22]Department of Genomic Medicine, Rare Disease Center, Seoul National University Hospital, Seoul 03080, Republic of Korea; [23]The Prenatal Diagnosis and Medical Genetics Program, Department of Obstetrics and Gynecology, Mount Sinai Hospital, University of Toronto ON M5G 1E2 Toronto, Canada; [24]Division of Clinical and Metabolic Genetics, Department of Pediatrics, The Hospital for SickKids, University of Toronto, M5G 1X8 Toronto, Canada; [25]Department of Medical Genetics, Faculty of Medicine and Dentistry, University of Alberta, AB T6G 2H7 Edmonton, Canada; [26]Institute of Human Genetics, University Hospital Essen, University Duisburg-Essen, 45122 Essen, Germany; [27]Neuromuscular Disorders, Bambino Gesù Children's Hospital, IRCCS, 00146 Rome, Italy; [28]Molecular Genetics and Functional Genomics, Bambino Gesù Children's Hospital, IRCCS, 00146 Rome, Italy; [29]Department of Molecular Genetics and Cytogenomics, Rare and Autoinflammatory Diseases Unit, University Hospital of Montpellier, 34295 Montpellier, France; [30]Montpellier University, Inserm U1183, Montpellier, France; [31]Department of Clinical Genetics, University Hospital of Montpellier, 34295 Montpellier, France; [32]The Genetic Institute of Maccabi Health Services, Rehovot 7610000, Israel; [33]Raphael Recanati Genetics Institute, Beilinson Hospital, Rabin Medical Center, Petach Tikva 49100, Israel; [34]Sackler Faculty of Medicine, Tel Aviv University, Tel Aviv 69978, Israel; [35]Pediatric Genetics Unit, Schneider Children's Medical Center of Israel, Petah Tikvah 4920235, Israel; [36]Departments of Neurology and Neurosurgery, Pediatrics and Human Genetics, McGill University, Montreal, Canada; [37]Child Health and Human Development Program, Research Institute of the McGill University Health Centre, Montreal, Canada; [38]Genomic Medicine Center, Department of Pediatrics, Children's Mercy Kansas City, Kansas City, MO, USA; [39]UKMC School of Medicine, University of Missouri Kansas City, Kansas City, MO, USA; [40]Department of Pathology and Laboratory Medicine, Children's Mercy Kansas City, Kansas City, MO, USA; [41]Department of Pediatrics, University Medical Center Hamburg-Eppendorf, 20246 Hamburg, Germany
[42]These authors contributed equally
*Correspondence: r.baehring@uke.uni-hamburg.de (R.B.), kindler@uke.de (S.K.)
https://doi.org/10.1016/j.ajhg.2024.04.019.

study was performed in accordance with the Declaration of Helsinki protocols and approved by the ethics committees of the respective institutions. Trio whole-exome sequencing (WES) was performed in all subjects, and identified *KCND1* variants were confirmed by Sanger sequencing. The different medical centers involved in this study utilized comparable protocols for DNA isolation, WES, bioinformatic processing, and variant interpretation. Functional impact of identified putative pathogenic variants was predicted via the MetaDome webserver,[31] the AlphaMissense tool (https://alphamissense.hegelab.org/), the Rare Exome Variant Ensemble Learner (REVEL) algorithm (https://genome.ucsc.edu/cgi-bin/hgTrackUi?db=hg19&g=revel), the Combined Annotation Dependent Depletion (CADD) tool,[32] and the Polymorphism Phenotyping v2 (PolyPhen-2) tool.[33]

Plasmids and synthesis of Kv4.1 channels in *Xenopus* oocytes

The human *KCND1* cDNA (GenBank: NM_004979.6) encoding full-length Kv4.1 (GenBank: NP_004970.3, UniProtKB Q9NSA2)[34] was inserted into pGEM-HE to generate pGEM-OK-hKCND1, which carries an optimized Kozak sequence. Variant-specific mutations were introduced utilizing the Quick-Change II site-directed mutagenesis kit (Agilent), and resulting constructs were verified by Sanger sequencing. Similar to a previous study, pGEM vectors encoding human KChIP2b[15] and DPP6s (a generous gift from Nicole Schmitt, University of Copenhagen), two well-characterized isoforms present in many brain regions, and hereafter referred to as KChIP and DPP, respectively, were used for auxiliary β-subunit synthesis.[10,35] Plasmid linearization, *in vitro* transcription, and cRNA injection in *Xenopus laevis* oocytes were done as previously described.[10] All experimental procedures were in accordance with the national guidelines for the care and use of research animals and were approved by the local authorities. To generate Kv4.1 homotetramers, 5 ng total cRNA were injected per oocyte. Kv4.1 wild type (WT) and variant cRNAs were also co-injected KChIP or DPP cRNAs (5 + 5 ng per oocyte) for binary complex formation (only one type of auxiliary β-subunit) or with both KChIP and DPP cRNAs (2 + 5 + 5 ng per oocyte) for ternary complex formation.

Electrophysiology

Oocytes were used for electrophysiological recordings 1–3 days after cRNA injection. Currents were recorded at room temperature

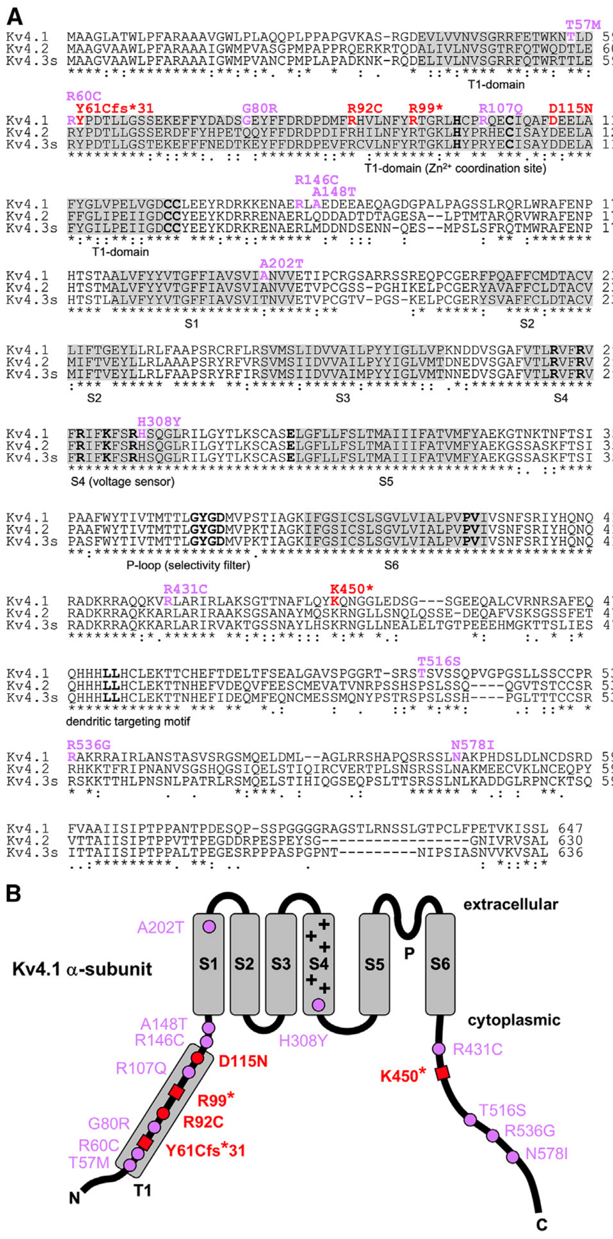

Figure 1. ***KCND1* variant-associated amino acid substitutions**
(A) Alignment of human Kv4 subfamily members Kv4.1, Kv4.2, and Kv4.3s (short isoform). Kv4.1 amino acid residues affected by the reported *KCND1* variants are shown in color, and respective changes are indicated above using single-letter code (red: initially studied *KCND1* group 1 variants; purple: additional maternally inherited missense variants, group 2). Regions highlighted in gray indicate the tetramerization (T1) domain and transmembrane segments S1–S6, as indicated below. Sequence motifs critical for channel assembly trafficking and function are indicated by bold letters ($HX_5CX_{20}CC$ motif in the T1-domain: Zn^{2+} coordination site;[6] positively charged residues in S4: voltage sensor; [7] potassium channel signature sequence GYGD in the pore loop [P]: selectivity filter;[5] critical dynamic coupling residues, glutamate [E] in the S4-S5 linker, and proline-valine [PV] in the distal S6 segment: operation of the cytoplasmic gate;[8,9,10,11] C-terminal di-leucine motif: dendritic targeting[12]). Numbers on the right specify amino acid residue positions. Sequences were aligned using Clustal Omega software (http://www.clustal.org/omega/). Residues that are perfectly conserved (*) or exhibit either strong or weak similarity (: or . for a scoring of >0.5 or ≤ 0.5, respectively, in the Gonnet PAM 250 matrix) are indicated. Note that Kv4.1 amino acid residues in positions 57, 60, 61, 92, 99, 107, 115, 146, 308, 431, 450, 536, and 578 are fully conserved in all three Kv4 subfamily members.
(B) Topology scheme of the Kv4.1 α-subunit (extracellular and cytoplasmic side indicated) with six transmembrane segments (S1–S6, gray boxes) and cytoplasmic N- and C-termini. Positively charged amino acid residues in S4 (+) mediate voltage sensing,[7] and a re-entrant P loop between S5 and S6 harbors the selectivity filter sequence.[5] The T1 domain (gray box) is located in the cytoplasmic N-terminus. Changes in amino acid sequence are indicated in single-letter code. The two amino acid substitutions and three truncation sites, respectively, associated with five *KCND1* group 1 variants are depicted as red dots (DNVs R92C and D115N) and red squares (PTVs Y61Cfs*31, R99*, and K450*). All other amino acid substitutions (12 maternally inherited missense variants) are depicted as purple dots. Note that all variant-associated amino acid substitutions, except for p.Ala202Thr (A202T in S1) and p.His308Tyr (H308Y in S4), reside within either the cytoplasmic N- or the cytoplasmic C-terminal domain of the protein and do not affect one of the indicated critical sequence motifs.

(20°C–22°C) under two-electrode voltage clamp as described[10] in a low chloride (15 mM) bath solution containing (in mM) 7.4 NaCl, 88.6 Na-aspartate, 2 KCl, 1.8 $CaCl_2$, 1 $MgCl_2$, and 5 HEPES, pH 7.4 with NaOH. Pulse protocols are explained in the figure legends. Data were analyzed using FitMaster (HEKA) and Kaleidagraph (Synergy Software), as described previously.[10,11,36] Current decay kinetics were approximated with a double-exponential function and the kinetics of recovery from inactivation with a single-exponential function. The voltage dependences of activation and steady-state inactivation were analyzed with appropriate Boltzmann functions.[10,11,36] Pooled data are presented as mean ± SD, unless stated otherwise. Statistical analyses for multiple groups are based on ANOVA with Dunnett's post hoc testing and for two groups on Student's t tests.

Results

Identification of hemizygous *KCND1* variants associated with variable neurological phenotypes

Utilizing trio WES and GeneMatcher, we identified distinct hemizygous coding variants of *KCND1* in a cohort of 18 male subjects from 17 families. The variant group includes one frameshift, two nonsense, and 12 missense mutations, all of which are maternally inherited, as well as two DNVs (Table 1). Affected individuals display diverse neurological abnormalities, primarily including delays in various developmental domains, distinct neuropsychiatric signs, and seizures (Tables 2 and S1). In each case, trio WES data analysis revealed the respective hemizygous *KCND1* variant as the most-likely genetic cause of the observed clinical phenotype, as no other (likely) pathogenic variants that could explain the observed clinical features were identified. *KCND1* is located on the X chromosome and encodes the Kv4.1 Kv channel α subunit.[34] Although Kv4 channels play a crucial role in brain function,[2] variants in *KCND1* have not been associated with a human disease phenotype in OMIM, though a single individual with focal epilepsy has been reported as hemizygous for a truncating variant in this gene.[29] Thus, the two central aims of the present work were to (1) functionally characterize the identified *KCND1* variants, thereby allowing their classification beyond uncertain clinical significance and (2) provide an overview of the phenotypic spectrum associated with hemizygous *KCND1* variants.

Initially, we focused on a set of five variants (group 1) for which the genetic data provide the strongest evidence for pathogenicity, i.e., DNVs and PTVs. The allele frequency in hemizygotes in the gnomAD database (v4.0.0, GRCh38/hg38)[37] is very low for the DNV c.274C>T (p.Arg92Cys) (7.54e-6, individual 1) and low for the DNV c.343G>A (p.Asp115Asn) (1.26e-5, individual 2) (Table 1). Both affected amino acid residues in the T1-domain are completely conserved throughout the Kv4 subfamily (Figure 1A). While well-established prediction tools support the pathogenicity of p.Arg92Cys, they provide ambiguous results for p.Asp115Asn (Table 1). We also identified three PTVs, namely c.182_194del (p.Tyr61Cysfs*31) (individual 3), c.295C>T (p.Arg99*) (individual 4), and c.1348A>T (p.Lys450*) (individual 5). While the former is not listed in gnomAD, p.Arg99* and p.Lys450* are both found at very low allele frequencies in hemizygotes (around 5.0e-6). High-scaled C-scores predicted by CADD suggest that the latter two are pathogenic variants. All PTVs are predicted to lead to nonsense-mediated decay (NMD) of the respective transcripts, thus resulting in a complete loss-of-function (LOF) in hemizygous males. However, as NMD is known to exhibit a large variability in its efficiency across mRNAs, cell types, tissues, and individuals, we also considered the possibility of NMD escape.[38] In case of NMD escape and synthesis of p.Tyr61Cysfs*31 and p.Arg99*, both truncated proteins are expected to remain cytosolic and be unable to form functional channels due to the lack of any transmembrane domains (Figure 1), thus still leading to complete LOF. In contrast, if synthesized, p.Lys450* contains transmembrane regions S1–S6 (Figure 1) and is hence theoretically able to form functional channels. Thus, from the five group 1 variants, p.Arg92Cys, p.Asp115Asn, and p.Lys450* were subsequently chosen for functional characterization, while cytosolic p.Tyr61Cysfs*31 and p.Arg99*, although patho-physiologically relevant, were not included in these experiments.

KCND1 group 1 variants: Functional characterization and associated clinical features

First of all, Kv4.1 WT and variant channels were functionally characterized in a ternary configuration, i.e., in the presence of both types of auxiliary β subunits (KChIP and DPP, Figures 2A and S1 and Video S1), a likely channel configuration in native neuronal tissues[24] (see material and methods). For the ternary Kv4.1 channel complexes, the following biophysical parameters were examined under two-electrode voltage clamp in *Xenopus* oocytes (see material and methods): peak current amplitude, macroscopic inactivation kinetics (i.e., kinetics of current decay), kinetics of recovery from inactivation, and voltage dependences of activation and steady-state inactivation.

Both Kv4.1 WT and variant ternary channels, even the truncated protein p.Lys450*, mediated rapidly, activating and inactivating currents with variable amplitudes and decay kinetics in response to depolarizing voltage steps (Figure 2B). Mean peak current amplitudes at a test voltage of +40 mV (19.2 ± 9.52 μA for Kv4.1 WT, $n = 16$) were found to be reduced for all three variants (Figure 2C). Macroscopic inactivation kinetics ($\tau_1 = 30.7 \pm 4.72$ ms, relative amplitude = 83 ± 11% and $\tau_2 = 85.8 \pm 56.8$ ms for Kv4.1 WT, $n = 21$) were accelerated for p.Asp115Asn, slowed for p.Arg92Cys and p.Lys450*, and the relative amplitude accounted for by τ_1 was significantly decreased for p.Lys450* (Figures 2D and 2F). The kinetics of recovery from inactivation ($\tau_{rec} = 11.2 \pm 2.02$ ms for Kv4.1 WT, $n = 16$), were unaffected for p.Arg92Cys and p.Asp115Asn but slowed for p.Lys450* (Figures 2E and 2G). Finally, we examined the voltage dependences of activation and steady-state inactivation (Figure 3A). For Kv4.1 WT,

Table 1. Description and *in silico* analysis of *KCND1* variants

Genetic analysis											Bioinformatic analysis				
			gnomAD v2.1.1				gnomAD v4.0.0								
Individual	Transcript GenBank: NM_004979.6	Protein GenBank: NP_004970.3	Allele count	# of hemizygotes	Allele frequency	Allele frequency in hemizygotes	Allele count	# of hemizygotes	Allele frequency	Allele frequency in hemizygotes	MetaDome	REVEL	CADD	AlphaMissense	PolyPhen-2
***De novo* missense variants**															
1	c.274C>T	p.Arg92Cys[a]	0	0	0	0	11	3	9.09e-6	7.54e-6	1.31	0.404	24.7	0	1.0
2	c.343G>A	p.Asp115Asn[a]	1	0	5.58e-6	0	18	5	1.49e-5	1.26e-5	0.53	0.165	23.6	0.037	0.388
**Maternally inherited truncating variants**															
3	c.182_194del	p.Tyr61Cysfs*31[a]	0	0	0	0	0	0	0	0	N/A	N/A	N/A	N/A	N/A
4	c.295C>T	p.Arg99*[a]	2	0	1.1e-5	0	6	2	4.96e-6	5.03e-6	N/A	N/A	35	N/A	N/A
5	c.1348A>T	p.Lys450*[a]	1	0	5.51e-6	0	5	1	8.87e-6	4.99e-6	N/A	N/A	37	N/A	N/A
**Maternally inherited missense variants**															
**Variants affecting the N-terminal cytoplasmic domain**															
6	c.170C>T	p.Thr57Met[a]	3	0	1.67e-5	0	12	0	9.92e-6	0	0.14	0.858	25.3	0.909	0.986
7	c.178C>T	p.Arg60Cys[a]	0	0	0	0	2	0	1.82e-6	0	0.16	0.469	29.0	0.534	1.0
8	c.238G>C	p.Gly80Arg	0	0	0	0	0	0	0	0	0.36	0.101	18.14	0.145	0.001
9	c.320G>A	p.Arg107Gln[a]	0	0	0	0	6	2	4.96e-6	5.03e-6	0.55	0.395	23.1	0.225	0.956
10	c.436C>T	p.Arg146Cys[a]	1	0	5.71e-6	0	3	2	2.74e-6	8.78e-6	0.18	0.807	26.5	0.952	1.0
11	c.442G>A	p.Ala148Thr	0	0	0	0	1	0	2.2e-6	0	0.23	0.438	20.7	0.112	0.471
**Variants affecting transmembrane domains**															
12	c.604G>A	p.Ala202Thr	5	2	2.53e-5	2.83e-5	130	35	1.08e-4	8.82e-5	0.74	0.613	22.7	0.207	0.236
13	c.922C>T	p.His308Tyr[a]	0	0	0	0	0	0	0	0	0.69	0.774	25.3	0.970	0.786
**Variants affecting the C-terminal cytoplasmic domain**															
14	c.1291C>T	p.Arg431Cys[a]	2	1	1.11e-5	1.56e-5	5	3	4.58e-6	8.38e-6	1.79	0.694	33	0.936	1.0
15/16	c.1546A>T	p.Thr516Ser	11	3	5.81e-5	4.63e-5	277	79	2.3e-4	2.01e-4	0.9	0.157	15.87	0.102	0.044
17	c.1606A>G	p.Arg536Gly[a]	3	2	1.68e-5	3.11e-5	32	14	2.65e-5	3.52e-5	0.46	0.822	25.1	0.194	0.949
18	c.1733A>T	p.Asn578Ile[a]	0	0	0	0	0	0	0	0	0.55	0.814	26.9	0.756	1.0

Individuals 15 and 16 are brothers of different age. Allele count, number of identified hemizygotes, and allele frequency of individual variants are derived from the gnomAD database (https://gnomad.broadinstitute.org/, gnomAD v2.1.1, GRCh37/hg19 and gnomAD v4.0.0, GRCh38/hg38; as of January 22, 2024). MetaDome tolerance scores are calculated as described (https://stuart.radboudumc.nl/metadome/). The phred-like scaled C score predicted by CADD (https://wintervar.wglab.org/) ranges from 1 to 99 with higher values indicating more deleterious variants. The AlphaMissense score (https://alphamissense.hegelab.org/) ranges from 0.0 to 1.0 with scores below 0.34 considered likely benign and scores higher than 0.564 being considered likely pathogenic. The REVEL and the PolyPhen-2 scores range from 0.0 (tolerated) to 1.0 (deleterious). CADD, Combined Annotation Dependent Depletion; HGVS, Human Genome Variation Society; N/A, not applicable.

[a]Respective native amino acids are conserved throughout the Kv4 family.

Table 2. Clinical features of subjects with hemizygous *de novo* missense or maternally inherited truncating *KCND1* group 1 variants

Individual #	1	2	3	4	5
cDNA variant GenBank: NM_004979.6	c.274C>T	c.343G>A	c.182_194del	c.295C>T	c.1348A>T
Protein variant GenBank: NP_004970.3	p.Arg92Cys	p.Asp115Asn	p.Tyr61Cysfs*31	p.Arg99*	p.Lys450*
Variant origin	*de novo*	*de novo*	maternally inherited	maternally inherited	maternally inherited
Age at last examination	15 years	9 years 11 months	16 years	16 years	3 years 2 months
Age at first signs	10 years	6 years	2 years 3 months	abt 2 years	neonatal period
First signs	anxiety, tics	speech delay, seizures	seizures	speech delay, motor delay, anxiety	speech delay, motor delay
Motor development	normal	normal	normal	delayed	delayed
Intellectual development	average	low average, TIQ 85	average	borderline mental functioning – low average, TIQ 74-86	extremely low/ impaired, TIQ 68
Speech development	normal	delayed	normal	delayed	delayed
Receptive language	normal	below average	normal	below average	below average
Verbal ability	normal	pronunciation problems, grammar problems	normal	pronunciation problems at younger age	pronunciation problems, uses only very short sentences
Comprehension	normal	below average	normal	below average	below average
Neuropsychiatric signs	ASD, attention deficit, anxiety, tics	attention deficit	none	ASD, anxiety, emotional problems	attention deficit
Seizures/epilepsy	no	epilepsy, absences, myoclonus, eyelid myoclonia	epilepsy, generalized tonic-clonic seizures during sleep until 11 years, then seizures remitted, Valproate responsive	no	no
Electro-encephalography	normal	irregular polyspikes and spike-waves, photoparoxysmal reaction	generalized abnormalities at 3 years 8 months, normal at 12 years 6 months	ND	ND

abt, about; ASD, autism spectrum disorder; ND, no data; TIQ, total intelligence quotient.

Boltzmann analysis yielded voltages of -6.45 ± 5.42 mV ($n = 17$) and -57.9 ± 4.30 mV ($n = 18$) for half-maximal activation and inactivation, respectively. Except for a decreased slope factor (steepening) of the p.Arg92Cys activation curve, the voltage dependence of activation remained unaltered for all variants. The voltage dependence of steady-state inactivation was similar to Kv4.1 WT for p.Arg92Cys but negatively shifted for p.Asp115Asn and p.Lys450* with no significant effects on the steepness of inactivation curves (Figure 3B, results summarized in Table S3). To summarize, functional analyses of group 1 variants p.Arg92Cys, p.Asp115Asn, and p.Lys450* in a ternary channel configuration revealed that (1) all three variants significantly differ from WT, and (2) each of them displays distinct variant-specific alterations of biophysical channel properties, thus providing evidence for their pathogenicity. Since the two remaining group 1 variants, p.Tyr61Cysfs*31 and p.Arg99*, which were not functionally studied, are predicted to result in complete LOF, either due to NMD of the respective mRNA or the inability of the encoded protein to form functional channels, the above data collectively suggest that all five identified group 1 *KCND1* variants are pathogenic, impairing Kv4.1 channel function and resulting in an X-linked neurodevelopmental disorder with variable expressivity.

From a clinical perspective, group 1 *KCND1* variants are associated with a neurodevelopmental disorder variable in severity, with or without seizures (Table 2). First clinical signs were noted between the neonatal period and 10 years of age. Four individuals exhibit neuropsychiatric signs, including autism spectrum disorder (ASD), attention deficit disorder, anxiety, tics, and emotional problems. In addition, three of these subjects present with speech delay, receptive language and comprehension problems, and

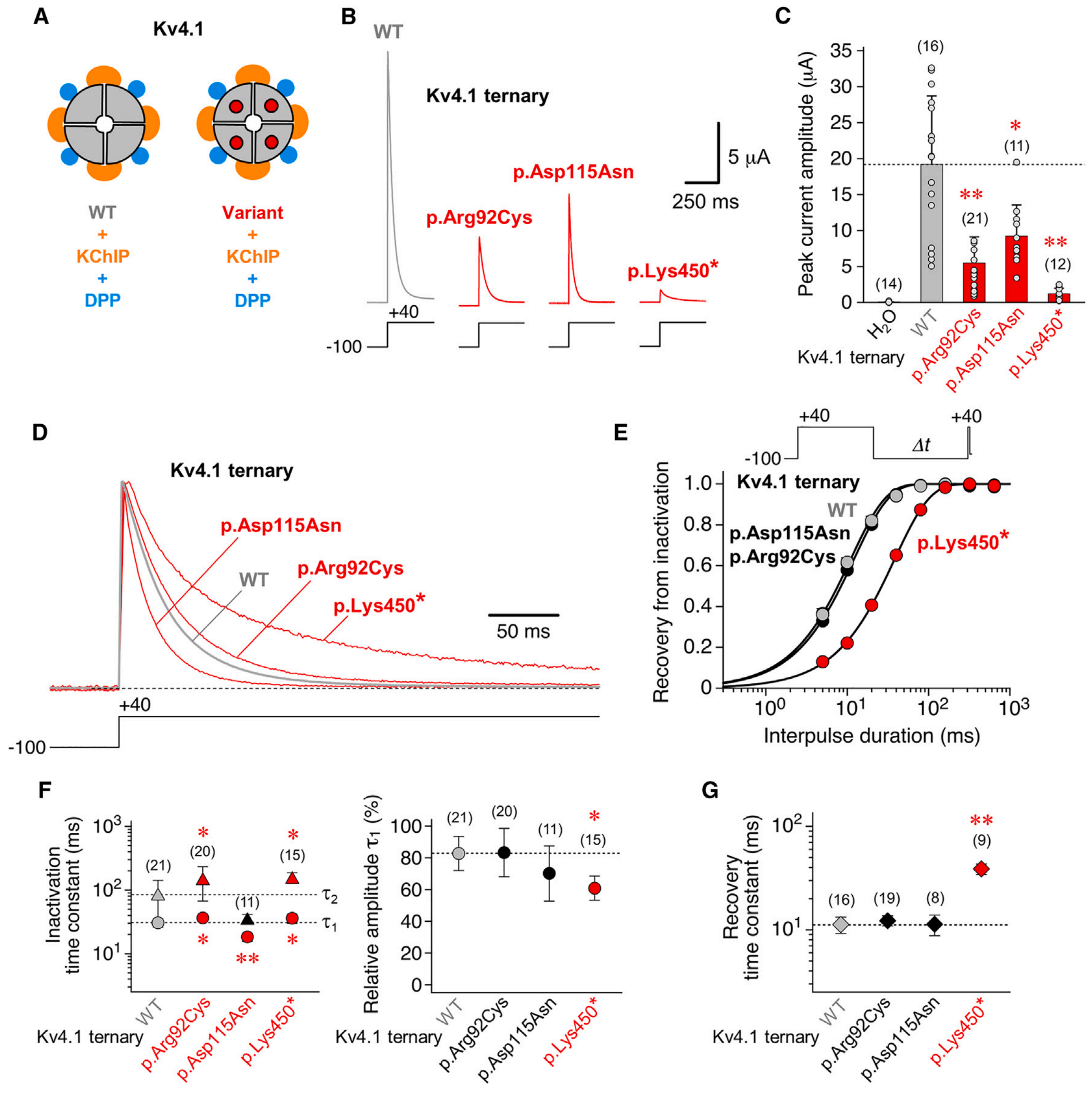

Figure 2. Functional expression and kinetic analysis of inactivation for *KCND1* WT and group 1 variants in a ternary configuration
(A) Kv4.1 wild-type channels (WT, gray quadrants) and variant channels with amino acid substitutions (quadrants with red dots) were functionally analyzed in a ternary configuration, i.e., with both KChIP (orange) and DPP (blue, see also Figure S1 and Video S1) in *Xenopus laevis* oocytes (see material and methods).
(B) Macroscopic currents mediated by Kv4.1 WT, p.Arg92Cys, p.Asp115Asn, and p.Lys450* ternary channels. Currents were recorded under two-electrode voltage clamp using depolarizing voltage steps from −100 to +40 mV, as indicated (gray trace: WT; red traces: p.Arg92Cys, p.Asp115Asn, and p.Lys450*).
(C) Bar graph shows mean peak current amplitudes, including individual data points (number of observations indicated). Note that for all functionally tested group 1 variants (red bars) mean peak current amplitudes were smaller than for Kv4.1 WT (gray bar and horizontal dotted line). Water-injected oocytes served as control.
(D) Normalized currents mediated by Kv4.1 WT (gray trace), p.Arg92Cys, p.Asp115Asn, and p.Lys450* (red traces) in a ternary configuration. Currents were elicited by extended voltage pulses, as indicated (2.5 s, +40 mV), to study the kinetics of macroscopic inactivation (i.e., current decay).
(E) Recovery of ternary channels from inactivation was measured using a double-pulse protocol with long control and brief test pulses to +40 mV and variable interpulse durations at −100 mV (Δt, see inset). The normalized data ($I_{test}/I_{control}$) were plotted against the interpulse duration and described by a single-exponential function (gray symbols: Kv4.1 WT; black symbols: p.Arg92Cys and p.Asp115Asn; red symbols: p.Lys450*; error bars are SEM and smaller than symbols).
(F) Inactivation time constants obtained by fitting the current decay kinetics with a double-exponential function (τ_1: circles, τ_2: triangles) and the relative amplitude of the total decay accounted for by τ_1.
(G) Recovery time constants obtained by fitting the kinetics of recovery from inactivation with a single-exponential function.
(F and G) Gray symbols and horizontal dotted lines: WT; black symbols: values obtained for variant ternary channels with no difference compared to Kv4.1 WT; red symbols: values obtained for variant ternary channels that significantly differ from Kv4.1 WT; number of observations indicated; all statistics based on one way ANOVA with Dunnett's post-hoc testing; significant differences compared to Kv4.1 WT are indicated with $*p < 0.05$ or $**p < 0.0001$.

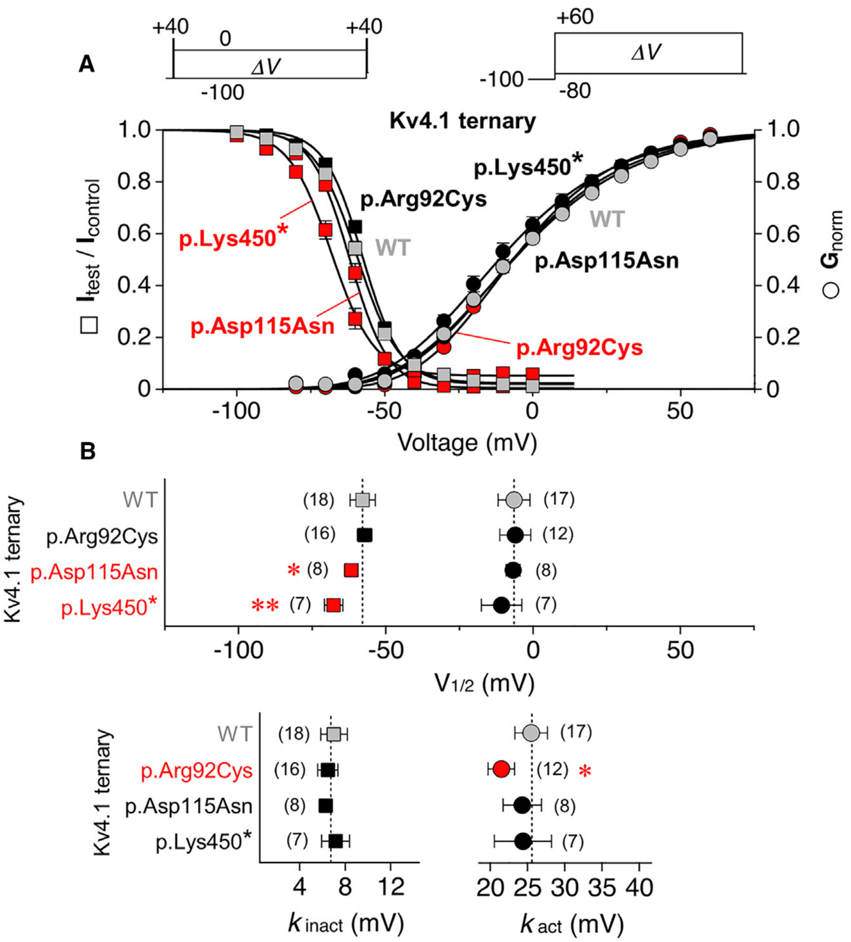

Figure 3. Voltage dependence of activation and steady-state inactivation

(A) Voltage dependences of peak conductance activation (circles) and steady-state inactivation (squares) are shown for Kv4.1 wild-type (WT), p.Arg92Cys, p.Asp115Asn, and p.Lys450* ternary channels. For the study of steady-state inactivation, brief control and test pulses to +40 mV were separated by a 10 s conditioning pulse (ΔV between −100 and 0 mV in 10 mV increments, see inset). Normalized data ($I_{test}/I_{control}$) were plotted against the conditioning pulse voltage. For the study of peak conductance activation, test pulses to voltages between −80 and +60 mV (ΔV in 10 mV increments) were applied from −100 mV (see inset). Normalized conductance values were plotted against the test pulse voltage. The data were fitted with appropriate Boltzmann functions.[11,36] Gray symbols: Kv4.1 WT; black symbols: data which do not differ from Kv4.1 WT; red symbols: data that differ from Kv4.1 WT (error bars are SEM).

(B) Voltages of half-maximal inactivation (squares) and half-maximal activation (circles) and corresponding slope factors (k_{inact} and k_{act}, respectively); gray symbols and vertical dotted lines: Kv4.1 WT; black symbols: $V_{1/2}$ and k values of variant ternary channels with no difference compared to Kv4.1 WT; red symbols: $V_{1/2}$ and k values of variant ternary channels that significantly differ from Kv4.1 WT; number of observations indicated; all statistics based on one way ANOVA with Dunnett's post-hoc testing; significant differences compared to Kv4.1 WT are indicated with *$p < 0.05$ or **$p < 0.0001$.

impaired intellectual development. Two individuals have different types of epilepsy, while two others present with delayed motor development. There is no apparent genotype-phenotype correlation.

Maternally inherited *KCND1* missense variants (group 2)

In addition to the five group 1 variants described above, we identified 13 male individuals carrying 12 distinct maternally inherited *KCND1* missense variants (group 2; Table 1), which were initially considered variants of unknown significance (VUSs). Eleven group 2 variants are private in unrelated males, while p.Thr516Ser was identified in two brothers of different age (individuals 15 and 16; Table 1). The phenotypic spectrum associated with group 2 variants overlaps with the range of phenotypes exhibited by the individuals bearing group 1 variants (Table S1). All 18 individuals present with neurological, albeit rather variable, abnormalities. Frequent clinical signs include impaired intellectual development (14 individuals), delayed speech development (14 individuals), impaired comprehension (13 individuals), neuropsychiatric signs (11 individuals), impaired receptive language (nine individuals), delayed motor development (eight individuals), muscular hypotonia (seven individuals), and different forms of epilepsy (six individuals). Neuropsychiatric features include ASD, attention deficit, hyperactivity, anxiety, tics, emotional problems, and poor social skills. Of note, the two differently aged brothers carrying the c.1546A>T (p.Thr516Ser) variant (individuals 15 and 16) share a common phenotype with only marginal differences. Both boys present with speech delay, reduced receptive language and comprehension, delayed intellectual development, and abnormalities in cerebral magnetic resonance imaging, while until now only the younger brother has been diagnosed with ASD. Thus, despite a rather large clinical variability within the entire cohort, a particular variant may be associated with a more defined phenotypic pattern. It is also worth noting that the variant p.His308Tyr is the one that deviates the most from Kv4.1 WT in terms of its biophysical parameters (see below). However, since the affected child was only two years old at the time of the most recent medical examination, it is not yet possible to definitively state whether these drastic functional changes correlate with a particularly severe phenotype (individual 13). Subject 14 deceased at the age of eight months due to respiratory insufficiency, probably linked to progressive hypomyelinating encephalopathy. Reportedly, in all cases, the heterozygous carrier mothers are clinically unaffected.

Within group 2, the pathogenicity of six variants is strongly supported by the fact that they are not listed as

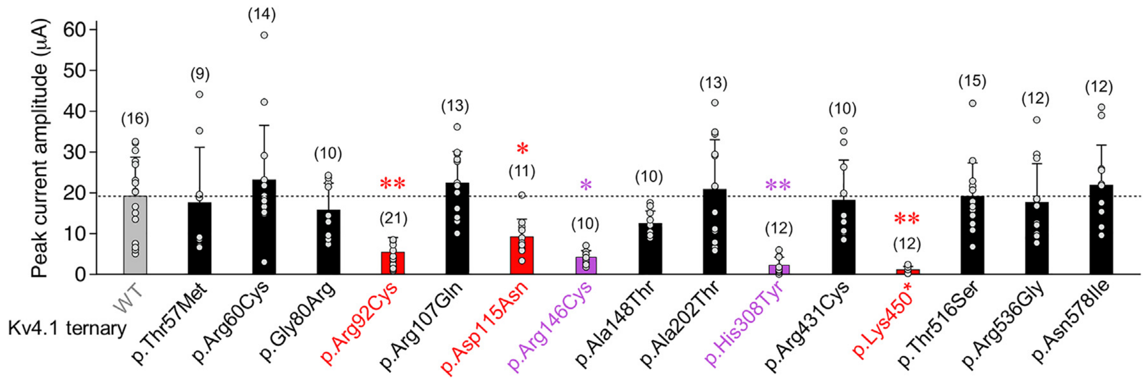

Figure 4. Functional expression of group 2 variants in a ternary configuration
Mean peak current amplitudes at +40 mV including individual data points (number of observations indicated; gray bar and horizontal dotted line: Kv4.1 WT data from Figure 2C; red bars: variant ternary channel data from Figure 2C; black bars: Peak amplitude values for variant ternary channels with no difference compared to Kv4.1 WT; purple bars: peak amplitude values of variant ternary channels that significantly differ from Kv4.1 WT; all statistics based on one way ANOVA with Dunnett's post-hoc testing; significant differences compared to Kv4.1 WT are indicated with *$p < 0.05$ or **$p < 0.0001$.

hemizygous variants in gnomAD (p.Thr57Met, p.Arg60Cys, p.Gly80Arg, p.Ala148Thr, p.His308Tyr, and p.Asn578Ile). The combined data from four distinct pathogenicity prediction algorithms indicate that six variants (p.Thr57Met, p.Arg146Cys, p.His308Tyr, p.Arg431Cys, p.Arg536Gly, and p.Asn578Ile) are likely pathogenic, while the prediction for the other six variants is rather ambiguous.

On the protein level, nine of the 12 substituted amino acid residues are conserved throughout the Kv4 subfamily (Figure 1A), thus suggesting potential biological relevance. Variant-specific amino acid exchanges primarily reside in the cytoplasmic N- or C-termini of Kv4.1, with only two exceptions affecting S1 or S4 (Figures 1 and S1 and Video S1). While these findings appear to suggest that both intracellular termini of Kv4.1 represent hotspots for disease-associated amino acid substitutions, the exact pathogenic potential of the respective variants in a hemizygous state remains unclear. Thus, after having ensured based on immunocytochemical experiments with epitope-tagged constructs that all variants are synthesized and integrated in the plasma membrane of transfected human cells (data not shown), we aimed at their functional characterization to aid clinical variant classification.

When tested in a ternary configuration (i.e., in the presence of both KChIP and DPP), all 12 maternally inherited missense variants mediated A-type currents and thus allowed further functional characterization. Of note, all group 2 variants as well as the three previously studied group 1 variants were included in the statistical analysis of the functional data. Reduced peak current amplitudes compared to Kv4.1 WT were only seen for p.Arg146Cys and p.His308Tyr (Figure 4). However, macroscopic inactivation kinetics were differentially altered for six out of the 12 variants, with an overall acceleration for p.Arg107Gln and p.Arg146Cys and an overall slowing for p.His308Tyr, p.Thr516Ser, and p.Asn578Ile, while in p.Ala148Thr only the relative amplitude of τ_1 was reduced in comparison to Kv4.1 WT (Figure 5A). Notably, in none of the 12 variants, the recovery from inactivation was affected, except for p.His308Tyr, which showed a remarkable about 100-fold slowing (Figure 5B). The voltage dependences of activation and/or steady-state inactivation were modified for three of the 12 variants. Thus, for p.His308Tyr, both voltage dependences were negatively shifted, whereas for p.Ala202Thr and p.Thr516Ser, both voltage dependences were positively shifted. In addition, increased slope factors (flattening) were obtained for the p.Ala202Thr inactivation curve and the p.His308Tyr activation curve (Figure 5C, results summarized in Table S3). Taken together, when tested in a ternary configuration, significant variant effects on one or more electrophysiological parameters were observed for seven out of 12 group 2 variants. It should be noted that, due to the inclusion of a large number of variants in addition to the initially studied group 1 variants, and particularly due to the drastic effects caused by p.His308Tyr, ANOVA might underestimate the significance of effects associated with some of the variants.

Variant-associated effects in Kv4.1 homotetramers and binary channel complexes

In the absence of a precise knowledge of the availability of KChIP and DPP subunits at early developmental stages, in specific brain regions, cell types, or subcellular compartments, further electrophysiological experiments were performed with Kv4.1 homotetramers and binary channel complexes (containing only one of the two auxiliary β subunits). First, Kv4.1 WT and variant channels were examined in the absence of auxiliary β subunits to find out whether a variant exerts an intrinsic effect on biophysical properties. Second, channels were synthesized together with either KChIP or DPP to assess possible dysfunctions of binary channel complexes (results summarized in Tables S4–S6). Our experiments revealed for instance that, although inconspicuous in the ternary configuration, p.Gly80Arg, p.Ala202Thr, p.Thr516Ser, and p.Asn578Ile show an increase, whereas p.Arg431Cys shows a decrease in peak current amplitude in the absence of auxiliary β

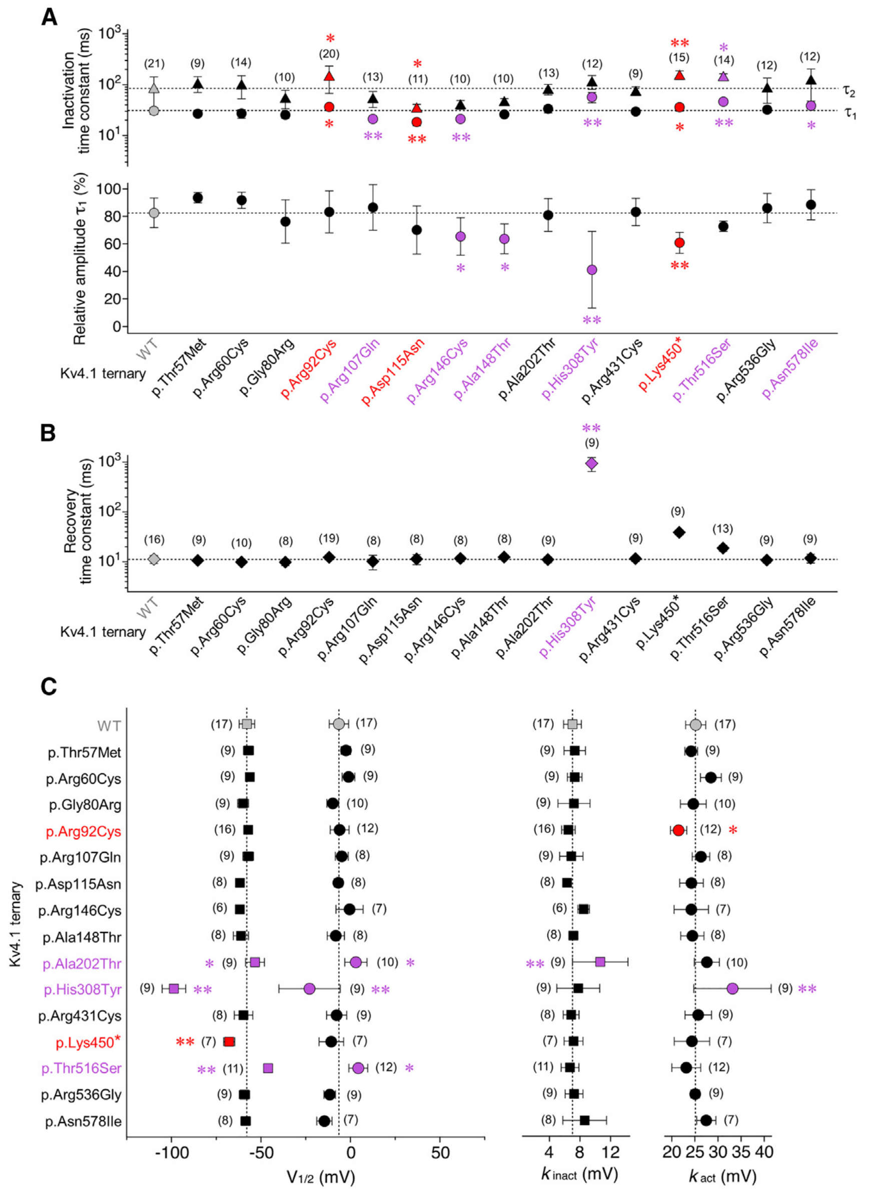

Figure 5. Biophysical characterization of group 2 variants in a ternary configuration
(A) Inactivation time constants obtained by fitting the Kv4.1-mediated current decay kinetics with a double-exponential function (τ_1: circles, τ_2: triangles) and the relative amplitude of the total decay accounted for by τ_1 (number of observations indicated).
(B) Recovery time constants obtained by fitting the kinetics of recovery from inactivation with a single-exponential function (number of observations indicated).
(C) Voltages of half-maximal inactivation (squares) and half-maximal activation (circles) and corresponding slope factors (k_{inact} and k_{act}, respectively; number of observations indicated).
(A–C) Gray symbols and dotted lines: Kv4.1 WT data from Figures 2F, 2G, and 3B; red symbols: variant ternary channel data from Figures 2F, 2G, and 3B that significantly differ from Kv4.1 WT when testing 15 variants, including p.His308Tyr; black symbols: values obtained for variant ternary channels with no difference compared to Kv4.1 WT (including part of the p.Asp115Asn and p.Lys450* data); purple symbols: values obtained for variant ternary channels (maternally inherited missense variants) that significantly differ from Kv4.1 WT; number of observations indicated; all statistics based on one way ANOVA with Dunnett's post-hoc testing; significant differences compared to Kv4.1 WT are indicated with $*p < 0.05$ or $**p < 0.0001$.

subunits. Similarly, macroscopic inactivation kinetics were accelerated for p.Thr57Met and slowed for p.Gly80Arg and p.Arg536Gly, and concerning the voltage dependences, positive shifts were seen for p.Thr57Met and p.Arg92Cys, and negative shifts were seen for p.Asp115Asn, p.Ala148Thr, p.Arg431Cys, and p.Asn578Ile in the absence of auxiliary β subunits but not in the ternary configuration. For other variants, intrinsic functional alterations correspond to those observed in the ternary configuration. Thus, peak current amplitudes were intrinsically lowered for p.Arg92Cys, p.His308Tyr, and p.Lys450*; macroscopic inactivation kinetics were intrinsically slowed for p.Lys450* and p.Asn578Ile; and recovery kinetics were intrinsically slowed for p.His308Tyr. Finally, the voltage dependence of steady-state inactivation was intrinsically shifted positive for p.Ala202Thr and p.Thr516Ser, and both voltage dependences were intrinsically shifted negative for p.His308Tyr. Taken together, our findings show that variant-specific alterations of individual biophysical parameters are often apparent in all four tested channel configurations. On the other hand, the only functional alteration found for p.Arg60Cys was a modest acceleration of macroscopic inactivation kinetics, reflected by an increase in the relative amplitude of τ_1 in the DPP binary complex.

Of note, the presence of only one β subunit (binary channels) also yielded valuable information on whether a variant channel was still able to functionally interact with KChIP or DPP. As seen for Kv4.1 WT and the majority of variants, the concurrent presence of KChIP slowed the initial phase and accelerated the late phase of macroscopic inactivation, accelerated the recovery from inactivation, and positively shifted the voltage dependence of steady-state inactivation, whereas the presence of DPP accelerated both the initial phase of macroscopic inactivation and recovery from inactivation and negatively shifted the voltage dependences of both activation and steady-state inactivation. These typical β-subunit effects on Kv4.1 channel function were lost for certain variants (see Figures S3–S5) thus representing a further putative pathophysiological mechanism.

Classification of *KCND1* group 2 variants

Finally, an assessment of the pathogenicity probability of group 2 variants should be made, taking into account the above findings. Thus, we relied on the well-established American College of Medical Genetics and Genomics (ACMG) guidelines for clinical variant interpretation.[39,40] This classification system utilizes the evidence code PS3 based on the results of "well-established" functional assays, which are suitable to discriminate between abnormal and normal gene/protein function. In the past, the highly reproducible and robust two-electrode voltage-clamp technique in *Xenopus* oocytes has been used successfully to assess the biophysical features of ion channels, including variants of *KCND2*/Kv4.2[8,9,10,41,42] and *KCND3*/Kv4.3,[43–45] as well as other voltage-gated potassium channels.[46] Therefore, we performed PS3 scoring of *KCND1* variants on the basis of the results from this functional assay system. In particular, to rate functional impairment of Kv4.1 variants, we included the five biophysical parameters examined (peak current amplitude, macroscopic inactivation, recovery from inactivation, and the voltage dependences of activation and steady-state inactivation) from all analyzed channel configurations (ternary complexes, Kv4.1 alone, and both binary channel compositions). In addition, we incorporated the functional interaction of Kv4.1 variants with KChIP2 and/or DPP6. Thus, to assess physiological impairment of a given functionally analyzed variant, 29 items were taken into account (Table S7). If four or more of these functional parameters are significantly altered compared to Kv4.1 WT, this is valued as supportive evidence for a pathogenic effect on channel function (ACMG criterion PS3, see discussion). This definition is based on the idea that variants showing normal interaction with auxiliary β subunits meet the requirements to pass the threshold if they differ from Kv4.1 WT in only one of the five biophysical parameters examined but do so consistently in all four channel configurations tested. All but two group 2 variants (p.Arg60Cys and p.Arg536Gly) exceed this threshold value (Table S7), with p.His308Tyr reaching the maximum number of 22 altered parameters, some of which are drastically affected. For comparison, 12 (p.Asp115Asn) and 15 (p.Arg92Cys and p.Lys450*) items are significantly altered in the functionally analyzed pathogenic group 1 variants. According to ACMG classification, three group 2 variants are considered to be likely pathogenic, whereas the nine remaining variants are categorized as VUSs (Table 3).

Discussion

Here, we describe 17 distinct hemizygous coding variants of *KCND1*, which were independently identified as the most-likely disease-causing variant in 18 male subjects presenting with a variable neurodevelopmental phenotype. To determine variant pathogenicity, we employed genomic data analyses and comprehensive functional characterization of biophysical channel features. Our results provide strong supporting evidence that hemizygous *KCND1* variants that differentially alter Kv4.1 channel properties are involved in the pathogenesis of an X-linked neurodevelopmental condition, which is often associated with a variable neuropsychiatric phenotype.

Clinical phenotypes

The *KCND1* variants described herein are associated with a neurodevelopmental disorder characterized by a remarkable variability in the phenotypic expression and severity of the disease. Although the clinically documented onset of the disease varies from neonatal to ten years of age, all but two individuals were younger than five years of age when the first clinical signs were diagnosed. In the two exceptions, it is not known whether an earlier clinical diagnosis would have been possible. The disease phenotype of the entire cohort ranges from rather complex and severe neurological phenotypes to more or less isolated clinical signs. Individual clinical features noticed in isolation were also observed as syndromic attributes. Prevalent clinical signs are intellectual disability, delayed speech development, hindered comprehension, neuropsychiatric signs, impaired receptive language, motor delay, muscular hypotonia, and different types of epilepsy. Neuropsychiatric abnormalities observed in about two-thirds of the subjects include ASD, attention deficit, hyperactivity, anxiety, tics, emotional problems, and poor social skills. There is no obvious genotype-phenotype correlation, e.g., similar genetic alterations or amino acid exchanges affecting identical parts of the channel protein do not appear to result in a common phenotype. Several *KCND1* variants, including two predicted LOF variants, are listed as rare hemizygous variants in gnomAD v4.0.0 (Table 1). This finding is consistent with a highly variable disease phenotype that includes adult males with a mild (or late-onset) or even no apparent clinical phenotype, thus potentially broadening the clinical variability described herein. Unfortunately, to our knowledge, there are no suitable published or otherwise available data on a *KCND1* LOF animal model to verify this hypothesis. However, in a single individual with childhood-onset focal epilepsy, a hemizygous *KCND1* nonsense variant was identified as the prime candidate for a pathogenic variant.[29] Moreover, LOF hemizygotes are listed in gnomAD for several well-known X-linked neurodevelopmental genes, including genes that also encode transmembrane proteins with ion channel/transporter function, such as *GRIA3*[47] (MIM: 305915) and *SLC9A6*[48] (MIM: 300231) for both of which pathogenic LOF variants have been described. It should also be noted that the *KCND1* cohort presented in our study is likely to be biased to the more severe end of the phenotypic spectrum, as all subjects were selected based on distinctive clinical abnormalities exhibited during childhood. Taken together, it is tempting to speculate that hemizygous *KCND1* variants lead to a wide-ranging continuum of neurodevelopmental abnormalities reaching from clinically largely unremarkable

Table 3. Classification of *KCND1* group 2 variants

Individual	Transcript GenBank: NM_004979.6	protein GenBank: NP_004970.3	Used ACMG criteria	ACMG classification[a]
**Maternally inherited missense variants**				
**Variants affecting the N-terminal cytoplasmic domain**				
6	c.170C>T	p.Thr57Met	PS3, PM2_sup, PP3	likely pathogenic
7	c.178C>T	p.Arg60Cys	PM2_sup, PP3	VUS
8	c.238G>C	p.Gly80Arg	PS3, PM2_sup, BP4	VUS
9	c.320G>A	p.Arg107Gln	PS3	VUS
10	c.436C>T	p.Arg146Cys	PS3, PP3	VUS
11	c.442G>A	p.Ala148Thr	PS3, PM2_sup	VUS
**Variants affecting transmembrane domains**				
12	c.604G>A	p.Ala202Thr	PS3, BS2	VUS
13	c.922C>T	p.His308Tyr	PS3, PM2_supp, PP3	likely pathogenic
**Variants affecting the C-terminal cytoplasmic domain**				
14	c.1291C>T	p.Arg431Cys	PS3, PP3	VUS
15 and 16	c.1546A>T	p.Thr516Ser	PS3, BP4, BS2	VUS
17	c.1606A>G	p.Arg536Gly	PP3	VUS
18	c.1733A>T	p.Asn578Ile	PS3, PM2_sup, PP3	likely pathogenic

American College of Medical Genetics and Genomics (ACMG) criteria: PS3, well-established *in vitro* or *in vivo* functional studies supportive of a damaging effect on the gene or gene product; PM2, absent from controls (or at extremely low frequency if recessive) in Exome Sequencing Project, 1000 Genomes Project, or Exome Aggregation Consortium; PP3, multiple lines of computational evidence support a deleterious effect on the gene or gene product (conservation, evolutionary, splicing impact, etc.); BP4, multiple lines of computational evidence suggest no impact on gene or gene product (conservation, evolutionary, splicing impact, etc.); BS2, variant was observed in a hemizygous state in population databases more than expected for disease.
[a]To assign variant class, a points-based system to simplify scoring was used.[40] See Table S7 for more details on PS3 scoring.

phenotypes to early-onset, rather severe syndromic conditions. While the reasons for this large degree of phenotypic variability are currently unknown, it is important to note that distinct variants of a given gene are unlikely to act in isolation. Instead, other genomic variants may act as genetic modifiers to either alleviate or exacerbate disease severity, thus leading to a variable phenotypic outcome. In the future, it will be interesting to identify genetic modifiers of pathogenic *KCND1* variants and determine their mutual interaction. Attractive candidate modifiers are, e.g., genes encoding proteins that interact with Kv4.1, such as members of the KChIP and DPP families.

ACMG classification

Our genetic and functional data provide strong evidence that all five *KCND1* group 1 variants are pathogenic. Based on these findings, the evidence of pathogenicity for all group 2 variants was formally evaluated in accordance with ACMG standards and guidelines (Table 3).[39,40] For functionally tested variants the criterion PS3 (well-established *in vitro* or *in vivo* functional studies supportive of a damaging effect on the gene or gene product) was rated utilizing a functional assessment table based on significance levels with no reference to the direction and amount of change (i.e., gain or LOF; Table S7). In order to meet the PS3 criterion, at least four electrophysiological items need to be significantly altered, accounting for the possibility that a variant may differ from Kv4.1 WT in only one electrophysiological parameter, but does so in all four tested channel configurations. To keep the scoring system simple, quantitative differences in significant variant effects are not further differentiated. Likewise, all analyzed parameters are assumed to be of equivalent pathogenic significance and are therefore given equal weight. Overall, ACMG classification categorizes three group 2 variants (p.Thr57Met, p.His308Tyr, and p.Asn578Ile) as likely pathogenic, thus providing supporting evidence that these variants are involved in the pathogenesis of a neurodevelopmental disorder. The remaining group 2 missense variants are classified as VUS. Further studies are needed to determine their involvement in pathogenicity.

Molecular pathophysiology of *KCND1* variants

A closer inspection of the structure-function relationships specifically affected by individual variants may help to understand the molecular pathophysiology of the disorder described herein. Strikingly, the vast majority of *KCND1* missense variants described herein leads to widely scattered amino acid exchanges within the cytoplasmic N- and C-termini at sites that are presumably not quite essential for Kv4.1 channel function (Figures 1 and S1). Although most amino acid exchanges lead to alterations in the number (mostly a reduction) and nature of polar side chain interactions (Figure S2; Table S2), the resultant biophysical alterations are mostly moderate. In contrast, published heterozygous missense variants in *KCND2* associated with a

neurodevelopmental disease phenotype alter sites within the S4-S5 linker and the distal S6 segment, core regions of the protein known to play a pivotal role in the operation of the cytoplasmic gate, with a prominent impact on Kv4.2 channel gating.[8,9,10,11] This obvious difference may reflect natural selection due to the gonosomal and autosomal localization of *KCND1* and *KCND2*, respectively.[34] Putative *KCND1* missense variants affecting the S4-S5 linker or the distal S6 segment may be selected against because homomeric variant channels may lead to embryonic lethality of males harboring these variants. Of note, all *KCND1* missense-variant-conducting mothers are reportedly clinically asymptomatic. It will be interesting to see if *KCND1* missense variants affecting the cytoplasmic gate are identified in the future and shown to be associated with a female-specific genetic disease.

In order to define loss- or gain-of-function features for a given variant, the direction of changes in biophysical parameters may be considered. Thus, besides an increase in current amplitude, a slowing of macroscopic inactivation, accelerated recovery kinetics, a positively shifted voltage dependence of steady-state inactivation and/or negatively shifted voltage dependence of activation represent gain-of-function features, and vice versa for LOF. Except for the recovery kinetics, which, if modified, were always slowed; biophysical Kv4.1 alterations are observed in either direction. Thus, observed alterations of biophysical parameters are likely to eventually reflect both LOF and gain-of-function. Overall, variant-specific alterations in electrophysiological parameters or a lack of their modulation by auxiliary β subunits do not appear to correlate with the associated clinical phenotype, neither with individual signs, the sum of phenotypic features, nor the disease severity. Notably, this finding holds true regardless of whether the quantitative and qualitative nature of biophysical changes was taken into account or not (data not shown). Therefore, and for the sake of simplicity, we based the PS3 scoring for ACMG classification of *KCND1* variants exclusively on the sum of significantly altered electrophysiological parameters (Tables S3–S7), thus not accounting for the qualitative and quantitative nature of variant effects.

Cellular mechanism of this neurodevelopmental disorder

In order to determine cellular mechanisms that may be critically influenced by Kv4.1 channel dysfunction or complete LOF, it is important to consider the brain regions in which *KCND1* is expressed and the physiological role of Kv4.1 channels in specific neurons. While *KCND1* expression seems to be relatively moderate compared to other ion channel genes,[34,49] it is widely expressed throughout the human brain and has been shown to be involved in the control of slow repetitive action potential firing in many different neurons.[50–53] This is reminiscent of the A-type current function originally identified in primitive animals,[54] where these channels appear to already activate at very negative membrane potentials well below threshold. Thereby, they cause a delay in firing until A-type channel inactivation allows for the next spike. It is obvious that the amount and distribution of A-type channels on the cell surface as well as their biophysical properties, especially their macroscopic inactivation kinetics, are critical for this function. However, none of the reported Kv4.1-associated functional roles, including hippocampal pattern separation,[52] clock gene expression,[51] control of striatal circuitry,[53] or nociception,[50] appear to directly relate to the neurodevelopmental disease phenotype described herein. Intriguingly, Kv4.1 has also been implicated in the control of cell proliferation, not only in tumor cells,[55,56] but also in neural stem and progenitor cells.[57] Thus, at certain developmental stages, even minor disturbances in Kv4.1 channel levels and/or biophysical properties, representing either gain of function or LOF, may significantly affect neuronal cell proliferation and migration, thus impairing neuronal development. In addition, clock gene expression reportedly under the control of Kv4.1 channel activity[51] is known to regulate cortical development,[58] thus representing a further possible explanation for the high phenotypic variability of the described disorder to be explored in the future.

Data and code availability

Additional methodological, genetic, and clinical data supporting the findings of this study are available from the corresponding authors upon reasonable request. In some cases, specific privacy regulations and the consent under which specific data were obtained may not permit the sharing of additional findings, e.g., raw next-generation sequencing files.

Supplemental information

Supplemental information can be found online at https://doi.org/10.1016/j.ajhg.2024.04.019.

Acknowledgments

We thank the family members for their participation and collaboration. This study makes use of data generated by the DECIPHER community.[59] A full list of centers that contributed to the generation of the data is available from http://decipher.sanger.ac.uk and via email from decipher@sanger.ac.uk. This work was supported by the Deutsche Forschungsgemeinschaft (BA2055/1-3 and BA2055/6-1 to R.B. and SCHO 1921/2-1 to C.S.), the Netherlands Organisation for Scientific Research (ZonMw Vidi, grant 09150172110002 to T.S.B.), an Erasmus MC Fellowship 2017 (to T.S.B.), the Erasmus MC Human Disease Model Award 2018 (to T.S.B.), PNRR-MUR-M4C2 PE0000006 Research Program "MNESYS" - A multiscale integrated approach to the study of the nervous system in health and disease (to P.S.), the Italian Ministry of Health (CCR-2017-23669081 and RF-2021-12374963 to M.T.). IRCCS "G. Gaslini" is a member of ERN-Epicare. Funding bodies did not have any influence on study design, results and data interpretation, or final manuscript.

Declaration of interests

The authors declare no competing interests.

Received: November 9, 2023
Accepted: April 25, 2024
Published: May 20, 2024

Web resources

CADD, https://wintervar.wglab.org/
GeneMatcher, https://genematcher.org/
gnomAD database, https://gnomad.broadinstitute.org/
MetaDome, https://stuart.radboudumc.nl/metadome/
PolyPhen-2, http://genetics.bwh.harvard.edu/pph2/
REVEL, https://sites.google.com/site/revelgenomics/
SIFT, https://sift.bii.a-star.edu.sg/

References

1. Gutman, G.A., Chandy, K.G., Grissmer, S., Lazdunski, M., McKinnon, D., Pardo, L.A., Robertson, G.A., Rudy, B., Sanguinetti, M.C., Stühmer, W., and Wang, X. (2005). International Union of Pharmacology. LIII. Nomenclature and molecular relationships of voltage-gated potassium channels. Pharmacol. Rev. *57*, 473–508. https://doi.org/10.1124/pr.57.4.10.
2. Jerng, H.H., Pfaffinger, P.J., and Covarrubias, M. (2004). Molecular physiology and modulation of somatodendritic A-type potassium channels. Mol. Cell. Neurosci. *27*, 343–369. https://doi.org/10.1016/j.mcn.2004.06.011.
3. Kim, J., Wei, D.S., and Hoffman, D.A. (2005). Kv4 potassium channel subunits control action potential repolarization and frequency-dependent broadening in rat hippocampal CA1 pyramidal neurones. J. Physiol. *569*, 41–57. https://doi.org/10.1113/jphysiol.2005.095042.
4. Ramakers, G.M.J., and Storm, J.F. (2002). A postsynaptic transient K^+ current modulated by arachidonic acid regulates synaptic integration and threshold for LTP induction in hippocampal pyramidal cells *99*, 10144–10149. https://doi.org/10.1073/pnas.152620399.
5. Hille, B. (2001). Ion Channels of Excitable Membranes, 3 Edition (Sinauer Associates, Inc.).
6. Bixby, K.A., Nanao, M.H., Shen, N.V., Kreusch, A., Bellamy, H., Pfaffinger, P.J., and Choe, S. (1999). Zn^{2+}-binding and molecular determinants of tetramerization in voltage- gated K^+ channels. Nat. Struct. Biol. *6*, 38–43. https://doi.org/10.1038/4911.
7. Swartz, K.J. (2004). Towards a structural view of gating in potassium channels. Nat. Rev. Neurosci. *5*, 905–916. https://doi.org/10.1038/nrn1559.
8. Bavan, S., Goodkin, H.P., and Papazian, D.M. (2022). Altered closed state inactivation gating in Kv4.2 channels results in developmental and epileptic encephalopathies in human patients. Hum. Mutat. *43*, 1286–1298. https://doi.org/10.1002/humu.24396.
9. Lee, H., Lin, M.c.A., Kornblum, H.I., Papazian, D.M., and Nelson, S.F. (2014). Exome sequencing identifies de novo gain of function missense mutation in *KCND2* in identical twins with autism and seizures that slows potassium channel inactivation. Hum. Mol. Genet. *23*, 3481–3489. https://doi.org/10.1093/hmg/ddu056.
10. Zhang, Y., Tachtsidis, G., Schob, C., Koko, M., Hedrich, U.B.S., Lerche, H., Lemke, J.R., van Haeringen, A., Ruivenkamp, C., Prescott, T., et al. (2021). *KCND2* variants associated with global developmental delay differentially impair Kv4.2 channel gating. Hum. Mol. Genet. *30*, 2300–2314. https://doi.org/10.1093/hmg/ddab192.
11. Barghaan, J., and Bähring, R. (2009). Dynamic coupling of voltage sensor and gate involved in closed-state inactivation of Kv4.2 channels. J. Gen. Physiol. *133*, 205–224. https://doi.org/10.1085/jgp.200810073.
12. Rivera, J.F., Ahmad, S., Quick, M.W., Liman, E.R., and Arnold, D.B. (2003). An evolutionarily conserved dileucine motif in Shal K^+ channels mediates dendritic targeting. Nat. Neurosci. *6*, 243–250. https://doi.org/10.1038/nn1020.
13. An, W.F., Bowlby, M.R., Betty, M., Cao, J., Ling, H.P., Mendoza, G., Hinson, J.W., Mattsson, K.I., Strassle, B.W., Trimmer, J.S., and Rhodes, K.J. (2000). Modulation of A-type potassium channels by a family of calcium sensors. Nature *403*, 553–556. https://doi.org/10.1038/35000592.
14. Nadal, M.S., Ozaita, A., Amarillo, Y., Vega-Saenz de Miera, E., Ma, Y., Mo, W., Goldberg, E.M., Misumi, Y., Ikehara, Y., Neubert, T.A., and Rudy, B. (2003). The CD26-related dipeptidyl aminopeptidase-like protein DPPX is a critical component of neuronal A-Type K^+ channels. Neuron *37*, 449–461. https://doi.org/10.1016/s0896-6273(02)01185-6.
15. Bähring, R., Dannenberg, J., Peters, H.C., Leicher, T., Pongs, O., and Isbrandt, D. (2001). Conserved Kv4 N-terminal domain critical for effects of Kv channel-interacting protein 2.2 on channel expression and gating. J. Biol. Chem. *276*, 23888–23894. https://doi.org/10.1074/jbc.M101320200.
16. Beck, E.J., Bowlby, M., An, W.F., Rhodes, K.J., and Covarrubias, M. (2002). Remodelling inactivation gating of Kv4 channels by KChIP1, a small-molecular-weight calcium-binding protein. J. Physiol. *538*, 691–706. https://doi.org/10.1113/jphysiol.2001.013127.
17. Jerng, H.H., Qian, Y., and Pfaffinger, P.J. (2004). Modulation of Kv4.2 channel expression and gating by dipeptidyl peptidase 10 (DPP10). Biophys. J. *87*, 2380–2396. https://doi.org/10.1529/biophysj.104.042358.
18. Shibata, R., Misonou, H., Campomanes, C.R., Anderson, A.E., Schrader, L.A., Doliveira, L.C., Carroll, K.I., Sweatt, J.D., Rhodes, K.J., and Trimmer, J.S. (2003). A fundamental role for KChIPs in determining the molecular properties and trafficking of Kv4.2 potassium channels. J. Biol. Chem. *278*, 36445–36454. https://doi.org/10.1074/jbc.M306142200.
19. Zagha, E., Ozaita, A., Chang, S.Y., Nadal, M.S., Lin, U., Saganich, M.J., McCormack, T., Akinsanya, K.O., Qi, S.Y., and Rudy, B. (2005). DPP10 modulates Kv4-mediated A-type potassium channels. J. Biol. Chem. *280*, 18853–18861. https://doi.org/10.1074/jbc.M410613200.
20. Callsen, B., Isbrandt, D., Sauter, K., Hartmann, L.S., Pongs, O., and Bähring, R. (2005). Contribution of N- and C-terminal Kv4.2 channel domains to KChIP interaction. J. Physiol. *568*, 397–412. https://doi.org/10.1113/jphysiol.2005.094359.
21. Kise, Y., Kasuya, G., Okamoto, H.H., Yamanouchi, D., Kobayashi, K., Kusakizako, T., Nishizawa, T., Nakajo, K., and Nureki, O. (2021). Structural basis of gating modulation of Kv4 channel complexes. Nature *599*, 158–164. https://doi.org/10.1038/s41586-021-03935-z.
22. Ren, X., Hayashi, Y., Yoshimura, N., and Takimoto, K. (2005). Transmembrane interaction mediates complex formation

between peptidase homologues and Kv4 channels. Mol. Cell. Neurosci. *29*, 320–332. https://doi.org/10.1016/j.mcn.2005.02.003.

23. Ma, D., Zhao, C., Wang, X., Li, X., Zha, Y., Zhang, Y., Fu, G., Liang, P., Guo, J., and Lai, D. (2022). Structural basis for the gating modulation of Kv4.3 by auxiliary subunits. Cell Res. *32*, 411–414. https://doi.org/10.1038/s41422-021-00608-4.
24. Maffie, J., and Rudy, B. (2008). Weighing the evidence for a ternary protein complex mediating A-type K^+ currents in neurons. J. Physiol. *586*, 5609–5623. https://doi.org/10.1113/jphysiol.2008.161620.
25. Barghaan, J., Tozakidou, M., Ehmke, H., and Bähring, R. (2008). Role of N-terminal domain and accessory subunits in controlling deactivation-inactivation coupling of Kv4.2 channels. Biophys. J. *94*, 1276–1294. https://doi.org/10.1529/biophysj.107.111344.
26. Allen, N.M., Weckhuysen, S., Gorman, K., King, M.D., and Lerche, H. (2020). Genetic potassium channel-associated epilepsies: Clinical review of the Kv family. Eur. J. Paediatr. Neurol. *24*, 105–116. https://doi.org/10.1016/j.ejpn.2019.12.002.
27. Mithyantha, R., Kneen, R., McCann, E., and Gladstone, M. (2017). Current evidence-based recommendations on investigating children with global developmental delay. Arch. Dis. Child. *102*, 1071–1076. https://doi.org/10.1136/archdischild-2016-311271.
28. Shevell, M. (2008). Global developmental delay and mental retardation or intellectual disability: conceptualization, evaluation, and etiology. Pediatr. Clin. *55*, 1071–1084. xi. https://doi.org/10.1016/j.pcl.2008.07.010.
29. Miao, P., Feng, J., Guo, Y., Wang, J., Xu, X., Wang, Y., Li, Y., Gao, L., Zheng, C., and Cheng, H. (2018). Genotype and phenotype analysis using an epilepsy-associated gene panel in Chinese pediatric epilepsy patients. Clin. Genet. *94*, 512–520. https://doi.org/10.1111/cge.13441.
30. Sobreira, N., Schiettecatte, F., Valle, D., and Hamosh, A. (2015). GeneMatcher: a matching tool for connecting investigators with an interest in the same gene. Hum. Mutat. *36*, 928–930. https://doi.org/10.1002/humu.22844.
31. Wiel, L., Baakman, C., Gilissen, D., Veltman, J.A., Vriend, G., and Gilissen, C. (2019). MetaDome: Pathogenicity analysis of genetic variants through aggregation of homologous human protein domains. Hum. Mutat. *40*, 1030–1038. https://doi.org/10.1002/humu.23798.
32. Kircher, M., Witten, D.M., Jain, P., O'Roak, B.J., Cooper, G.M., and Shendure, J. (2014). A general framework for estimating the relative pathogenicity of human genetic variants. Nat. Genet. *46*, 310–315. https://doi.org/10.1038/ng.2892.
33. Adzhubei, I.A., Schmidt, S., Peshkin, L., Ramensky, V.E., Gerasimova, A., Bork, P., Kondrashov, A.S., and Sunyaev, S.R. (2010). A method and server for predicting damaging missense mutations. Nat. Methods *7*, 248–249. https://doi.org/10.1038/nmeth0410-248.
34. Isbrandt, D., Leicher, T., Waldschütz, R., Zhu, X., Luhmann, U., Michel, U., Sauter, K., and Pongs, O. (2000). Gene structures and expression profiles of three human *KCND* (Kv4) potassium channels mediating A-type currents I_{TO} and I_{SA}. Genomics *64*, 144–154. https://doi.org/10.1006/geno.2000.6117.
35. Pongs, O., and Schwarz, J.R. (2010). Ancillary subunits associated with voltage-dependent K^+ channels. Physiol. Rev. *90*, 755–796. https://doi.org/10.1152/physrev.00020.2009.
36. Wollberg, J., and Bähring, R. (2016). Intra- and intersubunit dynamic binding in Kv4.2 channel closed-state inactivation. Biophys. J. *110*, 157–175. https://doi.org/10.1016/j.bpj.2015.10.046.
37. Collins, R.L., Brand, H., Karczewski, K.J., Zhao, X., Alföldi, J., Francioli, L.C., Khera, A.V., Lowther, C., Gauthier, L.D., Wang, H., et al. (2020). A structural variation reference for medical and population genetics. Nature *581*, 444–451. https://doi.org/10.1038/s41586-020-2287-8.
38. Dyle, M.C., Kolakada, D., Cortazar, M.A., and Jagannathan, S. (2020). How to get away with nonsense: Mechanisms and consequences of escape from nonsense-mediated RNA decay. Wiley Interdiscip. Rev. RNA *11*, e1560. https://doi.org/10.1002/wrna.1560.
39. Richards, S., Aziz, N., Bale, S., Bick, D., Das, S., Gastier-Foster, J., Grody, W.W., Hegde, M., Lyon, E., Spector, E., et al. (2015). Standards and guidelines for the interpretation of sequence variants: a joint consensus recommendation of the American College of Medical Genetics and Genomics and the Association for Molecular Pathology. Genet. Med. *17*, 405–424. https://doi.org/10.1038/gim.2015.30.
40. Tavtigian, S.V., Harrison, S.M., Boucher, K.M., and Biesecker, L.G. (2020). Fitting a naturally scaled point system to the ACMG/AMP variant classification guidelines. Hum. Mutat. *41*, 1734–1737. https://doi.org/10.1002/humu.24088.
41. Drabkin, M., Zilberberg, N., Menahem, S., Mulla, W., Halperin, D., Yogev, Y., Wormser, O., Perez, Y., Kadir, R., Etzion, Y., et al. (2018). Nocturnal Atrial Fibrillation Caused by Mutation in KCND2, Encoding Pore-Forming (alpha) Subunit of the Cardiac Kv4.2 Potassium Channel. Circ. Genom. Precis. Med. *11*, e002293. https://doi.org/10.1161/CIRCGEN.118.002293.
42. Lin, M.C.A., Cannon, S.C., and Papazian, D.M. (2018). Kv4.2 autism and epilepsy mutation enhances inactivation of closed channels but impairs access to inactivated state after opening *115*, E3559–E3568. https://doi.org/10.1073/pnas.1717082115.
43. Ågren, R., Geerdink, N., Brunner, H.G., Paucar, M., Kamsteeg, E.J., and Sahlholm, K. (2023). An E280K Missense Variant in *KCND3*/Kv4.3-Case Report and Functional Characterization. Int. J. Mol. Sci. *24*. https://doi.org/10.3390/ijms241310924.
44. Paucar, M., Ågren, R., Li, T., Lissmats, S., Bergendal, Å., Weinberg, J., Nilsson, D., Savichetva, I., Sahlholm, K., Nilsson, J., and Svenningsson, P. (2021). V374A *KCND3* Pathogenic Variant Associated With Paroxysmal Ataxia Exacerbations. Neurol. Genet. *7*, e546. https://doi.org/10.1212/NXG.0000000000000546.
45. Zanni, G., Hsiao, C.T., Fu, S.J., Tang, C.Y., Capuano, A., Bosco, L., Graziola, F., Bellacchio, E., Servidei, S., Primiano, G., et al. (2021). Novel *KCND3* variant underlying nonprogressive congenital ataxia or SCA19/22 disrupt Kv4.3 protein expression and K^+ currents with variable effects on channel properties. Int. J. Mol. Sci. *22*, 4986. https://doi.org/10.3390/ijms22094986.
46. Masnada, S., Hedrich, U.B.S., Gardella, E., Schubert, J., Kaiwar, C., Klee, E.W., Lanpher, B.C., Gavrilova, R.H., Synofzik, M., Bast, T., et al. (2017). Clinical spectrum and genotype-phenotype associations of *KCNA2*-related encephalopathies. Brain *140*, 2337–2354. https://doi.org/10.1093/brain/awx184.
47. Rinaldi, B., Bayat, A., Zachariassen, L.G., Sun, J.H., Ge, Y.H., Zhao, D., Bonde, K., Madsen, L.H., Awad, I.A.A., Bagiran, D., et al. (2023). Gain-of-function and loss-of-function variants in *GRIA3* lead to distinct neurodevelopmental phenotypes. Brain *147*, 1837–1855. https://doi.org/10.1093/brain/awad403.

48. Pescosolido, M.F., Stein, D.M., Schmidt, M., El Achkar, C.M., Sabbagh, M., Rogg, J.M., Tantravahi, U., McLean, R.L., Liu, J.S., Poduri, A., and Morrow, E.M. (2014). Genetic and phenotypic diversity of NHE6 mutations in Christianson syndrome. Ann. Neurol. *76*, 581–593. https://doi.org/10.1002/ana.24225.
49. Serôdio, P., and Rudy, B. (1998). Differential expression of Kv4 K+ channel subunits mediating subthreshold transient K^+ (A-type) currents in rat brain. J. Neurophysiol. *79*, 1081–1091. https://doi.org/10.1152/jn.1998.79.2.1081.
50. Chiu, C.Y., and Tsaur, M.L. (2022). K^+ channel Kv4.1 is expressed in the nociceptors/secondary nociceptive neurons and participates in pain regulation. Eur. J. Pain *26*, 2238–2256. https://doi.org/10.1002/ejp.2038.
51. Hermanstyne, T.O., Granados-Fuentes, D., Mellor, R.L., Herzog, E.D., and Nerbonne, J.M. (2017). Acute knockdown of Kv4.1 regulates repetitive firing rates and clock gene expression in the suprachiasmatic nucleus and daily rhythms in locomotor behavior. eNeuro *4*. https://doi.org/10.1523/ENEURO.0377-16.2017.
52. Kim, K.R., Lee, S.Y., Yoon, S.H., Kim, Y., Jeong, H.J., Lee, S., Suh, Y.H., Kang, J.S., Cho, H., Lee, S.H., et al. (2020). Kv4.1, a key ion channel for low frequency firing of dentate granule cells, is crucial for pattern separation. J. Neurosci. *40*, 2200–2214. https://doi.org/10.1523/JNEUROSCI.1541-19.2020.
53. Song, W.J., Tkatch, T., Baranauskas, G., Ichinohe, N., Kitai, S.T., and Surmeier, D.J. (1998). Somatodendritic depolarization-activated potassium currents in rat neostriatal cholinergic interneurons are predominantly of the A type and attributable to coexpression of Kv4.2 and Kv4.1 subunits. J. Neurosci. *18*, 3124–3137. https://doi.org/10.1523/JNEUROSCI.18-09-03124.1998.
54. Connor, J.A., and Stevens, C.F. (1971). Prediction of repetitive firing behaviour from voltage clamp data on an isolated neurone soma. J. Physiol. *213*, 31–53. https://doi.org/10.1113/jphysiol.1971.sp009366.
55. Jang, S.H., Choi, C., Hong, S.G., Yarishkin, O.V., Bae, Y.M., Kim, J.G., O'Grady, S.M., Yoon, K.A., Kang, K.S., Ryu, P.D., and Lee, S.Y. (2009). Silencing of Kv4.1 potassium channels inhibits cell proliferation of tumorigenic human mammary epithelial cells. Biochem. Biophys. Res. Commun. *384*, 180–186. https://doi.org/10.1016/j.bbrc.2009.04.108.
56. Kim, H.J., Jang, S.H., Jeong, Y.A., Ryu, P.D., Kim, D.Y., and Lee, S.Y. (2010). Involvement of Kv4.1 K^+ channels in gastric cancer cell proliferation. Biol. Pharm. Bull. *33*, 1754–1757. https://doi.org/10.1248/bpb.33.1754.
57. Shi, J., Miles, D.K., Orr, B.A., Massa, S.M., and Kernie, S.G. (2007). Injury-induced neurogenesis in Bax-deficient mice: evidence for regulation by voltage-gated potassium channels. Eur. J. Neurosci. *25*, 3499–3512. https://doi.org/10.1111/j.1460-9568.2007.05624.x.
58. Noda, M., Iwamoto, I., Tabata, H., Yamagata, T., Ito, H., and Nagata, K.I. (2019). Role of Per3, a circadian clock gene, in embryonic development of mouse cerebral cortex. Sci. Rep. *9*, 5874. https://doi.org/10.1038/s41598-019-42390-9.
59. Firth, H.V., Richards, S.M., Bevan, A.P., Clayton, S., Corpas, M., Rajan, D., Van Vooren, S., Moreau, Y., Pettett, R.M., and Carter, N.P. (2009). DECIPHER: Database of Chromosomal Imbalance and Phenotype in Humans Using Ensembl Resources. Am. J. Hum. Genet. *84*, 524–533. https://doi.org/10.1016/j.ajhg.2009.03.010.

The American Journal of Human Genetics, Volume *111*

Supplemental information

Etiological involvement of *KCND1* variants in an X-linked neurodevelopmental disorder with variable expressivity

Tassja Kalm, Claudia Schob, Hanna Völler, Thatjana Gardeitchik, Christian Gilissen, Rolph Pfundt, Chiara Klöckner, Konrad Platzer, Annick Klabunde-Cherwon, Markus Ries, Steffen Syrbe, Francesca Beccaria, Francesca Madia, Marcello Scala, Federico Zara, Floris Hofstede, Marleen E.H. Simon, Richard H. van Jaarsveld, Renske Oegema, Koen L.I. van Gassen, Sjoerd J.B. Holwerda, Tahsin Stefan Barakat, Arjan Bouman, Marjon van Slegtenhorst, Sara Álvarez, Alberto Fernández-Jaén, Javier Porta, Andrea Accogli, Margherita Maria Mancardi, Pasquale Striano, Michele Iacomino, Jong-Hee Chae, SeSong Jang, Soo Y. Kim, David Chitayat, Saadet Mercimek-Andrews, Christel Depienne, Antje Kampmeier, Alma Kuechler, Harald Surowy, Enrico Silvio Bertini, Francesca Clementina Radio, Cecilia Mancini, Simone Pizzi, Marco Tartaglia, Lucas Gauthier, David Genevieve, Mylène Tharreau, Noy Azoulay, Gal Zaks-Hoffer, Nesia K. Gilad, Naama Orenstein, Geneviève Bernard, Isabelle Thiffault, Jonas Denecke, Theresia Herget, Fanny Kortüm, Christian Kubisch, Robert Bähring, and Stefan Kindler

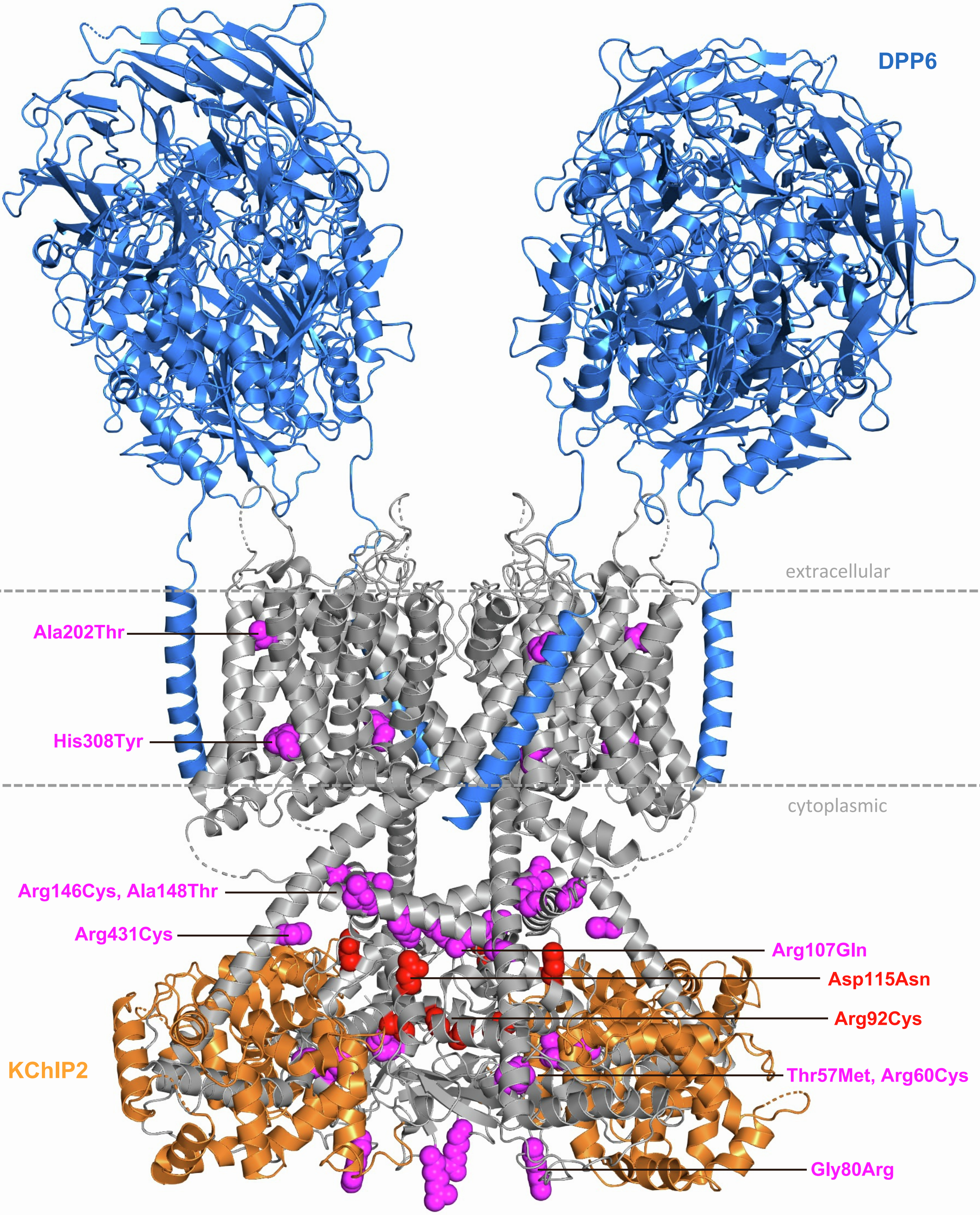

Figure S1. Structure of the ternary Kv4/KChIP/DPP channel complex

The structure of the Kv4.2/KChIP1/DPP6 complex reported by Kise and co-workers (PDB ID 7E8H)[1] was used to give an overview of the ternary channel configuration (α-subunits: grey, KChIPs: orange, DPPs: blue) and the location of *KCND1* variant-associated amino acid substitutions. Amino acid exchanges at sites homologous to Kv4.1 were simulated with the most likely backbone-dependent rotamer orientation using PyMOL (Schrödinger). Protein backbones depicted as ribbon diagrams. Substituted amino acid side chains depicted as red (group 1 variants) or purple (group 2 variants) CPK models. In each case one of the four corresponding substitutions is indicated and labeled (unlabeled structure also available as Movie S1). Note that p.Thr516Ser, p.Arg536Gly and p.Asn578Ile are absent because the structure lacks the C-terminal ends of the α-subunits. Horizontal dotted lines indicate the location of the plasma membrane separating extracellular from cytoplasmic space.

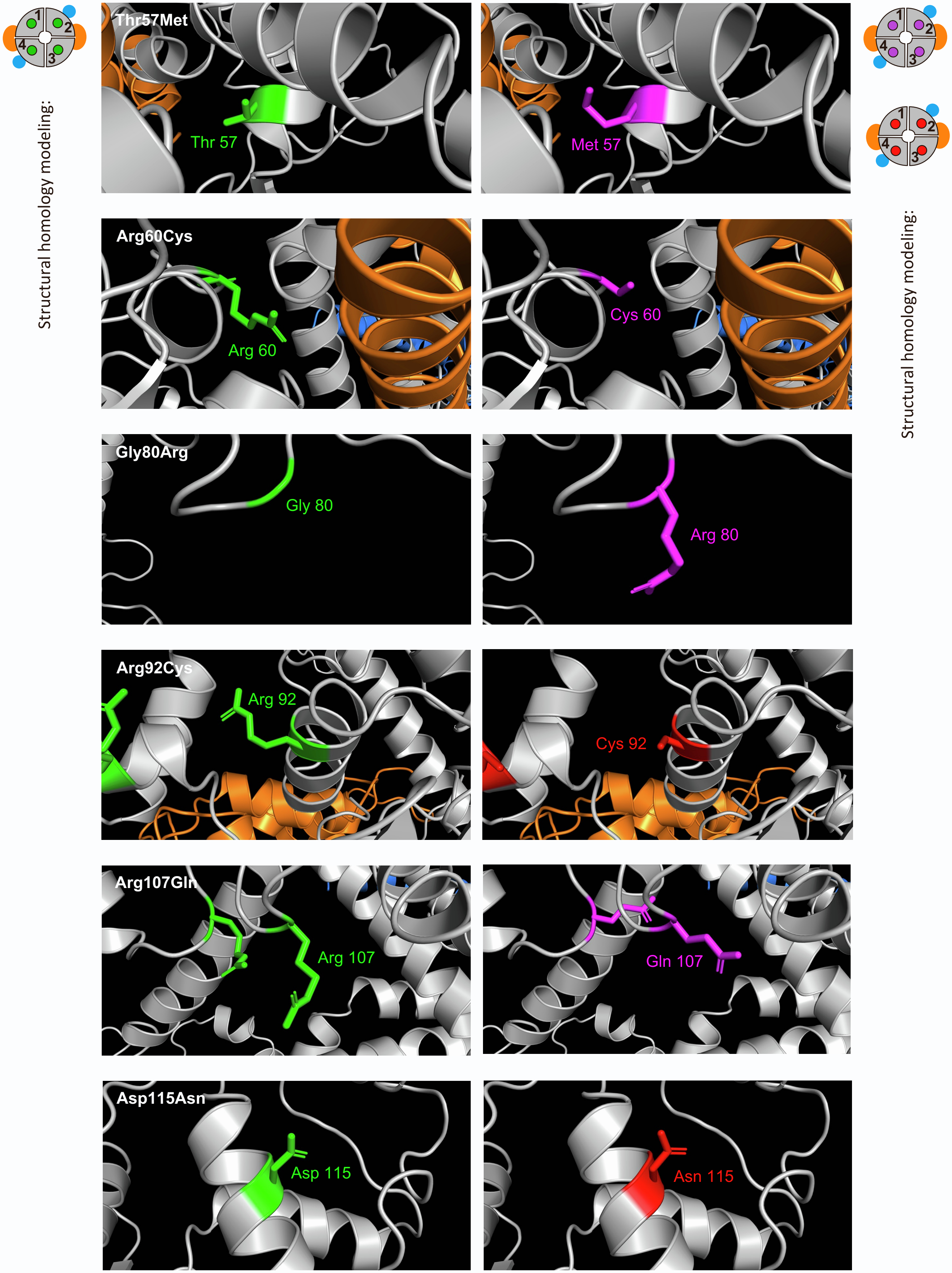

Structural homology modeling:
Thr57Met
Thr 57
Met 57
Arg60Cys
Arg 60
Cys 60
Gly80Arg
Gly 80
Arg 80
Arg92Cys
Arg 92
Cys 92
Arg107Gln
Arg 107
Gln 107
Asp115Asn
Asp 115
Asn 115
Structural homology modeling:

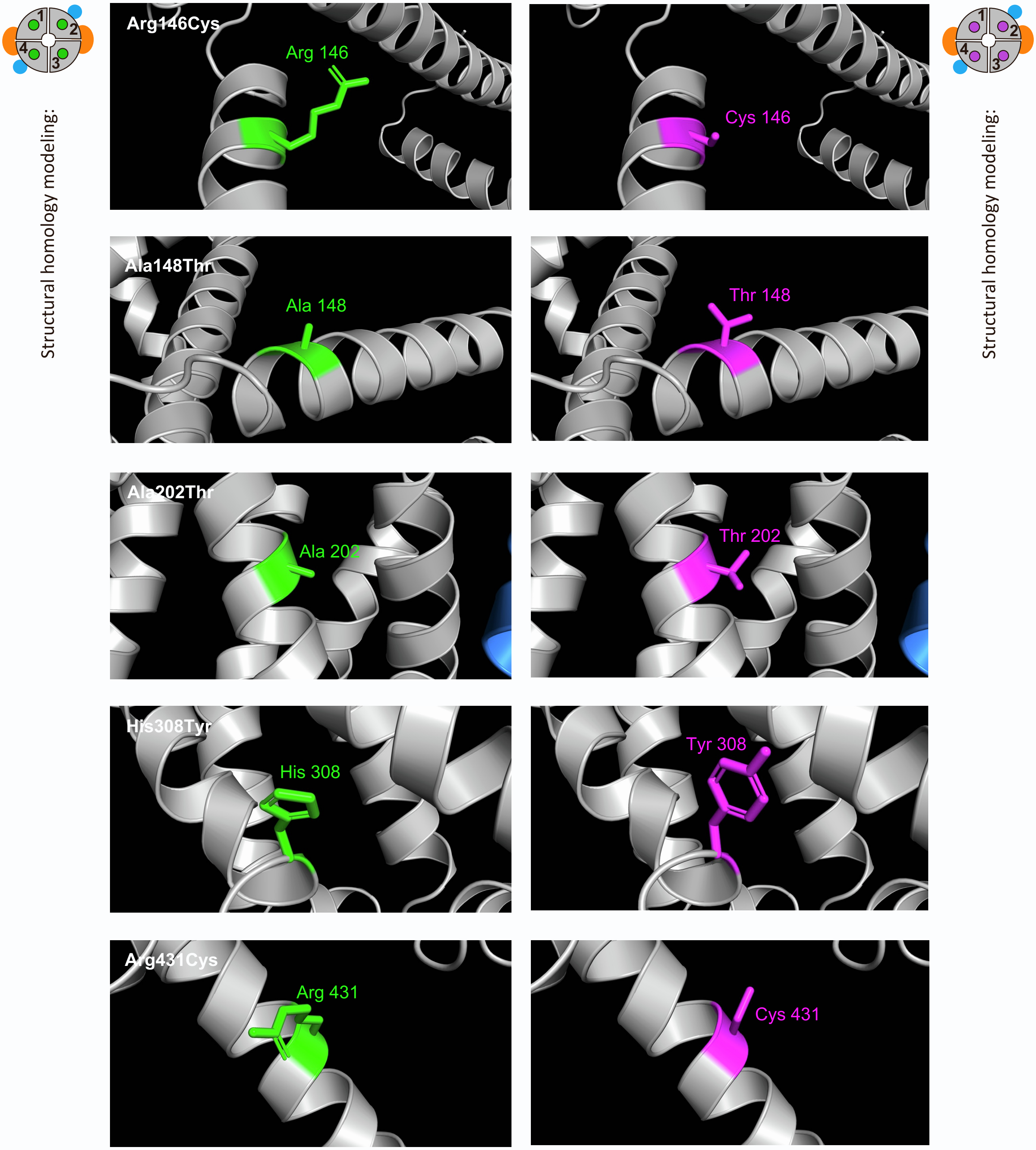

Figure S2. Structural side chain modification caused by *KCND1* variant-associated amino acid substitutions

Structural homology modeling using UniProt (https://www.uniprot.org/), SWISS MODEL (Center for Molecular Life Sciences, University of Basel, Switzerland; https://swissmodel.expasy.org/) and PyMOL (Schrödinger), was performed based on the amino acid sequences of human Kv4.1 (UniProt identifier: Q9NSA2), KChIP2 (UniProt identifier: Q9NS61) and DPP6 (UniProt identifer: P42658), and the ternary Kv4.2/KChIP1/DPP6 structure reported by Kise and co-workers (PDB ID 7E8H).[1] The structure provided by homology modeling was incomplete showing a 4:2:2 stoichiometry due to the lack of two opposite KChIP and DPP molecules (see cartoons). Computer-simulated amino acid substitutions using PyMOL (Schrödinger) were done with consideration of the most likely backbone-dependent rotamer orientation, and the putative steric consequences of the amino acid substitution within a distance of 8 Å were taken into account. Protein backbones depicted as ribbon diagrams. Native (wild-type) and novel variant-associated side chains depicted in stick mode, native residues in green, mutated residues in red (group 1 variants) or purple (group 2 variants).

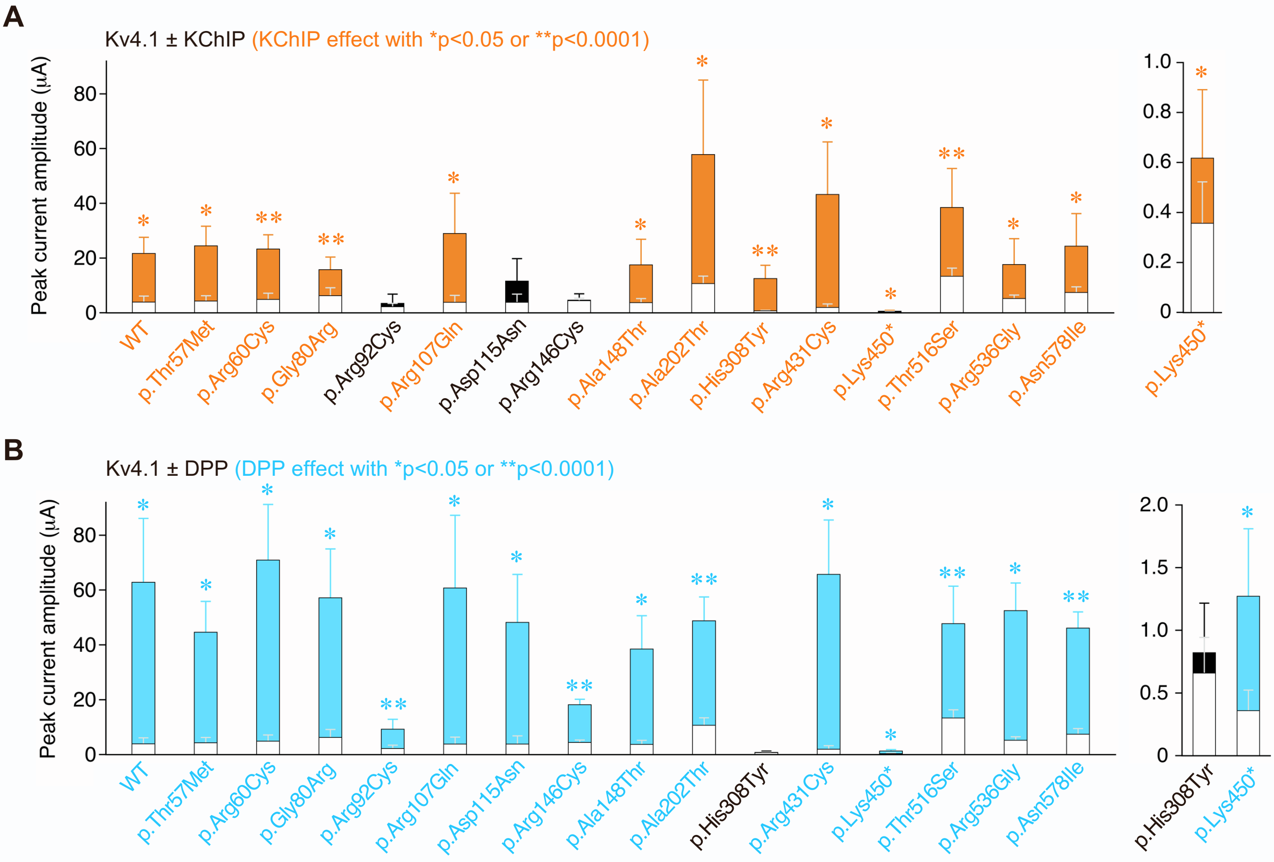

Figure S3. Effects of auxiliary β-subunit co-expression on Kv4.1 channel-mediated peak current amplitudes

Mean peak current amplitudes (data from Tables S4 – S6, error bars are SD) obtained in the absence (empty bars) and presence of either KChIP or DPP, as indicated by the filled bar extensions.

(A) Effects of KChIP co-expression; p.Lys450* data plotted on two different y-scales;

(B) Effects of DPP co-expression; p.His308Tyr and p.Lys450* data plotted on two different y-scales;

Statistics are based on unpaired Student's *t*-tests, applied to the data from Tables S4 – S6; significant effects of auxiliary β-subunit co-expression (compared to Kv4.1 alone) are indicated with * ($p<0.05$) or ** ($p<0.0001$) and by color (significant KChIP effect: orange; significant DPP effect: blue; absence of or non-significant effects: black). Note that the typical incease in peak current amplitude caused by both KChIP and DPP co-expression,[2,3] as seen for Kv4.1 WT and the majority of variants, is absent in the case of p.Arg92Cys, p.Asp115Asn and p.Arg146Cys for KChIP co-expression, and in the case of p.His308Tyr for DPP co-expression.

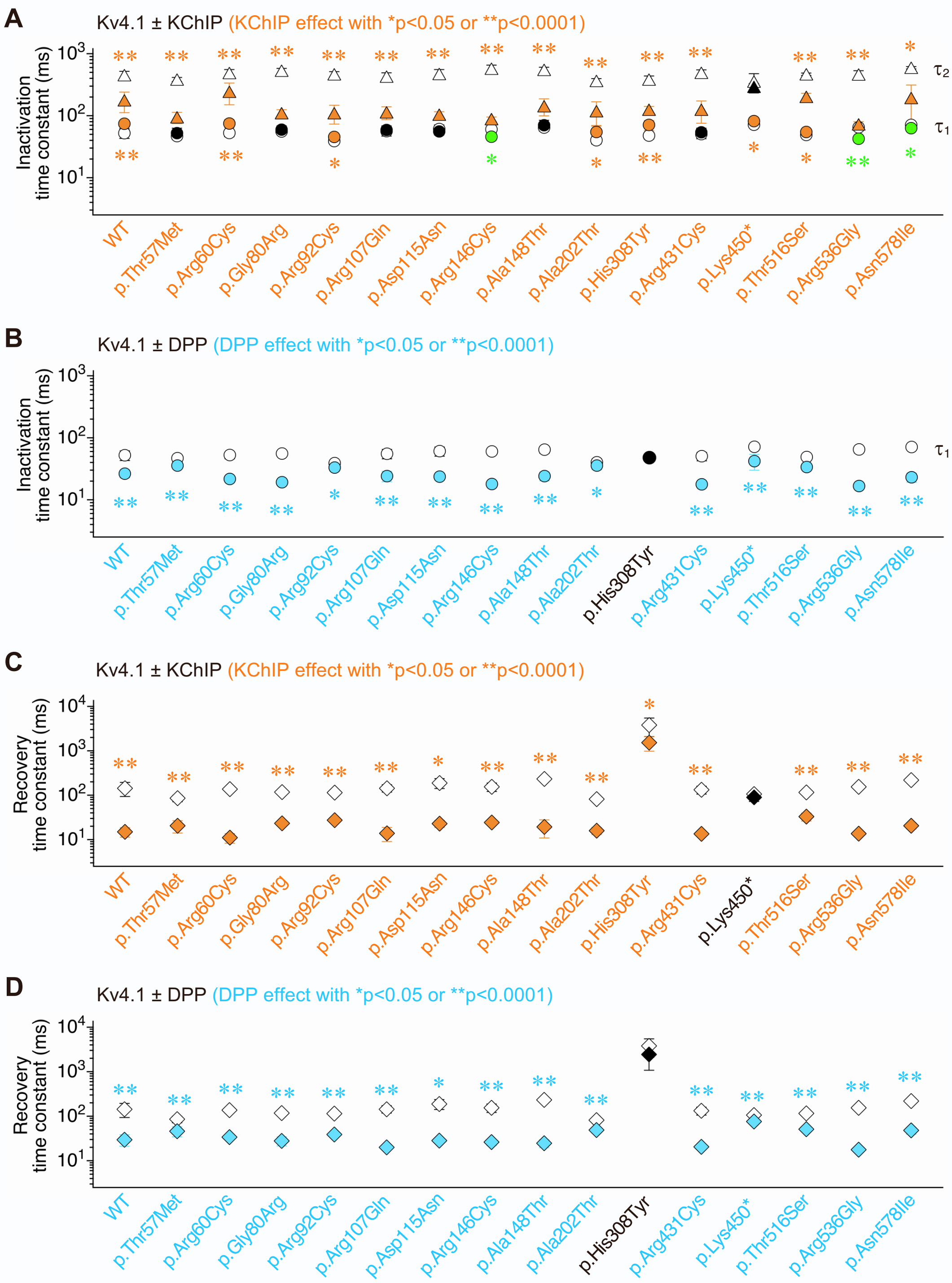

Figure S4. Effects of auxiliary β-subunit co-expression on Kv4.1 channel macroscopic inactivation and recovery kinetics

Mean values of inactivation and recovery time constants (data from Tables S4 – S6, error bars are SD and in many cases smaller than symbols) obtained in the absence (empty symbols) and presence of either KChIP or DPP (filled symbols).

(A and C) Effects of KChIP co-expression;

(B and D) Effects of DPP co-expression;

Statistics are based on unpaired Student's *t*-tests, applied to the data from Tables S4 – S6; significant effects of auxiliary β-subunit co-expression (compared to Kv4.1 alone) are indicated with * ($p<0.05$) or ** ($p<0.0001$) and by color (significant KChIP effect: orange; significant DPP effect: blue; absence of or non-significant effects: black). Typically, KChIP causes a slowing of the initial (fast) and an acceleration of the second (slower) current decay component (τ_1 and τ_2 depicted as circles and triangles, respectively), whereas DPP causes an acceleration of the initial (fast) current decay component (τ_1), while both KChIP and DPP speed up recovery from inactivation.[2,3] These typical modifications were seen for Kv4.1 WT and, at least partially, for the majority of variants. Still, for p.Lys450* KChIP failed to accelerate the recovery kinetics, and for p.His308Tyr none of the typical DPP effects was seen. Notably, in the case of p.Arg146Cys, p.Arg536Gly and p.Asn578Ile KChIP caused an accceleration of the initial current decay component (filled green symbols).

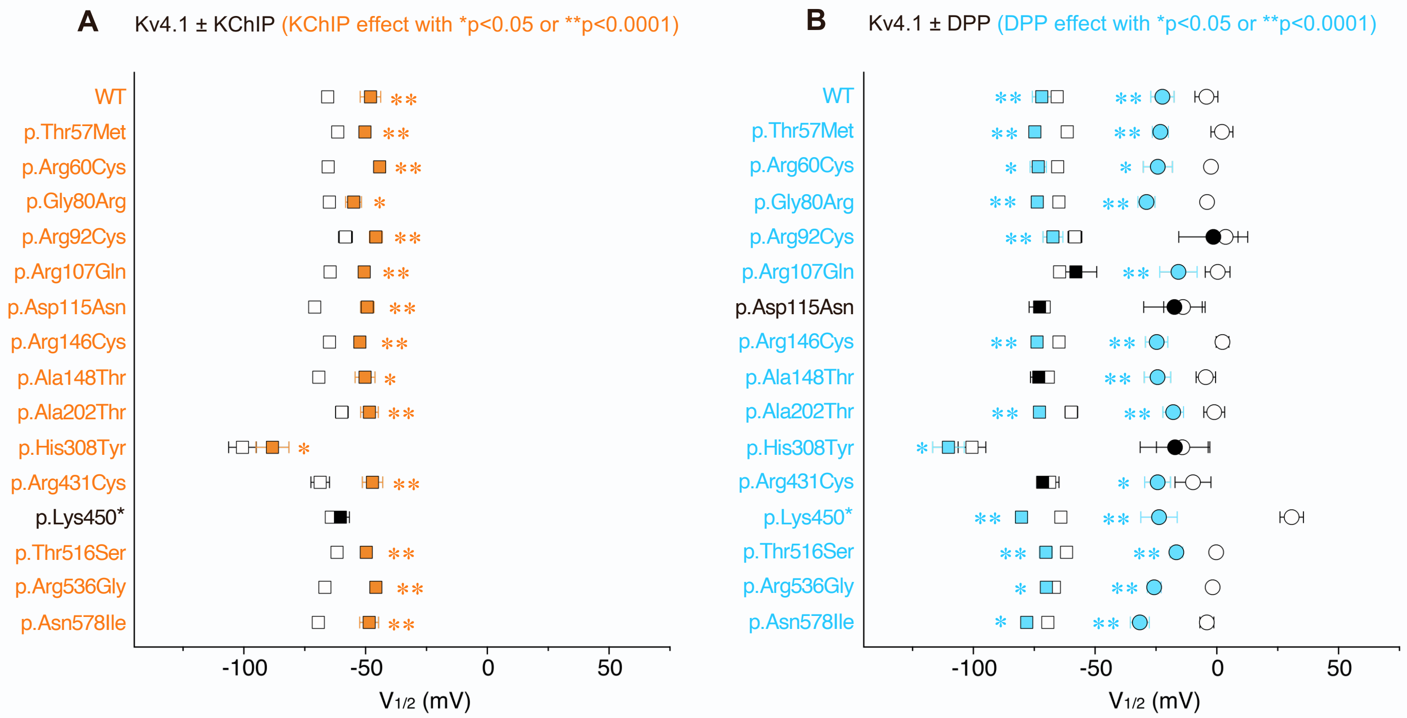

Figure S5. Effects of auxiliary β-subunit co-expression on the voltage dependences of Kv4.1 channel activation and steady-state inactivation

Mean values obtained for the voltages of halfmaximal activation and inactivation (data from Tables S4 – S6, error bars are SD and in many cases smaller than symbols) obtained in the absence (empty symbols) and in the presence of either KChIP or DPP (filled symbols).

(A) Effects of KChIP co-expression;

(B) Effects of DPP co-expression;

Statistics are based on unpaired Student's *t*-tests, applied to the data from Tables S4 – S6; significant effects of auxiliary β-subunit co-expression (compared to Kv4.1 alone) are indicated with * ($p<0.05$) or ** ($p<0.0001$) and by color (significant KChIP effect: orange; significant DPP effect: blue; absence of or non-significant effects: black). Typically KChIP causes a positive shift of the inactivation curve, and DPP a negative shift of both activation and inactivation curve,[2,3] as seen for Kv4.1 WT and, at least partially, for the majority of variants. However, in the case of p.Lys450* KChIP failed to significantly shift the inactivation curve in the positive direction, and in the case of p.Asp115Asn DPP failed to significantly shift both curves in the negative direction.

Individual #	1	2	3	4	5	6	7	8	9
Mutation (NM_004979.6; NP_004970.3)	c.274C>T (p.Arg92Cys)	c.343G>A (p.Asp115Asn)	c.182_194del (p.Tyr61Cysfs*31)	c.295C>T (p.Arg99*)	c.1348A>T (p.Lys450*)	c.170C>T (p.Thr57Met)	c.178C>T (p.Arg60Cys)	c.238G>C (p.Gly80Arg)	c.320G>A (p.Arg107Gln)
Origin	*de novo*	*de novo*	MI	MI	MI	MI	MI	MI	MI
Additional candidate variants	none	none	none	none	none	*TCEAL2*: c.595C>T (p.Gln199*), NM_080390.4, homozygous	none	*AREG*: c.440delA (p.Asn147Metfs*16), NM_001657.4, heterozygous	none
Birth weight (z, date of birth)	RN (40.5 wks)	ND (39.7 wks)	RN (at term)	2680 g (-0.79, 36.7 wks)	3262 g (-0.99, 40.7 wks)	4210 g (1.33, 40 wks)	3000 g (-1.25, 39.5 wks)	2100 g (-0.57, 34 wks)	2350 g (-2.2, 38 wks)
Birth length (z)	ND	ND	RN	ND	52 cm (-0.4)	ND	ND	ND	ND
OFC birth (z)	ND	ND	RN	ND	ND	ND	ND	ND	ND
Age last exam	15 y	9 y 11 m	16 y	16 y	3 y 2m	5 y 6 m	3 y 6 m	16 y	7 y
Weight last exam (z)	74 kg (1.1)	28.6 kg (-0.34)	ND	45.6 kg (-2.34)	15.5 kg (0.25)	23.5 kg (0.95)	15.9 kg (0.14)	46 kg (-2.28)	ND
Height last exam (z)	185.5 cm (1.54)	132.5 cm (-0.56)	ND	169 cm (-1.02)	96.4 cm (-0.5)	118 cm (0.58)	99.2 cm (-0.29)	166 cm (-1.42)	ND
OFC at last exam (z)	ND	55.5 cm (1.71)	ND	RN	50.7 cm (-0.09)	52 cm (0.07)	50.5 cm (-0.36)	54 cm (-1.57)	ND
Age at first signs	10 y	6 y	2 y 3 m	abt 2 y	neonatal period	abt 3 y	abt 2 y	3 y	4 y 10 m
First signs	anxiety, tics	speech delay, seizures	seizures	speech delay, motor delay, anxiety	speech delay, motor delay	speech delay, mild motor delay	speech delay mild motor delay	speech delay, autistic features	seizures (motor, with impaired awareness)
Motor development	normal	normal	normal	delayed	delayed	mildly delayed	mildly delayed	normal	normal
Intellectual development	average	low average, TIQ 85	average	borderline mental functioning – low average, TIQ 74-86	extremely low/impaired, TIQ 68	WNV of Bayley-III-NL could not be completed, developmental age estimate at 53 m of age: 20-42 m	extremely low/impaired, TIQ 55	extremely low/impaired, TIQ <50, WISC-IV 43 (7 y), WISC-V 41 (13 y)	average
Speech development	normal	delayed	normal	delayed	delayed	delayed	delayed	delayed, pragmatic language disorder	mildly delayed
Receptive language	normal	below average	normal	below average	below average	poor	ND	ND	ND
Comprehension	normal	below average	normal	below average	below average	below average	below average	below average	below average
Neuropsychiatric signs	ASD, attention deficit, anxiety, tics	attention deficit	none	ASD, anxiety, emotional problems	attention deficit	poor social skills, little contact, attention deficit	past temper tantrums	ASD, hyperactivity, attention deficit	hyperactivity, attention deficit
Ambulation/ gait	normal	normal	normal	normal	normal	clumsy gait[a]	normal	normal	normal
Neurological and muscular features	none	mild hypotonia	none	ND	none	none	probably mild hypotonia in infancy	hypotonia	none
Seizures/epilepsy	no	epilepsy, absences, myoclonus, eyelid myoclonia	epilepsy, generalized tonic-clonic seizures during sleep until 11 y, then seizures remitted, Valproate responsive	no	no	no	no	no	epilepsy, multiple daily seizures, psychomotor arrest, eye deviation
EEG	normal	irregular polyspikes and spike-waves, photoparoxysmal reaction	generalized abnormalities at 3 y 8 m, normal at 12 y 6 m	ND	ND	ND	ND	normal	irregular polyspikes and spike-waves
Cerebral MRI	normal	normal	normal	ND	ND	ND	ND	normal	ND

**Individual #**	10	11	12	13	14[b]	15[c]	16[c]	17	18
Mutation (NM_004979.6; NP_004970.3)	c.436C>T (p.Arg146Cys)	c.442G>A (p.Ala148Thr)	c.604G>A (p.Ala202Thr)	c.922C>T (p.His308Tyr)	c.1291C>T (p.Arg431Cys)	c.1546A>T (p.Thr516Ser)	c.1546A>T (p.Thr516Ser)	c.1606A>G (p.Arg536Gly)	c.1733A>T (p.Asn578Ile)
Origin	MI	MI	MI	MI	MI	MI	MI	MI	MI
Additional candidate variants	none	none	*EIF2B3*: c.172delG (p.Asp58Metfs*12), NM_020365.5, heterozygous; *SYNE1*: c.18891+2T>C, NM_182961.4, heterozygous	*HAGHL*: c.55G>A (p.Glu19Lys), NM_207112.2, heterozygous	none	none	none	none	*AMER1*: c.3112C>T (p.Pro1038Ser), NM_152424.4, hemizygous
Birth weight (z, date of birth)	ND (at term)	2300 g (-1.26, 37 wks)	3543 g (0.13, 39 wks)	2650 g (-1.81, 38.7 wks)	3300 g (-0.14, at term)	3470 g (-0.38, 40 wks)	2680 g (-1.86, 39 wks)	3180 g (-0.16, 37.7 wks)	2050 g (-2.37, 37 wks)
Birth length (z)	ND	ND	52 cm (0.04)	48 cm (-1.6)	51 cm (-0.14)	50 cm (-1.12)	46 cm (-2.57)	48 cm (-1.12)	46 cm (-1.75)
OFC birth (z)	ND	ND	35 cm (-0.23)	34 cm (-0.9)	35 cm (-0.01)	34.5 cm (-0.88)	34 cm (-1)	ND	31 cm (-2.27)
Age last exam	21 y	5 y	6 y 11 m	1 y 9 m	8 m	11 y 8 m	6 y 4 m	10 y 1 m	3 y
Weight last exam (z)	88 kg	18 kg (-0.49)	23.8 kg (-0.03)	13.7 kg (1.03)	5.6 kg (-2.98)	47.3 kg (0.78)	24.3 kg (0.51)	38.5 kg (0.63)	ND
Height last exam (z)	ND	110 cm (-0.36)	ND	90 cm (1.26)	65 cm (-2.16)	158 cm (1.05)	125 cm (0.82)	138.5 cm (-0.52)	ND
OFC at last exam (z)	ND	50 cm (-1.3)	45.5 cm (-5.38)	48.5 cm (-0.47)	40 cm (-4.37)	54.3 cm (-0.02)	52.5 cm (0.16)	54.2 cm (0.4)	ND
Age at first signs	abt 3 y	1 y 6 m	3 m	2 m	at birth	1 y 8 m	5-6 m	neonatal period	at birth (prenatal)
First signs	speech delay, ID	speech delay, poor eye contact	seizures	seizures	hypotonia	speech delay	feeding issues	feeding issues	IUGR, PAPP-A, postnatal SGA, pneumothorax
Motor development	normal	normal	delayed	normal	delayed	normal	normal	delayed	delayed
Intellectual development	extremely low/impaired, FSIQ 40-50	average, WPPSI-IV 90	impaired, not formally examined due to severe developmental delay	average	impaired, not formally examined, severe developmental delay and early death	borderline mental functioning, FSIQ 77	borderline mental functioning, FSIQ 77	low average, TIQ 81[d]	impaired, not formally examined
Speech development	delayed	delayed	delayed, never acquired ability to speak	normal	ND	delayed	delayed, never acquired ability to speak	delayed	delayed
Receptive language	ND	below average	poor	normal	ND	below average	poor	normal	poor
Comprehension	below average	below average	below average	normal	ND	below average	below average	normal	below average
Neuropsychiatric signs	none	none	ND	none	ND	none	ASD	ASD, tics	ASD
Ambulation/ gait	normal	normal	never acquired ambulation	normal	ND	normal	normal	initially normal[e]	unsteady gait
Neurological and muscular features	none	none	generalized hypotonia	none	hypotonia	none	none	slight hypotonia	hypotonia (limbs)
Seizures/epilepsy	epilepsy, generalized tonic-clonic seizures, Oxcarbazepine & Levetiracetam responsive	no	epilepsy, frequent recurrent seizures, nonresponsive to multiple anti-epileptic drugs	epilepsy, generalized tonic-clonic seizures, frequent recurrent febrile and non-febrile seizures, no medication given	no	no	no	no	no
EEG	few low amplitude spike discharges on left centro-temporal area	normal	abnormal background with intermittent epileptiform discharges mainly in bifrontal regions	normal	normal	ND	ND	normal	normal
Cerebral MRI	normal	normal	corpus callosum absent, progressive cerebral atrophy with relative sparing of basal ganglia and cerebellum, myelination arrest at 5 m	unremarkable at 2 m, enlarged external ventricles supratentorial	hypomyelination, abnormal symmetrical central tegmental tract hyper-intensities, increased DWI signal of bilateral central tegmental tract	hypoplasia of corpus callosum	small corpus callosum (3rd centile)	ND	bilateral small heterotopia in centrum semiovale

Table S1. Key clinical features of subjects with hemizygous *KCND1* variants (group 1 and group 2)

Z-scores for birth and growth parameters were calculated using the Ped(z) Pediatric Calculator (https://www.pedz.de/en/welcome.html). Abbreviations: abt, about; ASD, autism spectrum disorder; DD, developmental delay; exam, examination; DWI, diffusion-weighted magnetic resonance imaging; FSIQ, full scale intelligence quotient; ID, intellectual disability; IUGR, intrauterine growth retardation; m, months; MI, maternally inherited; ND, no data; OFC, occipitofrontal head circumference; PAPP-A, pregnancy-associated plasma protein A; RN, reported normal; SD, standard deviation; SGA, small for gestational age; TIQ, total intelligence quotient; WISC, Wechsler intelligence scale for children; WNV, Wechsler nonverbal scale of ability; wks, weeks; WPPSI, Wechsler preschool and primary scale of intelligence; y, years; z, z-score. [a]No clear evidence of ataxia, no broad-based gait, no coordination problems, no muscle weakness or hypertonia, but rather a somewhat unstable gait pattern possibly reflecting hypermobility or a general gross motor delay, [b]Died at eight months due to respiratory insufficiency, probably linked to progressive hypo-myelinating encephalopathy, [c]Brothers of different age, [d]At ten years of age, decline probably due to suspected childhood disintegrative disorder, [e]Increasingly frequent use of a wheel chair since the onset of suspected childhood disintegrative disorder

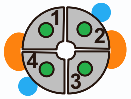

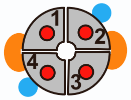

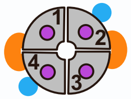

Structural homology modeling:

KCND1	side chain → side chain	side chain → backbone	backbone → side chain
Thr57	Thr53 (4-4-3.1Å)	Trp54 (4-4-2.9Å)	—
Met57	—	—	—
Arg60	Asn56 (1-1-2.8Å)	Gly4 (1-1-3.1Å); Asn56 (4-4-2.8Å)	—
Cys60	—	—	—
Gly80	—	—	—
Arg80	—	—	—
Arg92	Asp87 (1-4-2.6Å, 4-3-2.8Å); Asp89 (1-1-2.7Å, 4-4-2.6Å)	—	Asn96 (1-1-3.0Å, 4-4-3.3Å)
Cys92	—	—	Asn96 (1-1-2.9Å, 4-4-3.3Å)
Arg107	Gln108 (1-3-2.9Å)	Arg107 (1-4-3.5Å, 4-3-2.7Å); Glu109 (4-3-2.9Å)	Arg107 (1-2-2.7Å)
Gln107	Arg139 (1-4-2.9Å,3.1Å)	—	Arg139 (4-4-3.3Å)
Asp115	Tyr136 (4-4-2.6Å)	—	—
Asn115	Tyr136 (4-4-2.8Å); Lys140 (1-1-2.6Å; 4-4-3.0Å)	Asn115 (1-1-2.8Å)	—
Arg146	Glu134 (1-2-2.7Å); Asp130 (4-1-2.7Å); Glu142 (4-4-2.7Å)	Asp130 (4-1-3.0Å)	Arg424 (4-4-2.9Å)
Cys146	—	—	Arg424 (4-4-2.9Å)
Ala148	—	—	—
Thr148	—	Ala144 (1-1-3.0Å, 4-4-2.8Å)	—
Ala202	—	—	—
Thr202	—	Val198 (1-1-2.7Å, 4-4-2.8Å)	—
His308	—	Tyr189 (1-1-2.8Å); His308 (1-1-3.0Å)	—
Tyr308	—	—	—
Arg431	Gln428 (4-4-2.7Å)	Glu126 (1-2-3.3Å)	—
Cys431	—	—	—

Table S2. Polar contacts involving side chains in a modeled Kv4.1/KChIP2/DPP6 structure

A computer search for polar interactions involving side chains was performed on the Kv4.1/KChIP2/DPP6 structure provided by homology modeling (see cartoons: 4 α-subunits, grey; 2 KChIPs, orange; 2 DPPs, blue; see also Figure S2). Amino acid substitutions were modeled and searches for polar side chain interactions including distance measurements were performed for wild-type and variant α-subunits. The results obtained for the residues of interest (wild-type: green; group 1 mutants: red; group 2 mutants: purple) in α-subunits 1 and 4 are illustrated. Numbers separated by dashes represent (in this order): subunit of the residue under study (1 or 4) – subunit of the interaction partner – smallest distance between interacting atoms. All three possible combinations of side chain and backbone involvement are considered. Note that in the majority of cases the amino acid substitution leads to a reduction in the number of contacts.

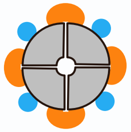

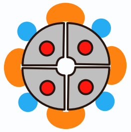

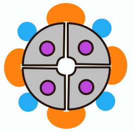

Heterologous expression:

Kv4.1 + KChIP + DPP	I_{+40} (µA)	τ_1 (ms)	τ_2 (ms)	% τ_1	τ_{rec} (ms)	$V_{1/2,inact}$ (mV)	k_{inact} (mV)	$V_{1/2,act}$ (mV)	k_{act} (mV)
WT	19.2 ± 9.5 (n = 16)	30.7 ± 4.7	85.8 ± 56.8	82.6 ± 10.8 (n = 21)	11.2 ± 2.0 (n = 16)	-57.9 ± 4.3	7.42 ± 2.00 (n = 18)	-6.5 ± 5.4	25.2 ± 2.2 (n = 17)
p.Thr57Met	17.6 ± 13.6 (n = 9)	26.8 ± 2.5	107 ± 36	93.6 ± 3.8 (n = 9)	10.6 ± 1.0 (n = 9)	-57.0 ± 2.8	7.32 ± 1.40 (n = 9)	-2.5 ± 2.7	24.2 ± 1.4 (n = 9)
p.Arg60Cys	23.2 ± 13.3 (n = 14)	27.1 ± 5.2	102 ± 49	91.7 ± 5.9 (n = 14)	9.80 ± 0.45 (n = 10)	-56.2 ± 2.5	7.31 ± 0.96 (n = 9)	-1.1 ± 3.6	28.5 ± 2.3 (n = 9)
p.Gly80Arg	15.8 ± 6.6 (n = 10)	25.4 ± 3.5	55.4 ± 22.1	76.2 ± 15.7 (n = 10)	9.74 ± 1.43 (n = 8)	-60.0 ± 3.0	7.23 ± 2.10 (n = 9)	-9.9 ± 3.4	24.7 ± 2.8 (n = 10)
pArg92Cys	5.47 ± 3.63 ** (n = 21)	36.3 ± 4.3 *	150 ± 83 *	83.3 ± 15.2 (n = 20)	12.2 ± 1.4 (n = 19)	-57.2 ± 2.5	6.49 ± 0.88 (n = 16)	-6.0 ± 5.3	21.5 ± 1.8 * (n = 12)
p.Arg107Gln	22.4 ± 7.7 (n = 13)	21.2 ± 2.4 **	54.5 ± 18.7	82.8 ± 16.6 (n = 13)	10.1 ± 3.3 (n = 8)	-57.1 ± 3.0	6.87 ± 1.53 (n = 9)	-4.8 ± 3.6	26.3 ± 1.9 (n = 8)
p.Asp115Asn	9.22 ± 4.36 * (n = 11)	18.4 ± 2.8 **	35.8 ± 5.5 *	70.1 ± 17.4 (n = 11)	11.3 ± 2.5 (n = 8)	-61.7 ± 2.3	6.32 ± 0.42 (n = 8)	-6.8 ± 2.4	24.3 ± 2.6 (n = 8)
p.Arg146Cys	4.22 ± 1.62 * (n = 10)	21.0 ± 2.4 **	40.9 ± 8.1	65.4 ± 13.6 * (n = 10)	11.5 ± 1.6 (n = 8)	-61.8 ± 2.3	8.48 ± 0.76 (n = 6)	-0.5 ± 7.6	24.2 ± 3.7 (n = 7)
p.Ala148Thr	12.5 ± 3.1 (n = 10)	26.0 ± 2.3	47.1 ± 6.0	63.7 ± 10.8 * (n = 10)	12.4 ± 1.4 (n = 8)	-61.2 ± 4.2	7.14 ± 0.60 (n = 8)	-8.2 ± 4.8	24.5 ± 2.5 (n = 8)
p.Ala202Thr	20.8 ± 12.2 (n = 13)	33.5 ± 5.8	82.0 ± 19.3	81.0 ± 12.0 (n = 13)	11.0 ± 1.5 (n = 9)	-53.3 ± 5.3 *	10.7 ± 3.6 ** (n = 9)	+3.1 ± 6.2 *	27.6 ± 2.7 (n = 10)
p.His308Tyr	2.30 ± 1.95 ** (n = 12)	57.0 ± 13.1 **	116 ± 35	41.2 ± 27.9 ** (n = 12)	937 ± 293 ** (n = 9)	-98.6 ± 6.5 **	7.79 ± 2.80 (n = 9)	-22.9 ± 17.2 **	33.2 ± 8.4 ** (n = 9)
p.Arg431Cys	18.2 ± 9.8 (n = 10)	29.6 ± 2.4	75.8 ± 14.9	83.2 ± 10.0 (n = 9)	11.5 ± 1.4 (n = 9)	-59.7 ± 5.2	6.85 ± 1.03 (n = 8)	-7.7 ± 5.6	25.7 ± 2.9 (n = 9)
p.Lys450*	1.20 ± 0.75 ** (n = 12)	35.8 ± 5.7 *	156 ± 30 **	60.8 ± 7.6 ** (n = 15)	38.7 ± 4.5 (n = 9)	-67.7 ± 3.1 **	7.17 ± 1.24 (n = 7)	-10.7 ± 6.9	24.4 ± 3.8 (n = 7)
p.Thr516Ser	19.1 ± 8.2 (n = 15)	46.1 ± 4.3 **	146 ± 17 *	72.8 ± 3.7 (n = 14)	18.7 ± 2.5 (n = 13)	-46.0 ± 2.0 **	6.70 ± 1.16 (n = 11)	+4.4 ± 5.3 *	23.1 ± 3.1 (n = 12)
p.Arg536Gly	17.6 ± 9.6 (n = 12)	32.1 ± 4.0	88.4 ± 45.5	86.0 ± 10.6 (n = 12)	10.7 ± 1.3 (n = 9)	-59.2 ± 2.9	7.24 ± 1.18 (n = 9)	-11.6 ± 3.2	25.1 ± 0.9 (n = 9)
p.Asn578Ile	21.9 ± 9.8 (n = 12)	38.5 ± 5.1 *	125 ± 78	88.5 ± 11.0 (n = 12)	11.7 ± 2.4 (n = 9)	-58.6 ± 2.7	8.62 ± 2.87 (n = 8)	-14.6 ± 4.3	27.5 ± 2.1 (n = 7)

Table S3. Biophysical parameters for Kv4.1 wild-type and variant ternary channels

Kv4.1 channels (grey, variant channels with red or purple dots) were studied in a ternary configuration; i.e. in the presence of both KChIP (orange) and DPP (blue). Mean values ± SD and number of observations (n) are given for the peak current amplitude at + 40 mV (I_{+40}), the time constants of macroscopic inactivation (τ_1 and τ_2) including the fractional amplitude of τ_1 (%), the time constant of recovery from inactivation (τ_{rec}) and the voltages of halfmaximal inactivation ($V_{1/2,inact}$) and activation ($V_{1/2,act}$) with corresponding slope factors (k_{inact} and k_{act}, respectively). Statistics are based on one-way analysis of variance (ANOVA) with Dunnett's post hoc testing. For significant variant effects (i.e., deviations from Kv4.1 WT ternary; key variants: red, maternally inherited missense variants: purple) significance levels are indicated with * (p<0.05) or ** (p<0.0001); shaded fields: no significant differences found compared to Kv4.1 WT ternary.

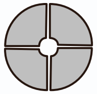

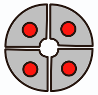

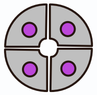

Heterologous expression:

Kv4.1	I_{+40} (µA)	τ_1 (ms)	τ_2 (ms)	% τ_1	τ_{rec} (ms)	$V_{1/2,inact}$ (mV)	k_{inact} (mV)	$V_{1/2,act}$ (mV)	k_{act} (mV)
WT	3.94 ± 2.24 (n = 46)	52.1 ± 9.3	452 ± 67	67.3 ± 5.6 (n = 45)	143 ± 50 (n = 22)	-65.6 ± 2.3	7.10 ± 1.71 (n = 24)	-4.3 ± 4.7	22.3 ± 2.1 (n = 25)
p.Thr57Met	4.25 ± 2.02 (n = 13)	46.9 ± 6.0	382 ± 29 *	72.3 ± 1.7 * (n = 13)	85.3 ± 14.2 (n = 8)	-61.5 ± 2.0 *	7.72 ± 1.53 (n = 8)	+2.1 ± 4.5 *	21.5 ± 2.8 (n = 7)
p.Arg60Cys	4.91 ± 2.26 (n = 17)	52.5 ± 6.5	487 ± 65	67.8 ± 5.3 (n = 16)	137 ± 18 (n = 8)	-65.4 ± 0.9	7.60 ± 2.05 (n = 9)	-2.4 ± 1.9	23.7 ± 0.7 (n = 6)
p.Gly80Arg	6.29 ± 2.90 * (n = 16)	55.4 ± 5.6	528 ± 51 *	64.6 ± 3.6 (n = 20)	117 ± 13 (n = 9)	-64.9 ± 1.2	9.04 ± 2.72 * (n = 10)	-4.0 ± 1.8	24.1 ± 0.7 (n = 4)
p.Arg92Cys	2.20 ± 1.28 * (n = 26)	38.7 ± 1.8 **	467 ± 45	72.9 ± 1.3 * (n = 17)	114 ± 13 (n = 11)	-58.3 ± 2.8 **	6.11 ± 0.35 (n = 10)	+3.6 ± 5.1 *	22.0 ± 2.0 (n = 11)
p.Arg107Gln	3.80 ± 2.56 (n = 66)	56.4 ± 9.7	424 ± 64	67.4 ± 5.1 (n = 39)	144 ± 27 (n = 11)	-64.5 ± 2.3	8.65 ± 1.72 (n = 16)	+0.4 ± 5.1	23.9 ± 2.9 (n = 13)
p.Asp115Asn	3.86 ± 2.95 (n = 33)	60.5 ± 10.1 **	469 ± 85	68.5 ± 5.2 (n = 31)	188 ± 47 (n = 5)	-70.9 ± 1.3 **	9.85 ± 1.52 * (n = 8)	-13.9 ± 7.9 *	20.8 ± 4.2 (n = 4)
p.Arg146Cys	4.46 ± 0.92 (n = 12)	60.0 ± 6.9 *	573 ± 81 **	72.4 ± 3.7 * (n = 20)	155 ± 33 (n = 9)	-64.9 ± 1.3	10.7 ± 3.0 ** (n = 9)	+2.3 ± 2.6	26.5 ± 2.4 * (n = 5)
p.Ala148Thr	3.70 ± 1.53 (n = 19)	64.2 ± 7.7 **	546 ± 57 **	69.0 ± 4.4 (n = 19)	231 ± 19 (n = 8)	-69.2 ± 1.0 *	6.85 ± 0.33 (n = 8)	-4.5 ± 4.0	23.3 ± 1.8 (n = 10)
p.Ala202Thr	10.7 ± 2.72 ** (n = 12)	40.3 ± 5.3 **	361 ± 35 *	73.8 ± 2.7 * (n = 12)	81.9 ± 9.8 (n = 7)	-59.8 ± 2.6 **	6.77 ± 0.83 (n = 9)	-1.1 ± 4.3	21.2 ± 1.1 (n = 9)
p.His308Tyr	0.66 ± 0.28 ** (n = 12)	47.9 ± 5.9	385 ± 51 *	65.6 ± 4.7 (n = 12)	3760 ± 1650 ** (n = 10)	-100.6 ± 5.7 **	7.16 ± 2.32 (n = 8)	-14.2 ± 10.6 *	24.8 ± 5.3 (n = 7)
p.Arg431Cys	1.98 ± 1.23 * (n = 16)	50.3 ± 8.0	492 ± 56	67.7 ± 3.9 (n = 25)	132 ± 27 (n = 9)	-68.6 ± 3.8 *	9.21 ± 1.76 * (n = 8)	-9.8 ± 7.4	22.7 ± 2.3 (n = 4)
p.Lys450*	0.36 ± 0.17 ** (n = 19)	71.9 ± 6.5 **	354 ± 123 **	73.9 ± 6.0 * (n = 12)	106 ± 7 (n = 5)	-64.1 ± 1.8	9.43 ± 0.62 (n = 4)	+30.7 ± 4.8 **	34.1 ± 4.0 ** (n = 5)
p.Thr516Ser	13.3 ± 3.07 ** (n = 20)	48.8 ± 3.9	464 ± 20	68.7 ± 1.8 (n = 20)	115 ± 8 (n = 6)	-61.7 ± 1.9 *	7.18 ± 0.28 (n = 5)	-0.3 ± 1.9	22.4 ± 0.8 (n = 5)
p.Arg536Gly	5.17 ± 1.33 (n = 15)	64.8 ± 7.8 **	460 ± 73	64.5 ± 4.3 (n = 22)	153 ± 16 (n = 10)	-66.7 ± 2.3	8.29 ± 1.12 (n = 12)	-1.7 ± 1.9	23.2 ± 2.2 (n = 8)
p.Asn578Ile	7.39 ± 2.08 * (n = 5)	70.8 ± 3.3 **	591 ± 26 *	60.6 ± 1.3 * (n = 5)	219 ± 25 (n = 5)	-69.4 ± 1.4 *	8.01 ± 0.90 (n = 4)	-4.1 ± 2.9	24.5 ± 0.5 (n = 4)

Table S4. Biophysical parameters for Kv4.1 wild-type and variant homotetrameric channels

Kv4.1 channels (grey, variant channels with red or purple dots) were expressed in the absence of auxiliary β-subunits. Mean values ± SD and number of observations (n) are given for the peak current amplitude at + 40 mV (I_{+40}), the time constants of macroscopic inactivation (τ_1 and τ_2) including the fractional amplitude of τ_1 (%), the time constant of recovery from inactivation (τ_{rec}) and the voltages of halfmaximal inactivation ($V_{1/2,inact}$) and activation ($V_{1/2,act}$) with corresponding slope factors (k_{inact} and k_{act}, respectively); Statistics are based on one-way analysis of variance (ANOVA) with Dunnett's post hoc testing. For significant variant effects (i.e., deviations from Kv4.1 WT; key variants: red, maternally inherited missense variants: purple) significance levels are indicated with * ($p<0.05$) or ** ($p<0.0001$); shaded fields: no significant differences found compared to Kv4.1 WT.

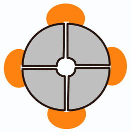

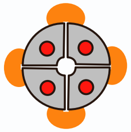

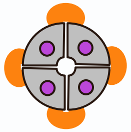

Heterologous expression:

Kv4.1 + KChIP	I_{+40} (μA)	τ_1 (ms)	τ_2 (ms)	% τ_1	τ_{rec} (ms)	$V_{1/2,inact}$ (mV)	k_{inact} (mV)	$V_{1/2,act}$ (mV)	k_{act} (mV)
WT	21.7 ± 6.0 (n = 7)	73.9 ± 8.1	176 ± 64	90.5 ± 6.6 (n = 6)	14.9 ± 3.5 (n = 5)	-48.0 ± 4.2	5.47 ± 1.28 (n = 5)	-7.0 ± 3.2	25.1 ± 2.1 (n = 6)
p.Thr57Met	24.4 ± 7.2 (n = 6)	52.1 ± 10.7 *	93.8 ± 19.4 *	55.8 ± 13.6 ** (n = 13)	20.4 ± 6.3 (n = 7)	-50.3 ± 2.0	5.82 ± 0.61 (n = 7)	-1.9 ± 3.4	20.8 ± 1.8 (n = 10)
p.Arg60Cys	23.3 ± 5.2 (n = 8)	74.7 ± 11.2	243 ± 93	93.2 ± 4.6 (n = 7)	11.0 ± 2.7 (n = 4)	-44.2 ± 1.6	6.76 ± 1.51 (n = 5)	-2.4 ± 2.8	26.9 ± 2.0 (n = 4)
p.Gly80Arg	15.7 ± 4.7 (n = 6)	59.3 ± 8.8	109 ± 15	49.6 ± 8.1 ** (n = 6)	23.1 ± 1.6 (n = 5)	-55.0 ± 3.2 *	4.44 ± 0.28 (n = 5)	-11.3 ± 2.8	20.1 ± 1.0 (n = 4)
p.Arg92Cys	3.50 ± 3.30 * (n = 11)	45.2 ± 4.8 **	110 ± 37 *	66.0 ± 16.1 * (n = 10)	27.3 ± 2.7 (n = 9)	-45.8 ± 2.6	6.69 ± 0.93 (n = 8)	+11.1 ± 10.0 **	24.7 ± 3.5 (n = 7)
p.Arg107Gln	29.0 ± 14.7 (n = 6)	59.1 ± 9.5	113 ± 26	79.4 ± 12.5 (n = 6)	13.9 ± 4.9 (n = 5)	-50.6 ± 1.7	4.94 ± 0.77 (n = 5)	-6.1 ± 1.3	24.5 ± 1.9 (n = 5)
p.Asp115Asn	11.6 ± 8.3 (n = 6)	55.5 ± 6.8 *	104 ± 12 *	69.0 ± 16.8 (n = 6)	23.0 ± 4.2 (n = 5)	-49.3 ± 2.8	8.32 ± 2.70 * (n = 6)	+3.1 ± 9.4 *	24.4 ± 4.1 (n = 6)
p.Arg146Cys	4.63 ± 2.32 * (n = 6)	45.7 ± 6.1 **	87.6 ± 7.3 *	39.2 ± 11.2 ** (n = 6)	24.3 ± 2.9 (n = 5)	-52.4 ± 1.3	6.48 ± 1.07 (n = 4)	-1.3 ± 2.4	21.3 ± 2.2 (n = 4)
p.Ala148Thr	17.5 ± 9.4 (n = 6)	70.3 ± 13.9	143 ± 44	77.1 ± 25.1 (n = 6)	19.4 ± 8.5 (n = 5)	-50.2 ± 4.1	4.59 ± 0.31 (n = 5)	-6.8 ± 6.2	21.0 ± 4.3 (n = 5)
p.Ala202Thr	57.9 ± 27.2 ** (n = 6)	54.7 ± 12.7 *	118 ± 49	66.9 ± 19.2 * (n = 9)	16.0 ± 2.9 (n = 8)	-48.4 ± 3.6	4.43 ± 0.42 (n = 8)	-1.9 ± 2.3	21.5 ± 2.0 (n = 9)
p.His308Tyr	12.5 ± 4.9 (n = 11)	70.8 ± 13.0	124 ± 16	41.4 ± 20.4 ** (n = 25)	1520 ± 540 ** (n = 11)	-88.3 ± 6.8 **	6.28 ± 3.34 (n = 9)	-30.5 ± 8.8 **	30.7 ± 7.7 * (n = 13)
p.Arg431Cys	43.3 ± 19.2 * (n = 8)	54.1 ± 8.6 *	124 ± 49	82.5 ± 18.4 (n = 8)	13.5 ± 1.5 (n = 5)	-47.2 ± 4.2	5.65 ± 0.85 (n = 5)	-1.8 ± 4.3	22.3 ± 0.7 (n = 3)
p.Lys450*	0.62 ± 0.27 * (n = 19)	81.9 ± 11.2	290 ± 37 **	70.1 ± 4.2 * (n = 20)	89.1 ± 17.2 (n = 10)	-60.4 ± 3.7 **	8.64 ± 1.42 * (n = 10)	+20.8 ± 6.9 **	36.6 ± 4.2 ** (n = 11)
p.Thr516Ser	38.5 ± 14.3 * (n = 13)	54.6 ± 5.6 *	202 ± 27	72.0 ± 2.6 (n = 13)	33.0 ± 6.7 (n = 8)	-49.7 ± 2.2	5.28 ± 0.56 (n = 5)	-5.5 ± 2.4	19.4 ± 1.7 (n = 5)
p.Arg536Gly	17.7 ± 9.5 (n = 6)	42.2 ± 5.4 **	72.1 ± 8.1 *	65.0 ± 16.5 * (n = 6)	13.7 ± 1.1 (n = 4)	-45.8 ± 0.6	5.37 ± 0.90 (n = 4)	+2.7 ± 1.7	21.2 ± 1.7 (n = 4)
p.Asn578Ile	24.4 ± 11.9 (n = 6)	62.6 ± 7.3	194 ± 117	87.4 ± 13.7 (n = 6)	20.6 ± 3.3 (n = 4)	-48.6 ± 3.9	6.68 ± 2.48 (n = 4)	-1.4 ± 2.6	24.5 ± 1.2 (n = 5)

Table S5. Biophysical parameters for Kv4.1 wild-type and variant binary channels with KChIP

Kv4.1 channels (grey, variant channels with red or purple dots) were studied in the presence of KChIP (orange). Mean values ± SD and number of observations (n) are given for the peak current amplitude at + 40 mV (I_{+40}), the time constants of macroscopic inactivation (τ_1 and τ_2) including the fractional amplitude of τ_1 (%), the time constant of recovery from inactivation (τ_{rec}) and the voltages of halfmaximal inactivation ($V_{1/2,inact}$) and activation ($V_{1/2,act}$) with corresponding slope factors (k_{inact} and k_{act}, respectively); Statistics are based on one-way analysis of variance (ANOVA) with Dunnett's post hoc testing. For significant variant effects (i.e., deviations from Kv4.1 WT + KChIP; key variants: red, maternally inherited missense variants: purple) significance levels are indicated with * (p<0.05) or ** (p<0.0001); shaded fields: no significant differences found compared to Kv4.1 WT + KChIP.

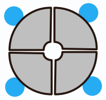

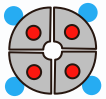

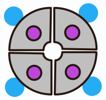

Heterologous expression:

**Kv4.1 + DPP**	I_{+40} (µA)	τ_1 (ms)	τ_2 (ms)	% τ_1	τ_{rec} (ms)	$V_{1/2,inact}$ (mV)	k_{inact} (mV)	$V_{1/2,act}$ (mV)	k_{act} (mV)
WT	62.8 ± 23.3 (n = 5)	26.3 ± 3.8	289 ± 59	71.7 ± 2.7 (n = 11)	29.7 ± 8.4 (n = 5)	-71.9 ± 3.9	8.46 ± 2.63 (n = 4)	-22.4 ± 4.8	24.7 ± 4.6 (n = 5)
p.Thr57Met	44.6 ± 11.2 (n = 6)	35.8 ± 2.9 **	279 ± 27	67.1 ± 2.5 * (n = 13)	45.8 ± 11.6 (n = 7)	-74.8 ± 1.2	7.03 ± 0.76 (n = 7)	-23.2 ± 3.3	21.9 ± 2.7 (n = 9)
p.Arg60Cys	70.9 ± 20.3 (n = 6)	21.5 ± 1.9	305 ± 15	75.8 ± 1.8 * (n = 11)	34.1 ± 3.3 (n = 4)	-73.4 ± 3.3	6.17 ± 0.77 (n = 5)	-24.2 ± 6.0	19.8 ± 5.9 (n = 6)
p.Gly80Arg	57.1 ± 17.9 (n = 6)	19.2 ± 2.0 *	322 ± 20	74.2 ± 3.0 (n = 11)	27.9 ± 6.4 (n = 5)	-73.8 ± 1.8	5.43 ± 0.66 (n = 5)	-28.9 ± 3.5	19.2 ± 3.6 (n = 5)
p.Arg92Cys	9.23 ± 3.65 ** (n = 13)	33.0 ± 4.2 *	330 ± 43	68.9 ± 3.3 (n = 12)	39.1 ± 4.8 (n = 9)	-67.3 ± 4.1	9.41 ± 1.79 (n = 9)	-1.5 ± 14.2 *	27.7 ± 4.4 (n = 8)
p.Arg107Gln	60.7 ± 26.6 (n = 6)	24.2 ± 4.6	252 ± 33	76.8 ± 3.1 ** (n = 18)	19.8 ± 1.9 (n = 4)	-57.9 ± 8.6 **	12.1 ± 2.9 * (n = 4)	-15.8 ± 7.7	25.3 ± 4.7 (n = 7)
p.Asp115Asn	48.2 ± 17.5 (n = 6)	23.4 ± 2.8	350 ± 12 *	77.2 ± 2.1 ** (n = 10)	28.3 ± 3.8 (n = 4)	-72.8 ± 4.3	10.0 ± 2.0 (n = 4)	-17.5 ± 12.6	27.7 ± 5.1 (n = 4)
p.Arg146Cys	18.2 ± 2.0 ** (n = 6)	17.9 ± 2.1 *	359 ± 41 **	85.9 ± 1.0 ** (n = 10)	26.1 ± 4.2 (n = 4)	-73.8 ± 1.3	7.45 ± 0.43 (n = 4)	-24.7 ± 4.6	18.1 ± 3.7 (n = 6)
p.Ala148Thr	38.5 ± 12.2 * (n = 6)	24.1 ± 3.9	372 ± 41 **	80.6 ± 3.0 ** (n = 10)	24.6 ± 4.9 (n = 4)	-73.2 ± 3.4	7.68 ± 0.14 (n = 4)	-24.4 ± 5.4	20.3 ± 5.2 (n = 6)
p.Ala202Thr	48.8 ± 8.6 (n = 6)	35.7 ± 5.3 **	313 ± 28	69.3 ± 2.2 (n = 16)	48.6 ± 6.6 (n = 8)	-72.9 ± 1.3	8.03 ± 0.50 (n = 8)	-17.9 ± 4.2	20.8 ± 3.2 (n = 10)
p.His308Tyr	0.82 ± 0.40 ** (n = 14)	47.6 ± 3.0 **	317 ± 50	64.5 ± 3.5 ** (n = 14)	2420 ± 1350 ** (n = 8)	-110.3 ± 6.5 **	6.74 ± 3.56 (n = 8)	-17.2 ± 14.3	30.0 ± 8.0 (n = 10)
p.Arg431Cys	65.7 ± 19.9 (n = 6)	17.8 ± 1.2 *	283 ± 32	81.4 ± 1.8 ** (n = 11)	20.6 ± 1.8 (n = 4)	-71.5 ± 2.0	7.68 ± 1.45 (n = 4)	-24.3 ± 5.3	20.6 ± 5.8 (n = 6)
p.Lys450*	1.27 ± 0.54 ** (n = 10)	41.9 ± 12.1 **	279 ± 36	71.6 ± 3.6 (n = 14)	75.7 ± 5.4 (n = 7)	-80.2 ± 2.5 *	7.97 ± 1.06 (n = 7)	-23.7 ± 7.5	25.6 ± 4.3 (n = 8)
p.Thr516Ser	47.8 ± 13.6 (n = 11)	33.9 ± 3.8 *	320 ± 23	67.4 ± 2.4 * (n = 11)	50.8 ± 6.4 (n = 6)	-70.2 ± 2.6	6.38 ± 0.23 (n = 8)	-16.6 ± 2.6	22.0 ± 2.0 (n = 7)
p.Arg536Gly	52.5 ± 10.1 (n = 4)	16.6 ± 1.8 *	272 ± 23	82.2 ± 1.8 ** (n = 9)	17.7 ± 1.9 (n = 4)	-70.0 ± 1.2	5.58 ± 0.52 (n = 4)	-25.7 ± 2.8	17.8 ± 1.5 (n = 4)
p.Asn578Ile	46.1 ± 6.0 (n = 4)	23.0 ± 3.8	332 ± 21 *	74.7 ± 2.8 (n = 10)	48.0 ± 8.3 (n = 3)	-78.1 ± 1.8	6.36 ± 0.76 (n = 4)	-31.7 ± 3.9	18.6 ± 5.3 (n = 5)

Table S6. Biophysical parameters for Kv4.1 wild-type and variant binary channels with DPP

Kv4.1 channels (grey, variant channels with red or purple dots) were studied in the presence of DPP (blue). Mean values ± SD and number of observations (n) are given for the peak current amplitude at + 40 mV (I_{+40}), the time constants of macroscopic inactivation (τ_1 and τ_2) including the fractional amplitude of τ_1 (%), the time constant of recovery from inactivation (τ_{rec}) and the voltages of halfmaximal inactivation ($V_{1/2,inact}$) and activation ($V_{1/2,act}$) with corresponding slope factors (k_{inact} and k_{act}, respectively); Statistics are based on one-way analysis of variance (ANOVA) with Dunnett's post hoc testing. For significant variant effects (i.e., deviations from Kv4.1 WT + DPP; key variants: red, maternally inherited missense variants: purple) significance levels are indicated with * (p<0.05) or ** (p<0.0001).

		Kv4.1 functional analysis																													
		peak current amplitude						macroscopic inactivation						recovery from inactivation						voltage dependence of steady-state inactivation						voltage dependence of activation					
Individual #		t	a	K	D	= ± K	= ± D	t	a	K	D	= ± K	= ± D	t	a	K	D	= ± K	= ± D	t	a	K	D	= ± K	= ± D	t	a	K	D	= ± D	PS3 score
	**DNVs**																														
1	c.274C>T (p.Arg92Cys)	Y	Y	Y	Y	Y	N	Y	Y	Y	Y	N	N	N	N	N	N	N	N	N	Y	N	N	N	N	Y	Y	Y	Y	Y	15
2	c.343G>A (p.Asp115Asn)	Y	N	N	N	Y	N	Y	Y	Y	Y	N	N	N	N	N	N	N	N	N	Y	Y	N	N	Y	N	Y	Y	N	Y	12
	**PTVs**																														
3	c.182_194del (p.Tyr61Cysfs*31)	complete LOF																													
4	c.295C>T (p.Arg99*)	complete LOF																													
5	c.1348A>T (p.Lys450*)	Y	Y	Y	Y	N	N	Y	Y	Y	Y	N	N	N	N	N	N	Y	N	Y	N	Y	Y	Y	N	N	Y	Y	N	N	15
	**maternally inherited missense variants**																														
	variants affecting the N-terminal cytoplasmic domain																														
6	c.170C>T (p.Thr57Met)	N	N	N	N	N	N	N	Y	Y	Y	N	N	N	N	N	N	N	N	N	Y	N	N	N	N	N	Y	N	N	N	5
7	c.178C>T (p.Arg60Cys)	N	N	N	N	N	N	N	N	N	Y	N	N	N	N	N	N	N	N	N	N	N	N	N	N	N	N	N	N	N	1
8	c.238G>C (p.Gly80Arg)	N	Y	N	N	N	N	N	Y	Y	Y	N	N	N	N	N	N	N	N	N	Y	Y	N	N	N	N	N	N	N	N	6
9	c.320G>A (p.Arg107Gln)	N	N	N	N	N	N	Y	N	N	Y	N	N	N	N	N	N	N	N	N	N	N	Y	N	Y	N	N	N	N	N	4
10	c.436C>T (p.Arg146Cys)	Y	N	Y	Y	Y	N	Y	Y	Y	Y	N	N	N	N	N	N	N	N	N	Y	N	N	N	N	N	Y	N	N	N	10
11	c.442G>A (p.Ala148Thr)	N	N	N	Y	N	N	Y	Y	N	Y	N	N	N	N	N	N	N	N	N	Y	N	N	N	Y	N	N	N	N	N	6
	variants affecting transmembrane domains																														
12	c.604G>A (p.Ala202Thr)	N	Y	Y	N	N	N	N	Y	Y	Y	N	N	N	N	N	N	N	N	Y	Y	N	N	N	N	Y	N	N	N	N	8
13	c.922C>T (p.His308Tyr)	Y	Y	N	Y	N	Y	Y	Y	Y	Y	N	Y	Y	Y	Y	Y	N	Y	Y	Y	Y	Y	N	N	Y	Y	Y	N	Y	22
	variants affecting the C-terminal cytoplasmic domain																														
14	c.1291C>T (p.Arg431Cys)	N	Y	Y	N	N	N	N	N	Y	Y	N	N	N	N	N	N	N	N	N	Y	N	N	N	Y	N	N	N	N	N	6
15 & 16	c.1546A>T (p.Thr516Ser)	N	Y	Y	N	N	N	Y	N	Y	Y	N	N	N	N	N	N	N	N	Y	Y	N	N	N	N	Y	N	N	N	N	8
17	c.1606A>G (p.Arg536Gly)	N	N	N	N	N	N	N	Y	Y	Y	N	N	N	N	N	N	N	N	N	N	N	N	N	N	N	N	N	N	N	3
18	c.1733A>T (p.Asn578Ile)	N	Y	N	N	N	N	Y	Y	N	Y	N	N	N	N	N	N	N	N	N	Y	N	N	N	N	N	N	N	N	N	5

Table S7. PS3 scoring for functionally expressed *KCND1* variants

Functional *KCND1* variants were assessed based on significant alterations relative to Kv4.1 WT. For each biophysical parameter and in all channel configurations (t: ternary, a: alone, K: only with KChIP, D: only with DPP) the following questions were asked: 1) Is there a significant difference relative to Kv4.1 WT? Yes (Y) or No (N); 2) Is the β-subunit effect on this biophysical parameter absent (i.e.; no KChIP effect, = ± K or no DPP effect, = ± D)? Yes (Y) or No (N). Assessment based on the data from Tables S3 - S6; see also Figures S3 - S5.

Supplemental References

1. Kise, Y., Kasuya, G., Okamoto, H.H., Yamanouchi, D., Kobayashi, K., Kusakizako, T., Nishizawa, T., Nakajo, K., and Nureki, O. (2021). Structural basis of gating modulation of Kv4 channel complexes. Nature *599*, 158-164.

2. An, W.F., Bowlby, M.R., Betty, M., Cao, J., Ling, H.P., Mendoza, G., Hinson, J.W., Mattsson, K.I., Strassle, B.W., Trimmer, J.S., and Rhodes, K.J. (2000). Modulation of A-type potassium channels by a family of calcium sensors. Nature *403*, 553-556.

3. Nadal, M.S., Ozaita, A., Amarillo, Y., de Miera, E.V., Ma, Y., Mo, W., Goldberg, E.M., Misumi, Y., Ikehara, Y., Neubert, T.A., and Rudy, B. (2003). The CD26-related dipeptidyl aminopeptidase-like protein DPPX is a critical component of neuronal A-Type K^+ channels. Neuron *37*, 449-461.
